# Interplay of Stereochemistry and Charge Governs Guest Binding in Flexible Zn^II^_4_L_4_ Cages

**DOI:** 10.1021/jacs.4c12320

**Published:** 2024-11-14

**Authors:** Weichao Xue, Elie Benchimol, Alexandre Walther, Nianfeng Ouyang, Julian J. Holstein, Tanya K. Ronson, Joseph Openy, Yujuan Zhou, Kai Wu, Rituparno Chowdhury, Guido H. Clever, Jonathan R. Nitschke

**Affiliations:** † Key Laboratory of Green Chemistry & Technology of Ministry of Education, College of Chemistry, Sichuan University, 29 Wangjiang Road, Chengdu 610064, China; ‡ Fakultät für Chemie und Chemische Biologie, Technische Universität Dortmund, Otto-Hahn-Strasse 6, Dortmund 44227, Germany; § Yusuf Hamied Department of Chemistry, University of Cambridge, Cambridge CB2 1EW, U.K.; ∥ Cavendish Laboratory, University of Cambridge, Cambridge CB3 0HE, U.K.

## Abstract

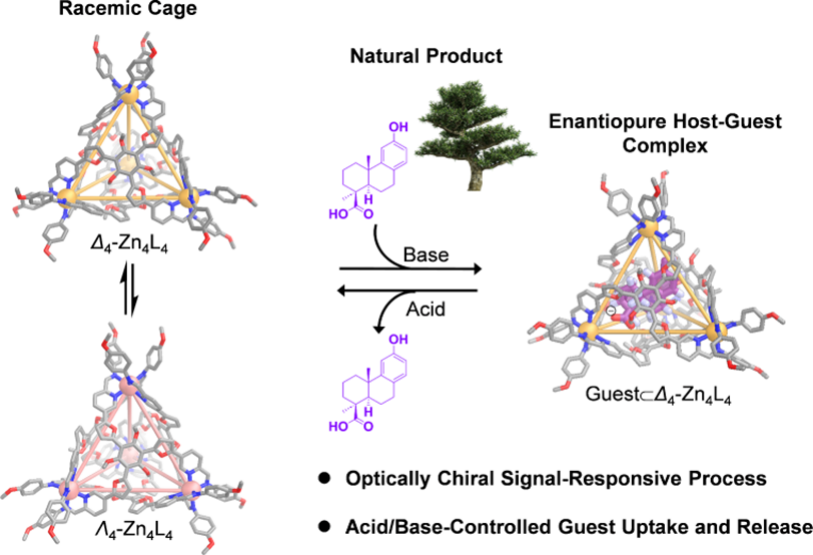

Here, we report the synthesis of a family of chiral Zn^II^_4_L_4_ tetrahedral cages by subcomponent self-assembly. These cages contain a flexible trialdehyde subcomponent that allows them to adopt stereochemically distinct configurations. The incorporation of enantiopure 1-phenylethylamine produced Δ_4_ and Λ_4_ enantiopure cages, in contrast to the racemates that resulted from the incorporation of achiral 4-methoxyaniline. The stereochemistry of these Zn^II^_4_L_4_ tetrahedra was characterized by X-ray crystallography and chiroptical spectroscopy. Upon binding the enantiopure natural product podocarpic acid, the Zn^II^ stereocenters of the enantiopure Δ_4_-Zn^II^_4_L_4_ cage retained their Δ handedness. In contrast, the metal stereocenters of the enantiomeric Λ_4_-Zn^II^_4_L_4_ cage underwent inversion to a Δ configuration upon encapsulation of the same guest. Insights gained about the stereochemical communication between host and guest enabled the design of a process for acid/base-responsive guest uptake and release, which could be followed by chiroptical spectroscopy.

## Introduction

Chiral signal transduction is a fundamental process in living systems, where enzymes and bioreceptors dynamically adapt their stereochemistry to enantiospecifically catalyze reactions and stereoselectively recognize their targets.^1,2^ Methods for designing sophisticated supramolecular systems with rich stereochemical information and novel structural adaptability are therefore of interest, not only to illuminate the flow of stereochemical information at the molecular level but also to enable bioinspired applications.^3−8^

In analogy to bioreceptors, enantiopure metal–organic cages^9^ have exhibited functions such as enantioselective catalysis,^10^ recognition,^11^ separation^12^ and chiroptical readout^13^ owing to their well-defined chirotoptic cavities.^14^ Their construction requires a source of stereochemical information, such as from chiral ligands during self-assembly, or postassembly chiral resolution of racemic cages using enantiopure counterions or guests.^9^ The incorporation of commercially available chiral building blocks into structures built using subcomponent self-assembly is a particularly useful technique,^9b^ as stereochemistry can efficiently be transferred from less-expensive subcomponents to the final structures, resulting in the modular design of enantiopure metal–organic helicates,^15^ tetrahedra^16^ and cubes.^17^

Stereochemical communication between enantiopure cages and chiral guests has been fruitfully investigated.^9^ Many studies have examined the stereochemical impact of the host on the guest, particularly the ability of cages to enantioselectively bind one enantiomer of a guest over the other.^11,12^ This capability allows chiral cages to recognize artificial molecules, such as binaphthols^11c,12^ and cryptophanes,^18^ as well as biomolecules, including amino acids^19^ and steroids.^20^ There are fewer studies of the impacts of enantiopure guests on the stereochemistry of hosts.^13,21^ Biologically active natural products often have low symmetry due to multiple stereogenic centers. One approach to binding them would be to employ a host capable of dynamically adapting its stereochemistry.

**Figure 1:**
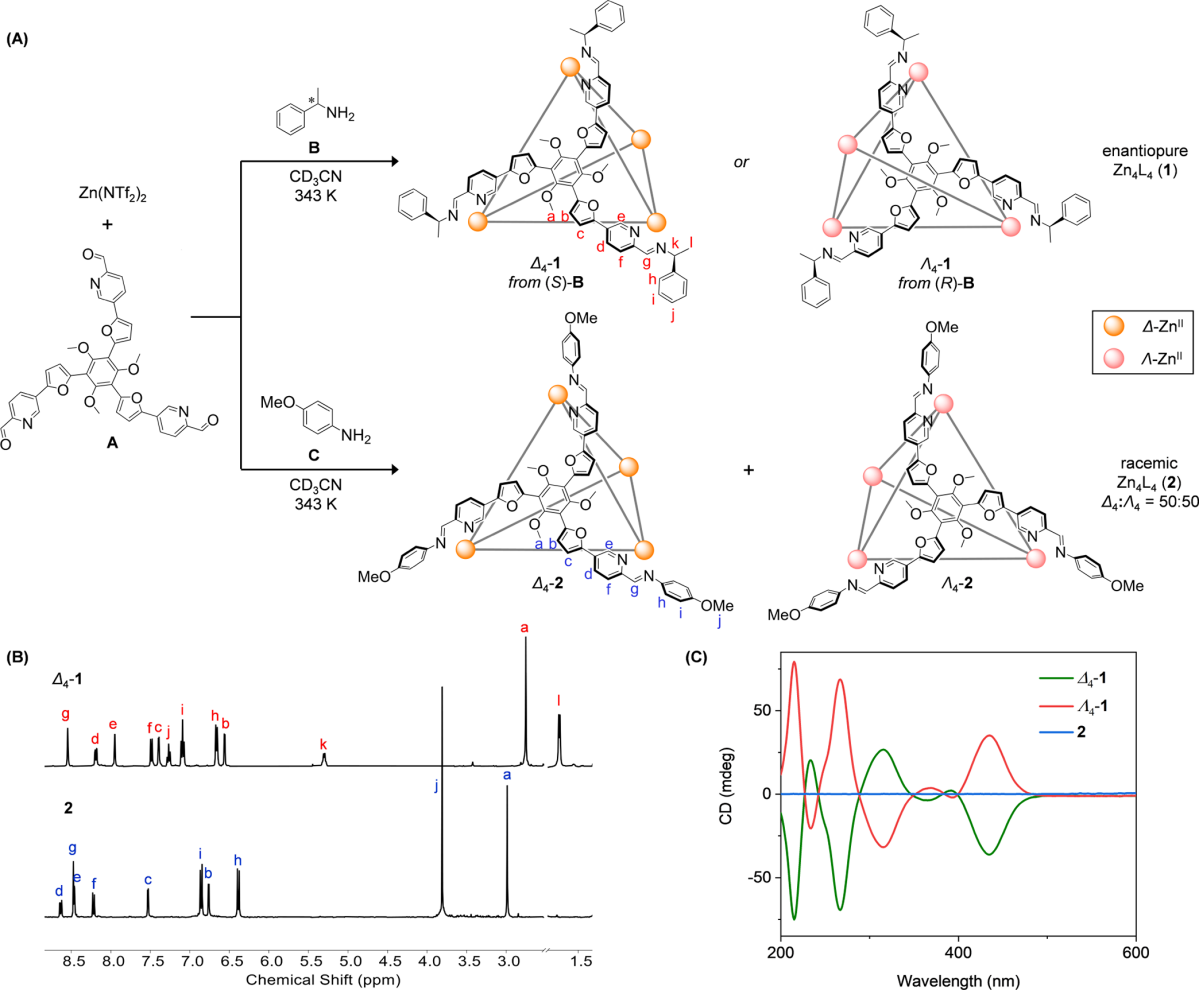
(A) Subcomponent self-assembly of the enantiomers Δ_4_-**1** and Λ_4_-**1**, and racemic **2**. (B) Partial ^1^H NMR spectra of Δ_4_-**1** and **2** (500 MHz, CD_3_CN, 298 K). (C) CD spectra of Δ_4_-**1**, Λ_4_-**1**, and **2** in acetonitrile at equal concentrations (50 μM).

Here we describe the preparation of a family of Zn^II^_4_L_4_ tetrahedra by subcomponent self-assembly from a flexible trialdehyde panel **A** (Figure 1A). With **A** and Zn^II^, enantiopure subcomponent 1-phenylethylamine **B** produced enantiopure Δ_4_-**1** or Λ_4_-**1**, depending on the handedness of 1-phenylethylamine, whereas achiral 4-methoxyaniline **C** gave rise to racemic cage **2**. Upon binding the low-symmetry natural product, podocarpic acid, either Δ_4_-**1** or racemic **2** gave a host–guest complex with all Δ stereochemistry at Zn^II^. In contrast, the handedness of the metal vertices of Λ_4_-**1** Zn^II^_4_L_4_ inverted upon encapsulation of podocarpic acid.

## Results and Discussion

### Preparation and Characterization of Cages

Tritopic formylpyridine subcomponent **A**, featuring a 1,3,5-tris(2-furyl)-2,4,6-trimethoxybenzene panel, was chosen because of its structural flexibility. Cages incorporating this panel have exhibited conformational plasticity upon guest binding, as demonstrated in Fujita’s knotted palladium(II) cage^22^ and our tetrahedral iron(II) cage.^23^ Enantiopure 1-phenylethylamine subcomponent **B** was selected due to its ability to dictate the handedness of a neighboring metal ion in both mononuclear^24^ and multinuclear^15−17,20d^ complexes.

The reaction of subcomponents **A** (4 equiv) and enantiopure **B** (12 equiv) with zinc(II) bis(trifluoromethanesulfonyl)imide (Zn^II^(NTf_2_)_2_, 4 equiv) in CD_3_CN at 343 K afforded cage **1** (Figure 1A) as the uniquely observed product, with electrospray ionization mass spectrometry (ESI-MS) confirming its Zn^II^_4_L_4_ composition (Figures S11 and S12). The identical ^1^H NMR spectra of **1** prepared from (*S*)-**B** and (*R*)-**B** indicated formation of the enantiomers of **1** (Figures 1B and S15), consistent with circular dichroism (CD) spectra showing mirror-image bisignate CD curves (Figure 1C). The ^1^H NMR spectra indicated the formation of a *T*-symmetric tetrahedral framework, where four octahedral tris-chelated Zn^II^ centers adopt the same handedness (Δ_4_-**1** or Λ_4_-**1**), and the ligands adopt a 3-fold-symmetric configuration.

Achiral 4-methoxyaniline subcomponent **C** likewise self-assembled with **A** and Zn(NTf_2_)_2_, resulting in the formation of cage **2**, with its Zn^II^_4_L_4_ composition confirmed by ESI-MS and *T* point symmetry by its ^1^H NMR spectrum (Figures S17 and 1B). Racemic **2** exhibited a flat solution CD spectrum (Figure 1C), consistent with the presence of an equimolar ratio of its Δ_4_-**2** and Λ_4_-**2** enantiomers, as is frequently observed in cages formed by metal ions with octahedral coordination geometries.^9b,12a,25^

**Figure 2:**
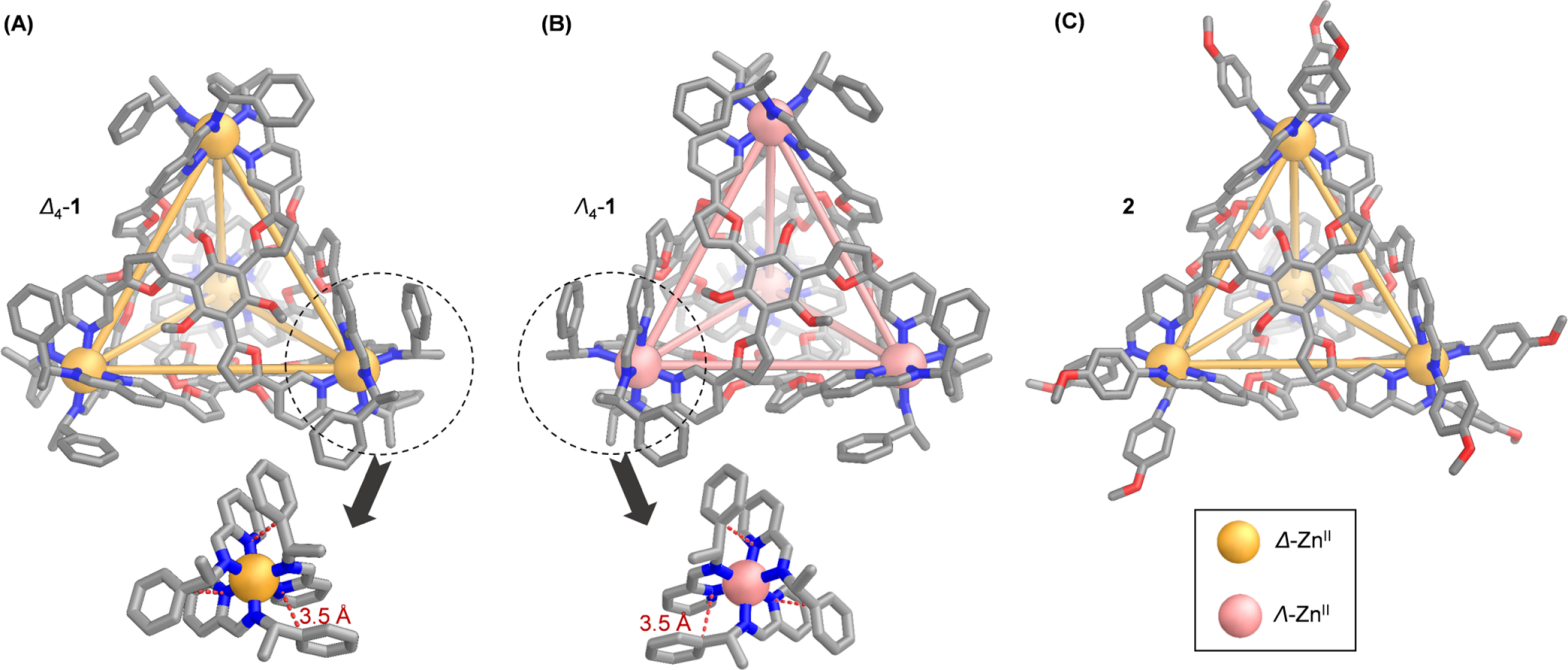
(A) Crystal structure of Δ_4_-**1**, with a single (*S*,*S*,*S*)-Δ-Zn center highlighted. (B) Crystal structure of Λ_4_-**1**, with a single (*R*,*R*,*R*)-Λ-Zn center highlighted. (C) Crystal structure of **2**. Disorder, anions and hydrogen atoms are omitted for clarity.

Slow vapor diffusion of diisopropyl ether into acetonitrile solutions of Zn^II^_4_L_4_ cages **1** and **2** led to crystals suitable for single-crystal X-ray diffraction analysis using synchrotron radiation. The solid-state structure of each Zn^II^_4_L_4_ cage adopted a tetrahedral architecture, with four Zn^II^ centers capped by four ligand faces (Figure 2). All structures exhibited idealized *T* symmetry, in line with the solution-state ^1^H NMR spectra displaying one set of ligand peaks.

Both Δ_4_ and Λ_4_ enantiomers of **1** crystallized in the chiral *R*3 space group, allowing for the determination of the absolute configuration in both cases (Figure 2A,B). Subcomponent (*S*)-**B** dictated Δ handedness of the Zn^II^ centers to form (*S*)_12_-Δ_4_-**1**, whereas (*R*)-**B** produced (*R*)_12_-Λ_4_-**1**.^17,20c,24^ The transfer of stereochemical information from subcomponent **B** to the metal centers is facilitated by aromatic stacking interactions around Zn^II^ centers, as observed in the crystal structures. Around each Zn^II^ center within (*S*)_12_-Δ_4_-**1**, three phenyl rings of (*S*)-**B** stack with pyridine rings of **A** at a distance of 3.5 Å, thus providing the stereocontrol required to establish Δ stereochemistry. (*R*)-**B** likewise dictates Λ configuration through the same aromatic stacking interactions.

Within Δ_4_-**1**, four ligand faces adopted an anticlockwise orientation to pair with Δ Zn^II^ centers, whereas a clockwise ligand orientation was observed for Λ_4_-**1**. The mean Zn^II^···Zn^II^ distances are 14.5 Å for both enantiomers; cavity volumes of Δ_4_-**1** and Λ_4_-**1** were calculated to be of 354 Å^3^ and 352 Å^3^ (Figure S67), respectively, using the MoloVol program.^26^ Structural parameters for both enantiomers are identical within the margin of error. Racemic cage **2** crystallized in the *C*2/c space group, with Δ_4_-**2** and Λ_4_-**2** related by inversion in the crystal. An average Zn^II^···Zn^II^ distance of 14.5 Å and a cavity volume of 342 Å^3^ were calculated for **2** (Figures 2C and S67).

### Host–Guest Studies

Based on the well-enclosed cavities and well-defined stereochemistry in the crystal structures of Δ_4_-**1** and Λ_4_-**1**, we inferred that they might host chiral guests of suitable sizes. Host–guest interactions were gauged by NMR, ESI-MS and chiroptical spectroscopy, with binding affinities quantified by ^1^H NMR titrations (Table 1),^27^ as detailed in Section S4.

**Table 1:**
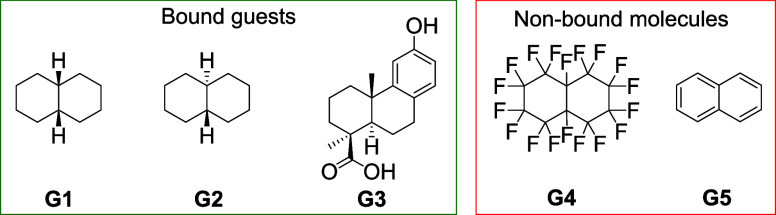
Host-Guest Properties of Cages 1 and 2

Guest^a^	*K*_a_ for Δ_4_-**1** (× 10^2^ M^–1^)	*K*_a_ for Λ_4_-**1** (× 10^2^ M^–1^)	*K*_a_ for **2** (× 10^2^ M^–1^)
**G1**	1.08 ± 0.07	1.10 ± 0.07	0.40 ± 0.10
**G2**	0.48 ± 0.04	0.47 ± 0.04	0.35 ± 0.10
**G3**	1.98 ± 0.08	1.44 ± 0.08	–
**G3** carboxylate^b^	28.7 ± 1.0	21.3 ± 1.0	17.5 ± 1.0

a Titrations were carried out by ^1^H NMR through the addition of a CD_3_CN solution of the prospective guest or portion-wise addition as a solid into a stock solution of the host in CD_3_CN (0.75 mM), using 1,3,5-trimethoxybenzene or 1,3,5-trimethybenzene as an internal standard.

b The conjugate base of **G3** was prepared by mixing equimolar amounts of **G3** and *N*,*N*-diisopropylethylamine (DIPEA) in CD_3_CN.

Cage **1** was observed to bind decalin (169 Å^3^)^28^ within its 354 Å^3^ cavity in slow exchange on the NMR time scale, with proton signals of the bound guest shifted upfield as a result of aromatic shielding effects, consistent with internal guest binding (Figures S24 and S27). Despite the slow exchange, NMR titrations determined the binding constant to be small. A large excess of the guest was thus required to shift the equilibrium toward the formation of a clean host–guest complex. Nuclear Overhauser effect spectroscopy (NOESY) cross peaks between the bound **G1** (Table 1) signals and the methoxy and pyridine signals of Δ_4_-**1** were clearly observed (Figure S26). Both cage enantiomers showed identical binding behavior toward *cis*-decalin **G1** and *trans*-decalin **G2** (Table 1, entries 1 and 2), as expected given the achiral nature of **G2** and the rapid racemization of **G1** at room temperature. **G1** was observed to be bound more strongly than **G2**. This difference was inferred to be due to the more spherical configuration of *cis*-**G1**, which may better fit the near-spherical cage cavity.^29^ In contrast, Δ_4_-**1** did not encapsulate perfluorodecalin **G4** or naphthalene **G5**, suggesting that aliphatic CH-π interactions and CH···O hydrogen bonds between host and guest might contribute to binding.

Given the prevalence of the decalin motif in natural products,^30^ we investigated stereochemical communication between enantiopure **1** and low-symmetry natural products containing the decalin motif.^31^ Larger guests, such as progesterone (312 Å^3^) and andrographolide (326 Å^3^), were not bound by **1**.^28^ However, cage **1** (354 Å^3^) was observed to encapsulate podocarpic acid **G3** (268 Å^3^), which has been shown to interact with protein signaling pathways implicated in disease states.^32^**G3** was observed to bind in slow exchange on the NMR time scale, showing shielding effects consistent with internal binding (Figure S34). The formation of **G3**⊂**1** was also observed by ESI-MS (Figures S36 and S42), consistent with a ^1^H diffusion-ordered spectroscopy (DOSY) spectrum displaying the same diffusion coefficients for host and bound guest signals (Figures S38 and S44). The stronger binding affinity of **G3** over **G1** and **G2** was attributed to aromatic stacking between the phenyl ring of **G3** and the pyridine rings of cage **1**, as indicated by the NOE correlations. Cage **1** also bound the conjugate base of **G3** with a stronger binding affinity than for neutral **G3** (Table 1, entries 3 and 4); we infer that Coulombic interaction between cationic host and anionic guest increase the binding affinity.

### Stereochemical Communication

**Figure 3:**
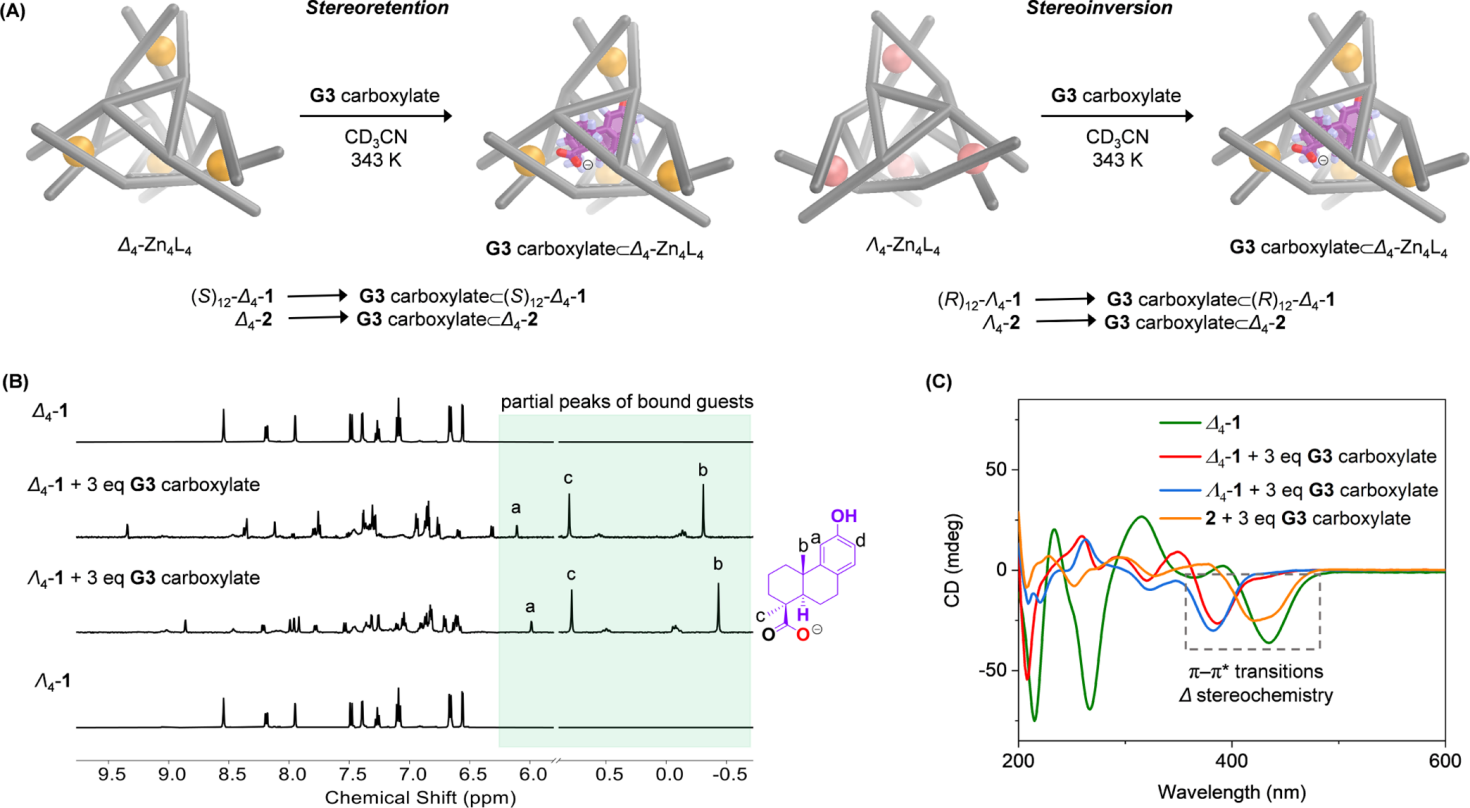
(A) Schematic representation showing stereochemical communication between Zn^II^_4_L_4_ and **G3** carboxylate. (B) Partial ^1^H NMR spectra of Δ_4_-**1**, Λ_4_-**1**, and their host–guest complexes, with peaks for the bound guest highlighted against a light green background (500 MHz, CD_3_CN, 298 K). (C) CD spectra of Δ_4_-**1** and host–guest complexes in acetonitrile, all at 50 μM cage concentration, with π–π* transitions indicative of Δ stereochemistry highlighted in a gray dotted box.

Both Δ_4_-**1** and Λ_4_-**1** bound enantiopure **G3** carboxylate, forming host–guest complexes with distinct ^1^H NMR signals, consistent with the formation of diastereomeric complexes (Figure 3A,B). As expected, these two diastereomeric complexes displayed different NOE correlations between the bound guest and host. For Δ_4_-**1**, cross peaks were observed between the aromatic protons H_a_ and H_d_ of the **G3** carboxylate and the aromatic protons of the host (Figure S40), while, for Λ_4_-**1**, its aromatic protons correlated with both the methyl proton H_b_ and aromatic proton H_a_ of the bound guest (Figure S46). In both cases, the host signals split and broadened after guest encapsulation, consistent with desymmetrization of the host framework on the NMR time scale. The overlap of proton signals prevented the assignment of each peak. The desymmetrization of the cage framework was inferred to be a result of slow tumbling of the guest within the cage cavity.^20c^ In contrast, a single set of well-defined proton peaks was observed for the bound guest, indicating that both host–guest complexes are enantiopure.

Although numerous attempts to grow host–guest crystals suitable for X-ray diffraction were unsuccessful, CD and circularly polarized luminescence (CPL) provided insights into the stereochemical consequences of host–guest binding in solution. The CD spectra of Δ_4_-**1** and Λ_4_-**1** displayed strong Cotton effects between 400–485 nm (Figure 1c), corresponding to π–π* transitions. As noted by Scott et al.,^24^ a negative band in this region correlates with Δ metal centers, whereas a positive band indicates Λ stereochemistry. Host–guest complexes of **G3** carboxylate prepared from both Δ_4_-**1** and Λ_4_-**1** showed negative bands in the π–π* region (Figures 3C and S48). Λ_4_-**1** also exhibited a positive circularly polarized emission band in the 400–600 nm range ascribed to π–π* transitions of the ligand backbone upon excitation,^33^ whereas a negative band was observed for Δ_4_-**1** (Figure S65). Host–guest complexes of **G3** carboxylate bound by Λ_4_-**1** and Δ_4_-**1** likewise showed negative CPL bands in this region (Figure S66). Based upon these CD and CPL results, we thus conclude that the Zn^II^_4_L_4_ framework of both host–guest complexes adopted Δ metal stereochemistry. (*S*)_12_-Δ_4_-**1** thus bound **G3** carboxylate in a stereoretentive manner, producing **G3** carboxylate⊂(*S*)_12_-Δ_4_-**1**, whereas (*R*)_12_-Λ_4_-**1** inverted the stereochemistry of its Zn^II^ centers and ligand panel orientations upon encapsulating **G3** carboxylate, giving **G3** carboxylate⊂(*R*)_12_-Δ_4_-**1**.^34^

To gain thermodynamic insights into the interplay between stereochemistry and binding, we conducted isothermal titration calorimetry (ITC) experiments (Section S4.4). The binding of **G3** carboxylate by both Δ_4_-**1** and Λ_4_-**1** was observed to be driven by both enthalpy and entropy. Binding values of Δ*H* = −22.6 ± 4.4 kJ mol^–1^ and Δ*S* = 79.9 J mol^–1^ K^−1^ were determined for Δ_4_-**1**, while Λ_4_-**1** exhibited a similar Δ*H* of −23.4 ± 3.3 kJ mol^–1^ and Δ*S* of 72.8 J mol^–1^ K^−1^. The free energies of binding (Δ*G*) at 298 K were thus calculated to be −46.4 ± 4.4 kJ mol^–1^ and −45.2 ± 3.3 kJ mol^–1^ for Δ_4_-**1** and Λ_4_-**1**, respectively. Such small energy differences were consistent with the thermodynamically favorable inversion of the configuration of Λ_4_-**1** upon binding **G3** carboxylate to form the stable **G3** carboxylate⊂Δ_4_-Zn_4_L_4_ complex. We infer this complex to be stabilized by multiple noncovalent interactions between the host and guest, including CH-π, CH···O, π–π, and Coulombic interactions.

**Figure 4:**
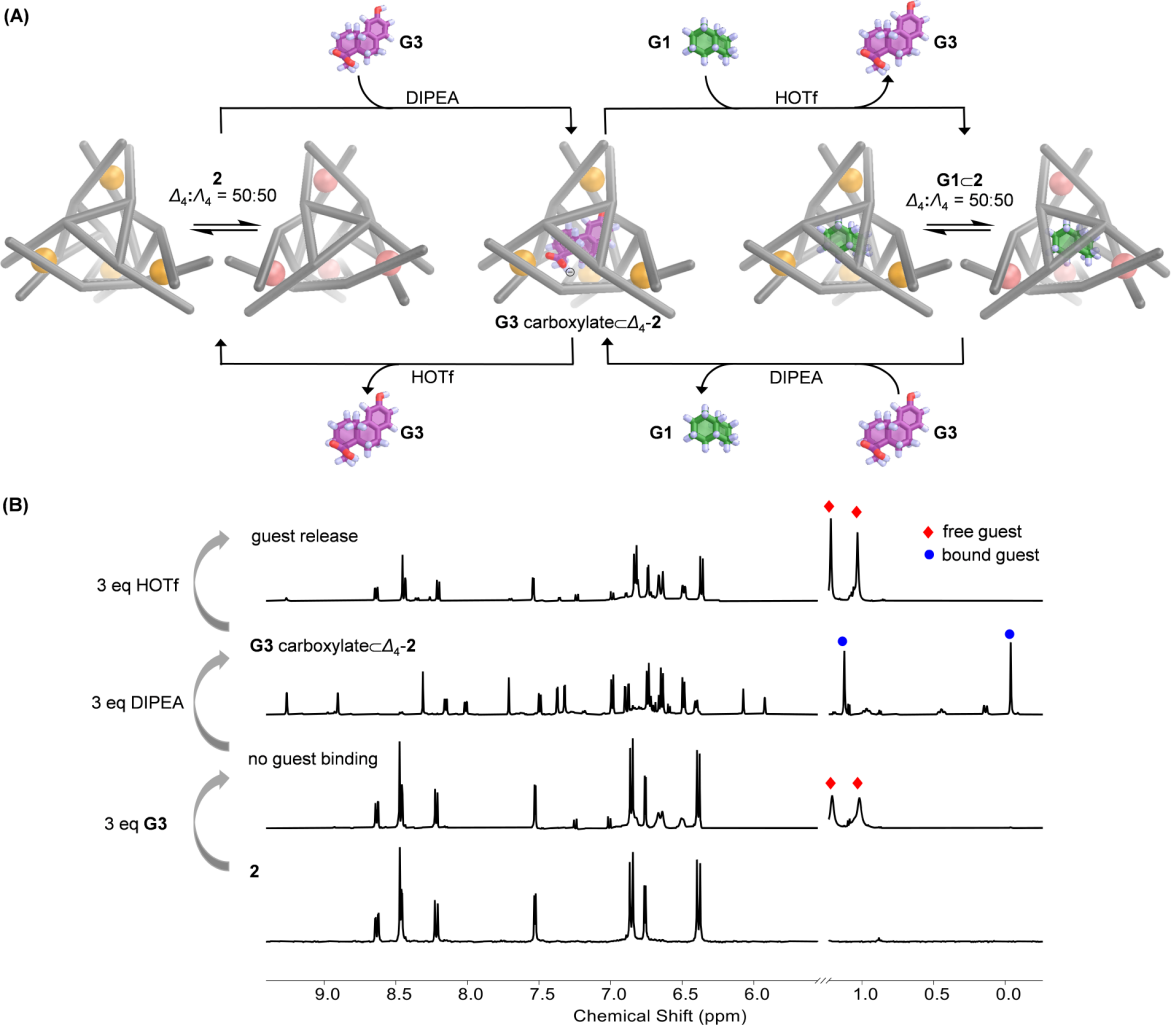
(A) Schematic representation showing the acid/base-controlled guest uptake and release processes involving cage **2**. (B) Partial ^1^H NMR spectra of **2** and its host–guest complexes upon adding guest, acid and base (500 MHz, CD_3_CN, 298 K). Proton signals of the methyl groups from the free and bound guests are labeled with red squares and blue circles, respectively.

Encouraged by this demonstration of stereochemical communication between host **1** and **G3**, we investigated the ability of racemic **2** to bind this enantiopure guest. Cage **2** was not observed to encapsulate neutral **G3**, from which we infer that cage **2**, incorporating 4-methoxyaniline, may be more rigid than phenylethylamine-incorporating **1**, thus leading to insufficient structural adaptability to bind neutral guests. However, cage **2** was observed to bind the conjugate base of **G3**, where electrostatic attraction provides an additional driving force for encapsulation. The observation of a single set of proton peaks for the host and bound guest indicated that **G3** carboxylate⊂**2** formed stereoselectively (Figures 4 and S52). The CD spectrum of this complex again exhibited negative bands for its π–π* transitions (Figures 3C and S55), consistent with the absolute configuration **G3** carboxylate⊂Δ_4_-**2**.

### pH-Driven Guest Uptake and Release

Based upon different binding behaviors toward **G3** and its carboxylate, we designed an acid/base-responsive^35^ guest uptake and release system using cage **2**, as shown in Figure 4A.^36^ The process was monitored by both ^1^H NMR and CD. The addition of **G3** (3 equiv) into a CD_3_CN solution of **2** gave no evidence of guest binding by ^1^H NMR (Figure 4B). As a CD_3_CN solution of DIPEA (3 equiv) was progressively added to the mixture, **G3** carboxylate⊂Δ_4_-**2** formed, as confirmed by ^1^H NMR and CD spectra (Figure S52). Subsequently, progressive addition of a CD_3_CN solution of trifluormethanesulfonic acid (HOTf, 3 equiv) into the mixture resulted in guest release and regeneration of empty **2**. The CD spectrum of this mixture was recorded immediately after guest release, and no Cotton effects were observed for **2** (Figure S55), indicating rapid racemization of the host framework upon guest release.^37^

Acid/base-responsive guest exchange with **G1** was likewise observed (Figures 4A and S53). Adding HOTf to a CD_3_CN solution of **G3** carboxylate⊂Δ_4_-**2** in the presence of excess *cis*-decalin led to the release of **G3** and the simultaneous uptake of **G1**. After guest exchange, **G1**⊂**2** was also observed as the racemate, exhibiting no chiral memory effect (Figure S55). The reformation of enantiopure **G3** carboxylate⊂Δ_4_-**2** subsequently occurred following the addition of DIPEA into the CD_3_CN solution of **G1**⊂**2** containing **G3**.

## Conclusion

The enantiopure metal–organic cages thus prepared have provided a useful framework for the exploration of stereochemical information transfer between host and guest. The chiral guest molecule was able to override the chiral input of the 12 amine subcomponents, leading to an inverted stereochemical output of the host framework. Subtle structural differences between cages **1** and **2** enabled the latter to bind and release enantiopure podocarpic acid driven by a pH swing, where stereochemical imprinting from guest to host provided a readout of guest binding.

Our studies present an example of stereochemical communication between flexible hosts and low-symmetry guests, which stands in contrast to previous studies that focused more generally on stereochemical communication using coordination cages with high-symmetry chiral guests, such as binaphthols^11c,12^ and cryptophanes.^18^ These findings may offer insights for the future design of chiral host systems aimed at binding low-symmetry biologically relevant molecules, with potential applications in drug delivery. Moreover, the phenomenon of pH-driven guest uptake and release may be general to cationic cages for binding guests with acidic protons, which may enable pH-driven chemical purification methods, along with systems providing chiroptical readouts for guest sensing.^38^

## Supporting Information Available

Experimental procedures, NMR characterizations, mass spectrometry data, UV–vis spectra, CD spectra, CPL spectra, volume calculations, and X-ray crystallographic data (PDF)
