# Supplementary material for: Interplay of Stereochemistry and Charge Governs Guest Binding in Flexible ZnII4L4 Cages
Source: J Am Chem Soc. 2024 Nov 14;146(47):32730–7. doi: 10.1021/jacs.4c12320 (PMC11613429; doi:10.1021/jacs.4c12320)
Supplement: Supplementary file 1 — ja4c12320_si_001.pdf [file ja4c12320_si_001.pdf]

Interplay of Stereochemistry and Charge Governs Guest Binding in Flexible $Zn^{II}_4L_4$ Cages

Weichao Xue,[1,2,3,*] Elie Benchimol,[2] Alexandre Walther,[2] Nianfeng Ouyang,[3] Julian J. Holstein,[2] Tanya K. Ronson,[3] Joseph Openy,[2] Yujuan Zhou,[3] Kai Wu,[3] Rituparno Chowdhury,[4] Guido H. Clever,[2,*] and Jonathan R. Nitschke[3,*]

[1]Key Laboratory of Green Chemistry & Technology of Ministry of Education, College of Chemistry, Sichuan University; 29 Wangjiang Road, Chengdu 610064, China

[2]Fakultät für Chemie und Chemische Biologie, Technische Universität Dortmund, Otto-Hahn-Strasse 6, 44227 Dortmund, Germany

[3]Yusuf Hamied Department of Chemistry, University of Cambridge, Cambridge CB2 1EW, UK

[4]Cavendish Laboratory, University of Cambridge, Cambridge CB3 0HE, UK

Email: jrn34@cam.ac.uk; guido.clever@tu-dortmund.de; weichaoxue@scu.edu.cn

Supporting Information

Table of Contents

1 General Information S3

2 Synthesis and Characterization of Subcomponent **A** S5

3 Self-Assembly and Characterization of $Zn^{II}{}_4L_4$ Cages S6

3.1 Self-Assembly of Enantiopure Δ_4-**1** S8

3.2 Self-Assembly of Enantiopure Λ_4-**1** S16

3.3 Self-Assembly of Racemic **2** S18

4 Host-Guest Properties of $Zn^{II}{}_4L_4$ Cages S23

4.1 Host-Guest Interaction of Enantiopure **1** with **G1** and **G2** S25

4.2 Host-Guest Interaction of Enantiopure **1** with **G3** and Its Carboxylate S32

4.3 Host-Guest Interactions of Racemic **2** with **G1**, **G2**, and **G3** Carboxylate S41

4.4 Guest Binding Studies through ITC Experiments S47

5 CPL Studies of Host-Guest Systems S53

6 Volume Calculations S55

7 Crystal Structures of $Zn^{II}{}_4L_4$ Cages S56

7.1 Crystal Structure of Δ_4-**1** S61

7.2 Crystal Structure of Λ_4-**1** S63

7.3 Crystal Structure of **2** S65

8 References S67

1 General Information

Unless otherwise specified, all reagents were purchased from commercial sources and used as received. (*S*)-**B** (*Sigma Aldrich*), (*R*)-**B** (*Sigma Aldrich*), **C** (*Sigma Aldrich*), $Zn(NTf_2)_2$ (*TCI*), *cis*-decalin **G1** (*abcr*), *trans*-decalin **G2** (*abcr*), podocarpic acid **G3** (*Sigma Aldrich*). Compounds **S1**[1] and **S2**[2] were prepared according to reported procedures, respectively. Self-assembly reactions were performed in either CD_3CN or distilled MeCN.

NMR spectra were recorded using the following NMR spectrometers: Bruker 400 MHz Avance III HD smart probe (^{1}H, ^{13}C, and ^{1}H-DOSY), Bruker 500 MHz AVIII HD Smart Probe (^{1}H), Bruker Avance 500 MHz DCH cryoprobe (^{1}H, ^{13}C, and 2D NMR), Bruker Avance 700 MHz TXO cryoprobe (^{1}H, ^{13}C, and 2D NMR), Bruker AV 500 Avance NEO (^{1}H, ^{1}H-DOSY, and 2D NMR), AV 600 MHz Avance III HD (^{1}H, ^{13}C, and 2D NMR). Chemical shifts of the NMR spectra are reported relative to $CDCl_3$ (^{1}H NMR: δ = 7.26 ppm, ^{13}C NMR: δ = 77.0 ppm), CD_3CN (^{1}H NMR: δ = 1.94 ppm, ^{13}C NMR: δ = 118.3 ppm). Data for ^{1}H NMR spectra were reported as follows: chemical shift (ppm), peak shape (s = singlet, d = doublet, t = triplet, m = multiplet, br = broad signal), coupling constant (Hz), and integration. Data for ^{13}C NMR were reported with chemical shift (ppm) values referenced to the residual solvent peak.

UV-vis measurements were employed to fine-tune the solution concentration for subsequent CD measurements, and were performed on a Varian Cary 400 scan UV-vis spectrophotometer or a DAD HP-8453 UV-Vis spectrometer with a 1 mm path-length cuvette at 25 °C.

Circular Dichroism was performed on an Applied-Photophysics Chirascan CD spectrometer using a 1 mm path-length cuvette. Experiments were recorded at 298 K, maintained with a Peltier temperature control. Measurements were background subtracted from blank solvent in an identical cuvette. The sample concentrations were adjusted to maintain a HV below 800 V.

Circularly polarized luminescence (CPL) measurements were performed on a JASCO CPL-300 spectrophotometer, equipped with a (150 W) Xe lamp as light source, using a 10 mm path-length cuvette. The CPL spectra of cages and host-guest complexes were recorded with an excitation and emission bandwidth of 20 nm, a Digital Integration Time of 2 seconds, a data pitch of 1 nm, and averaged over 100 spectra.

Low resolution electrospray ionization mass spectra (LR-ESI-MS) were measured on a Micromass Quattro LC mass spectrometer. High resolution electrospray ionisation mass spectra (HR-ESI-MS) were recorded on a Waters Synapt G2-Si instrument.

Isothermal titration calorimetry (ITC) experiments were performed at 25 °C on a Nano ITC (TA, USA). All ITC titrations were performed in MeCN.

2 Synthesis and Characterization of Subcomponent A

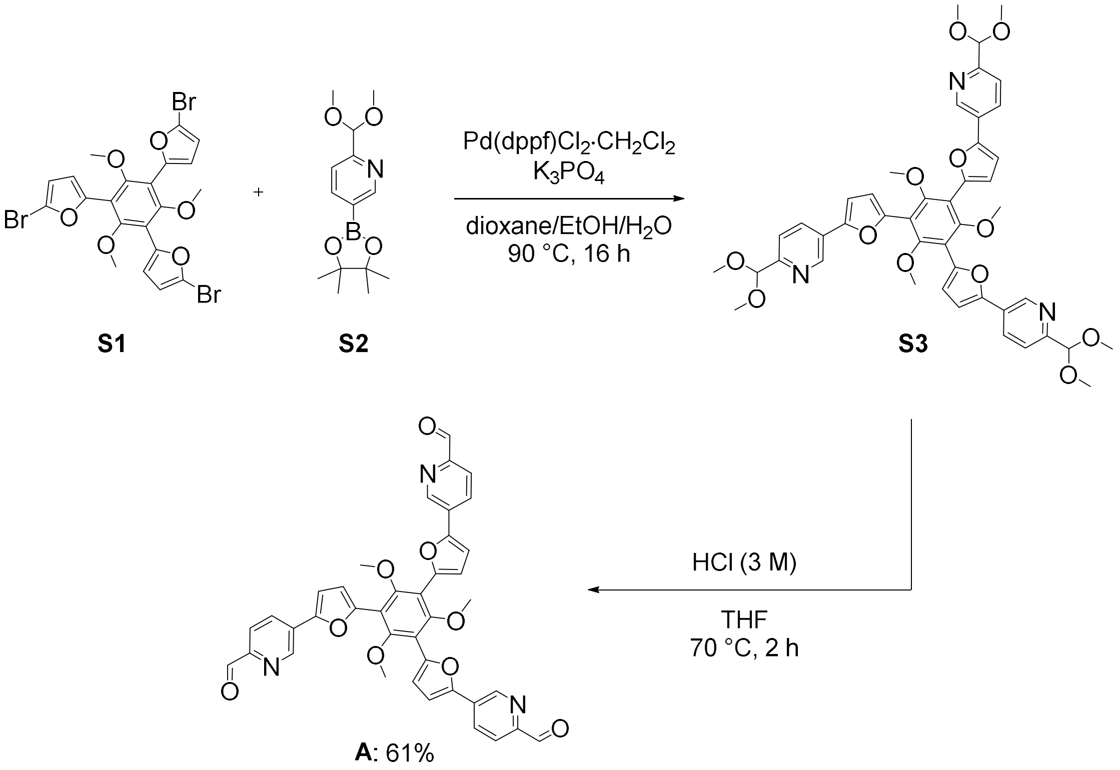

To a solution of 5,5',5''-(2,4,6-trimethoxybenzene-1,3,5-triyl)tris(2-bromofuran) (**S1**, 301.5 mg, 0.5 mmol, 1.0 equiv) and boronic acid pinacol ester (**S2**, 502.5 mg, 1.8 mmol, 3.6 equiv) in a solvent mixture of 1,4-dioxane (14 mL), EtOH (4 mL) and H_2O (2 mL), were added Pd(dppf)Cl_2·CH_2Cl_2 (122.5 mg, 0.15 mmol, 0.3 equiv) and K_3PO_4 (382.1 mg, 1.8 mmol, 3.6 equiv). The reaction mixture was refluxed at 90 °C for 16 hours under nitrogen. After cooling down to room temperature, CH_2Cl_2 (100 mL) was added, and the mixture was then filtered through Celite. The organic phase was washed with brine (100 mL) and H_2O (100 mL), and the combined aqueous phase was extracted with CH_2Cl_2 (2 x 100 mL). The combined organic phases were dried over anhydrous Na_2SO_4, filtered, and the solvents were evaporated under reduced pressure. The crude compound **S3** was obtained as a brown solid and used without purification.

Crude compound **S3** was dissolved in a solvent mixture of THF (30 mL) and HCl (5 mL, 3 M). The reaction solution was then refluxed at 70 °C for 2 hours. After cooling down to room temperature, saturated Na_2CO_3 solution (50 mL) was progressively added, and the mixture was extracted with CH_2Cl_2 (2 x 100 mL). The combined

organic phases were dried over anhydrous Na_2SO_4, filtered, and the solvents were evaporated under reduced pressure. The residual solid was purified by flash column chromatography filled with silica gel, using hexane/EtOAc = 40/60 as eluent, affording subcomponent **A** as a yellow solid (415.8 mg, 61% over two steps).

$\mathbf{R}_f$ = 0.55 (hexane/EtOAc = 40/60).

^{1}H NMR (500 MHz, $CDCl_3$): δ 10.07 (s, 3H), 9.33 (s, 3H), 8.145 (d, *J* = 8.1 Hz, 3H), 8.00 (d, *J* = 8.1 Hz, 3H), 7.13 (d, *J* = 3.5, 3H), 6.92 (d, *J* = 3.5, 3H), 3.59 (s, 9H) ppm.

^{13}C NMR (126 MHz, $CDCl_3$): δ 192.6, 159.0, 151.0, 149.9, 148.2, 145.4, 130.9, 130.2, 122.1, 115.8, 114.4, 111.0, 61.7 ppm.

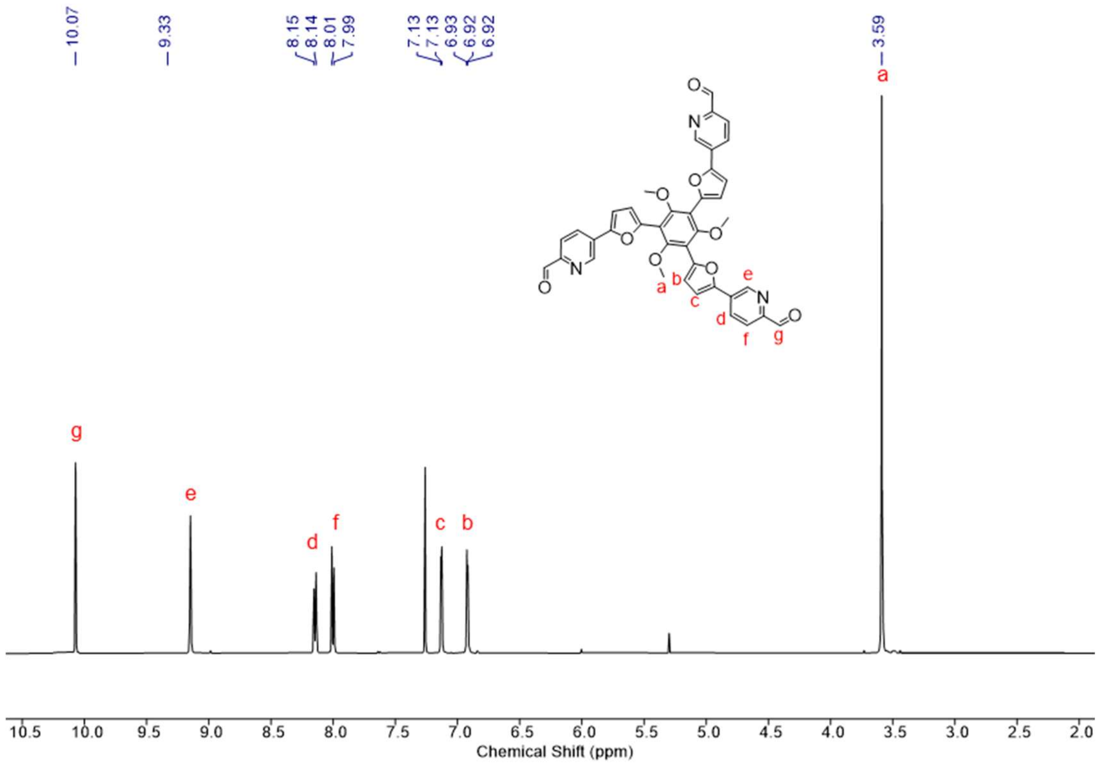

Figure S1. ^{1}H NMR spectrum of subcomponent **A** (500 MHz, $CDCl_3$, 25 °C).

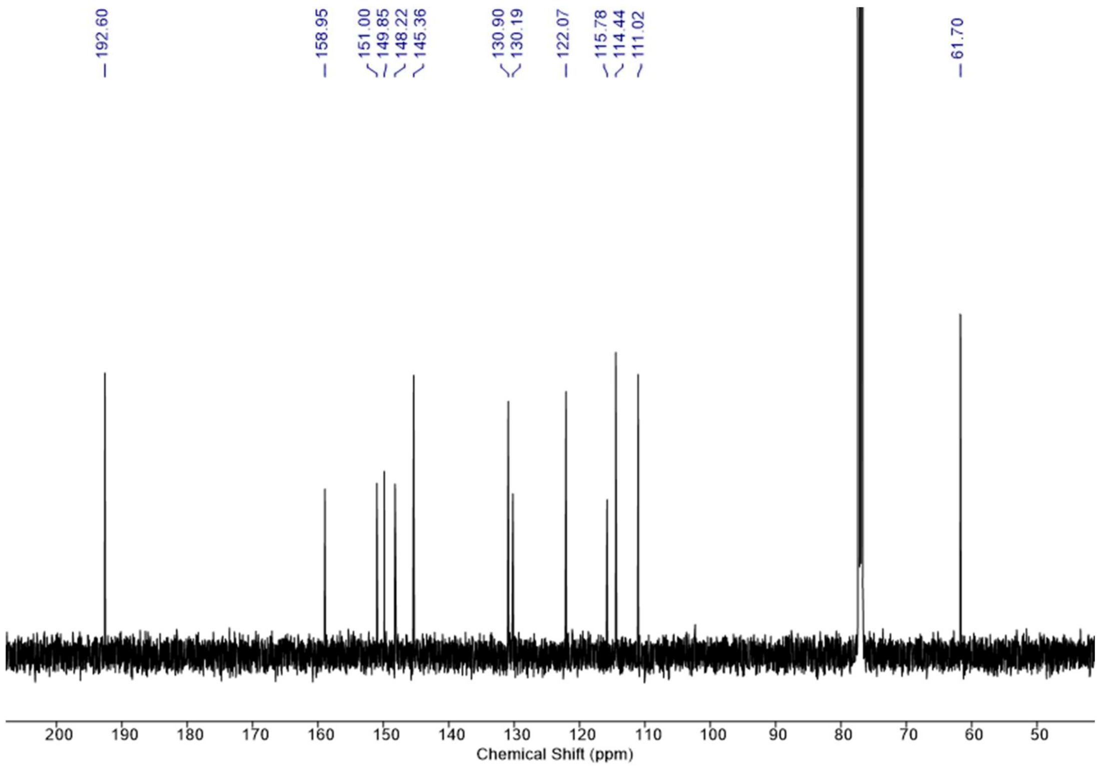

Figure S2. ^{13}C NMR spectrum of subcomponent **A** (126 MHz, $CDCl_3$, 25 °C).

3 Self-Assembly and Characterization of $Zn^{II}_4L_4$ Cages

3.1 Self-Assembly of Enantiopure *Δ*$_4$-1

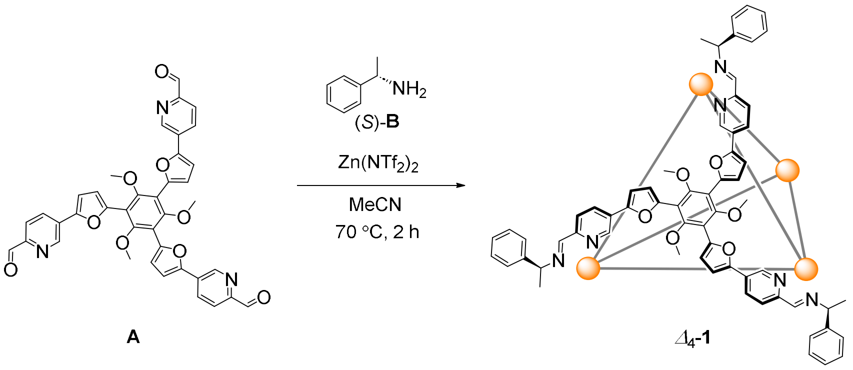

Subcomponent **A** (13.6 mg, 20.0 μmol, 1.0 equiv), (*S*)-1-phenylethylamine ((*S*)-**B**, 7.3 mg, 60.0 μmol, 3.0 equiv) and $Zn(NTf_2)_2$ (12.5 mg, 20.0 μmol, 1.0 equiv) were combined in MeCN (5 mL) in a 25 mL flask. The reaction mixture was stirred at 70 °C for 2 hours. The solvent was reduced to around 1 mL, and Et_2O (15 mL) was then added. The precipitate was collected by centrifugation and washed with excess Et_2O, affording *Δ*$_4$-**1** as a yellow solid (27.5 mg, 85%).

^{1}H NMR (500 MHz, CD_3CN): δ 8.54 (s, 12H), 8.19 (dd, *J* = 1.8, 8.1 Hz, 12H), 7.95 (d, *J* = 1.8 Hz, 12H), 7.49 (d, *J* = 8.1 Hz, 12H), 7.40 (d, *J* = 3.6 Hz, 12H), 7.27 (t, *J* = 7.5 Hz, 12H), 7.04–7.12 (m, 24H), 6.66 (d, *J* = 7.3 Hz, 24H), 6.56 (d, *J* = 3.6 Hz, 12H), 5.26–5.34 (m, 12H), 2.75 (s, 36H), 1.74 (d, *J* = 6.8 Hz, 36H).

^{13}C NMR (126 MHz, CD_3CN): δ 163.3, 157.6, 148.9, 148.8, 145.6, 143.5, 141.4, 137.2, 132.0, 131.2, 130.1, 129.0, 126.4, 120.9 (q, *J* = 320 Hz, CF_3 from NTf_2), 116.0, 115.24, 115.19, 64.8, 61.7, 24.3 ppm.

HR-ESI-MS: m/z (found) = 527.1558 [**1**-8(NTf_2)]$^{8+}$, 797.5160 [**1**-6(NTf_2)]$^{6+}$, 1013.1936 [**1**-5(NTf_2)]$^{5+}$, 1336.2240 [**1**-4(NTf_2)]$^{4+}$, 1875.2620 [**1**-3(NTf_2)]$^{3+}$.

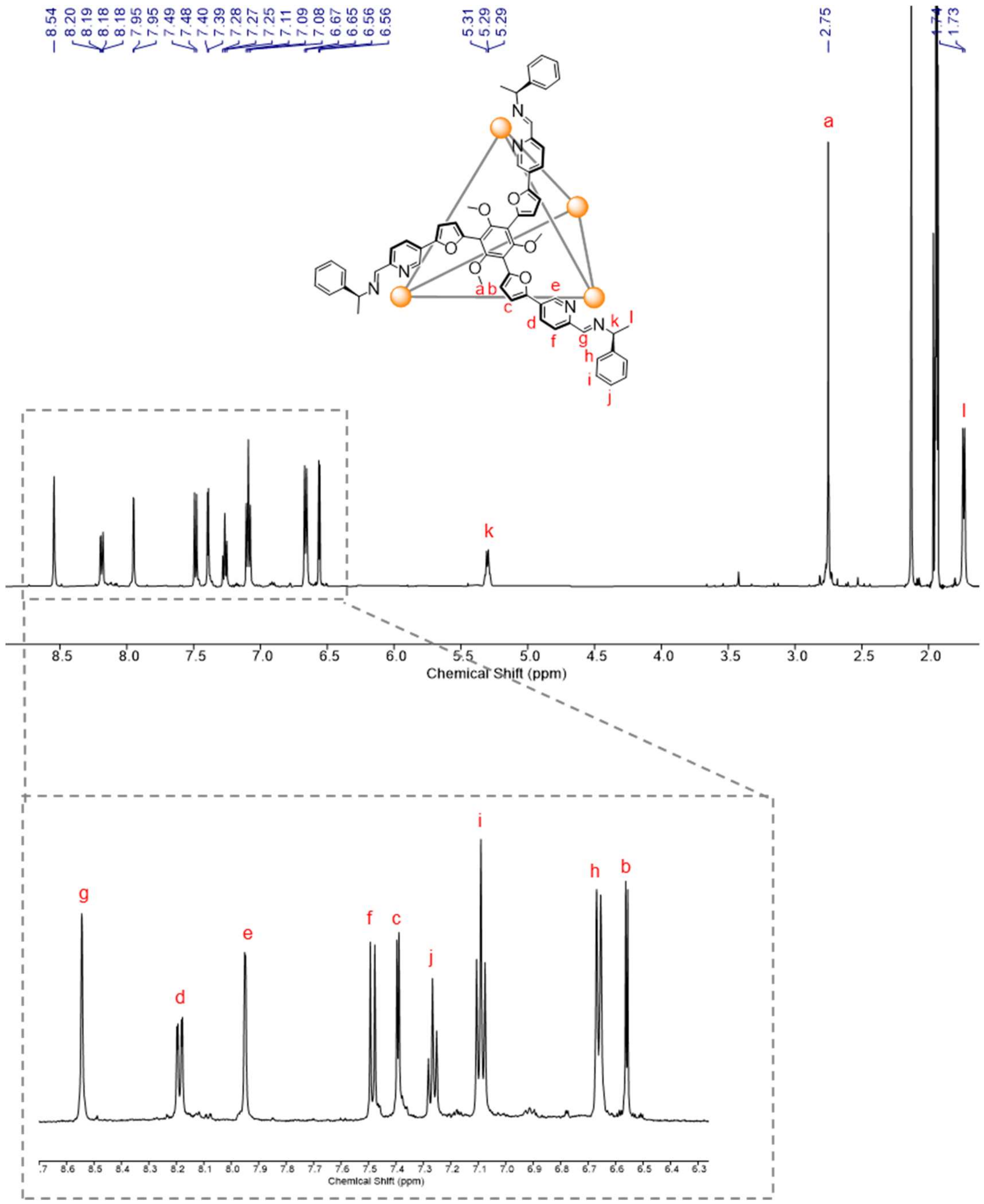

Figure S3. ^{1}H NMR spectrum of Δ_4-**1** (500 MHz, CD_3CN, 25 °C).

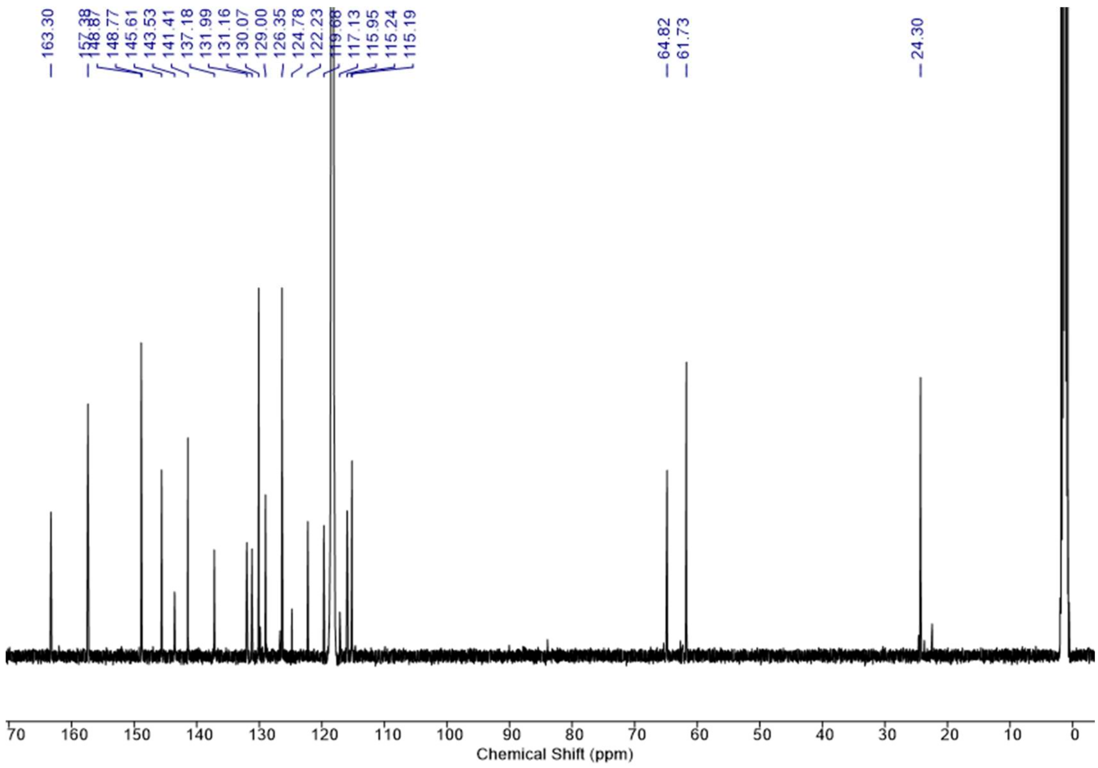

Figure S4. ^{13}C NMR spectrum of Δ_4-**1** (126 MHz, CD_3CN, 25 °C).

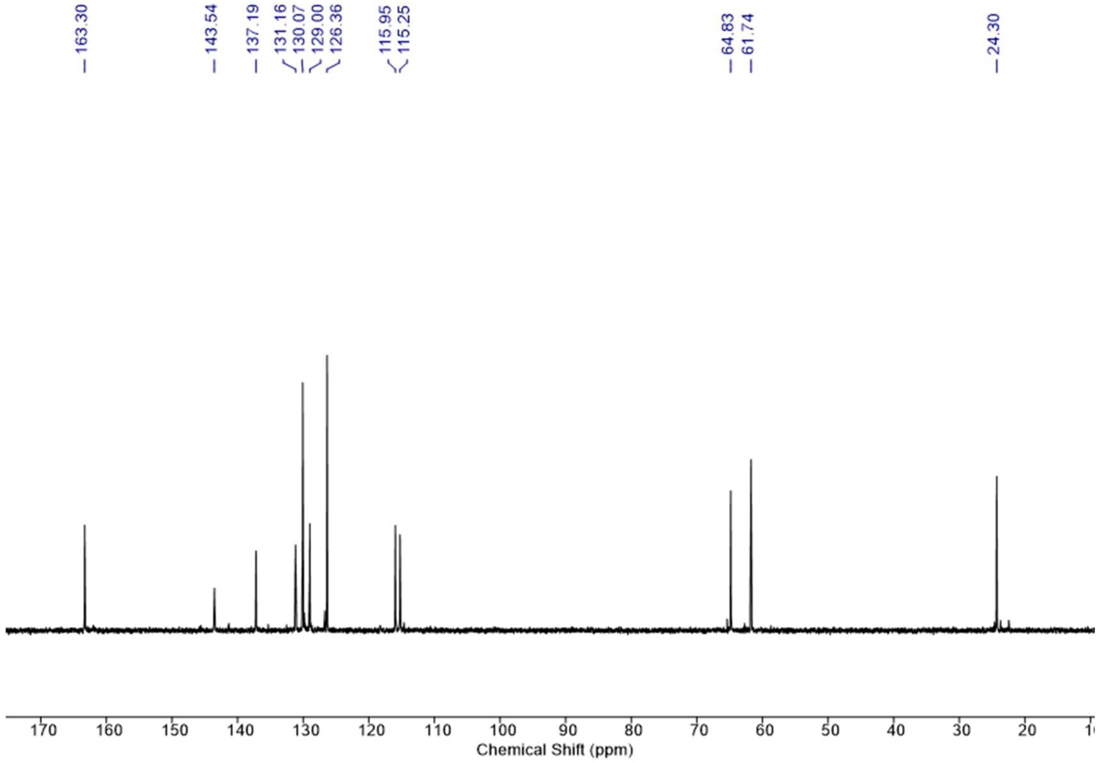

Figure S5. ^{13}C DEPT-135 NMR spectrum of Δ_4-**1** (126 MHz, CD_3CN, 25 °C).

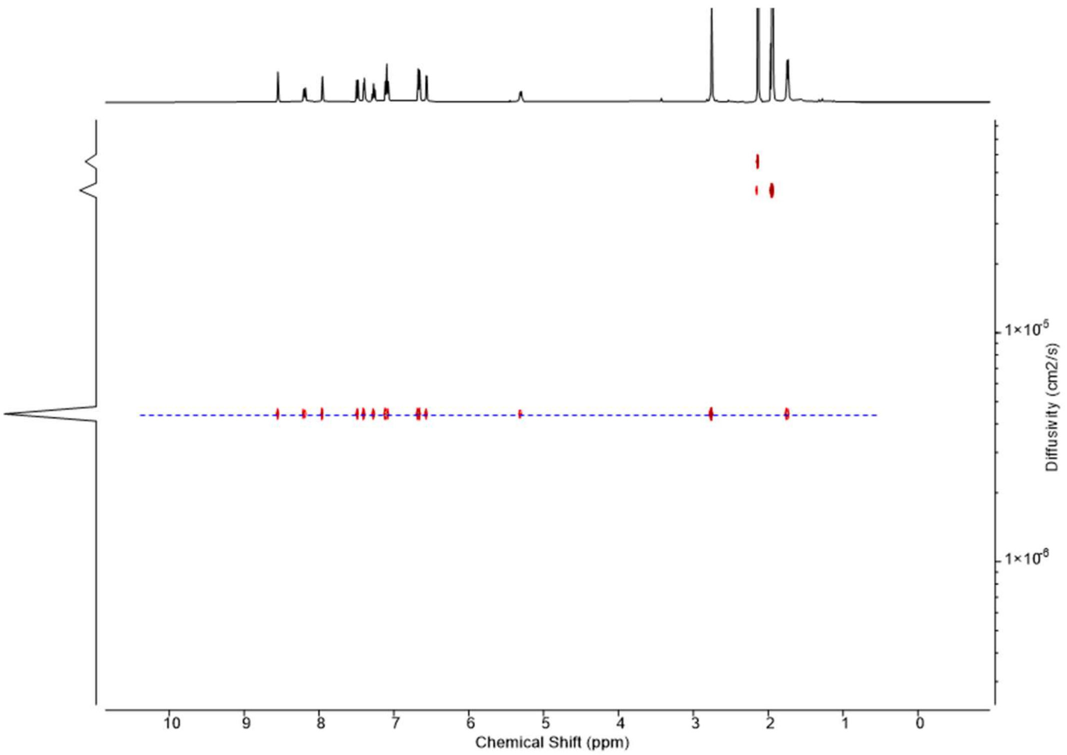

Figure S6. ^{1}H DOSY spectrum of *Δ*$_4$-**1** (400 MHz, CD_3CN, 25 °C). The diffusion coefficient was measured to be 4.52×10^{-6} cm^2/s.

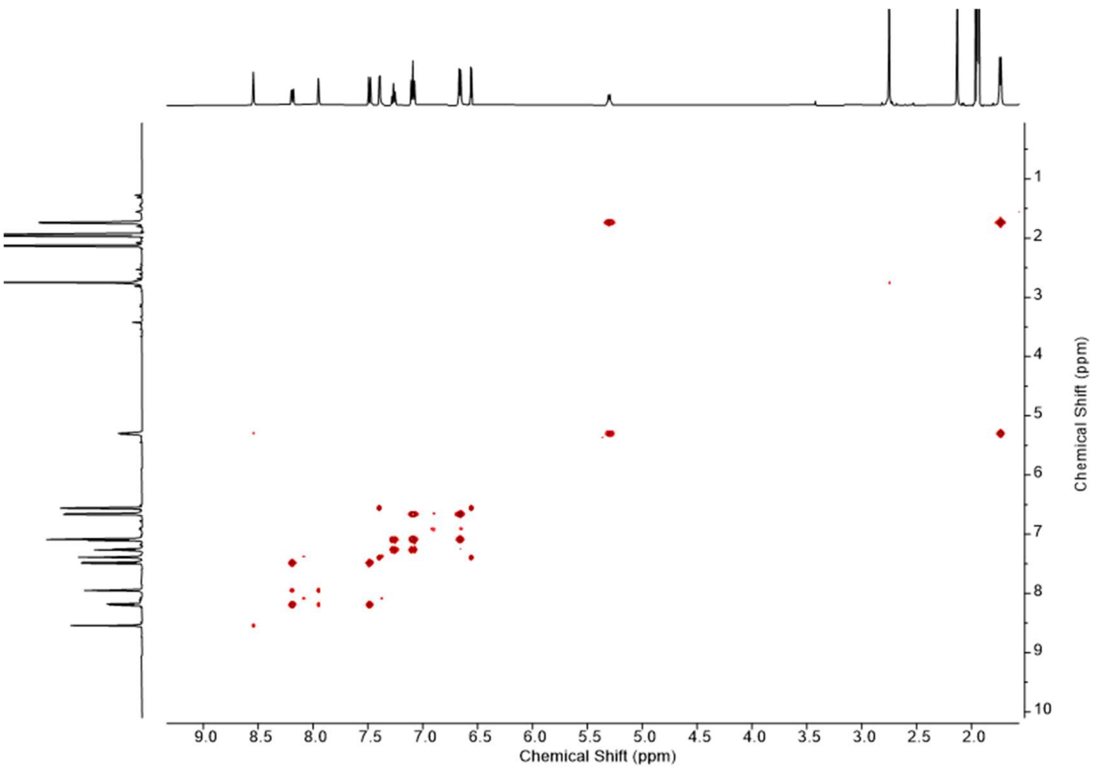

Figure S7. ^{1}H-^{1}H COSY NMR spectrum of *Δ*$_4$-**1** (500 MHz, CD_3CN, 25 °C).

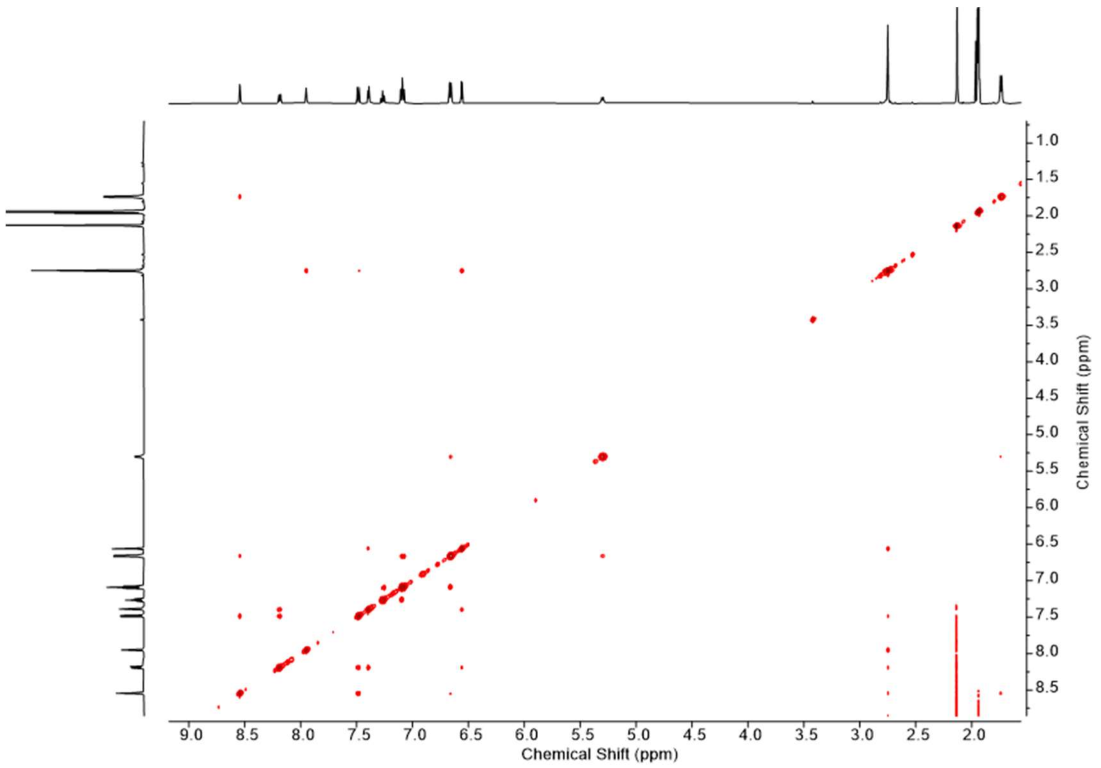

Figure S8. ^{1}H-^{1}H NOESY NMR spectrum of Δ_4-**1** (500 MHz, CD_3CN, 25 °C).

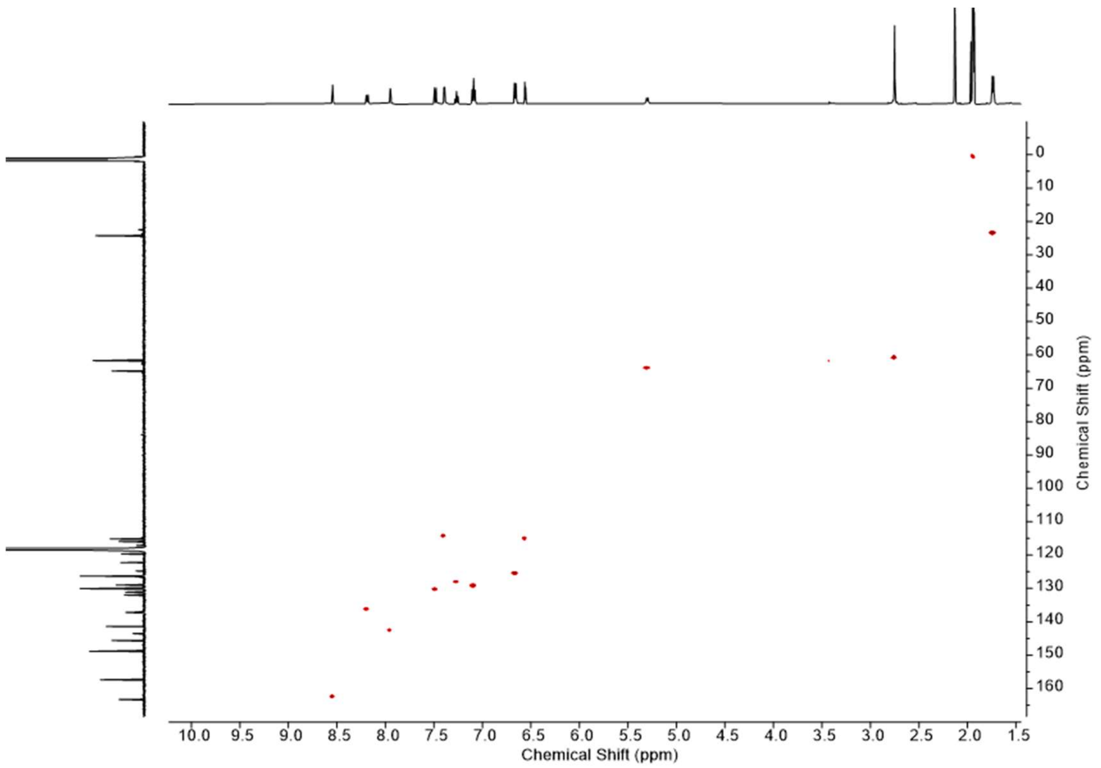

Figure S9. ^{1}H-^{13}C HSQC NMR spectrum of Δ_4-**1** (500 MHz, CD_3CN, 25 °C).

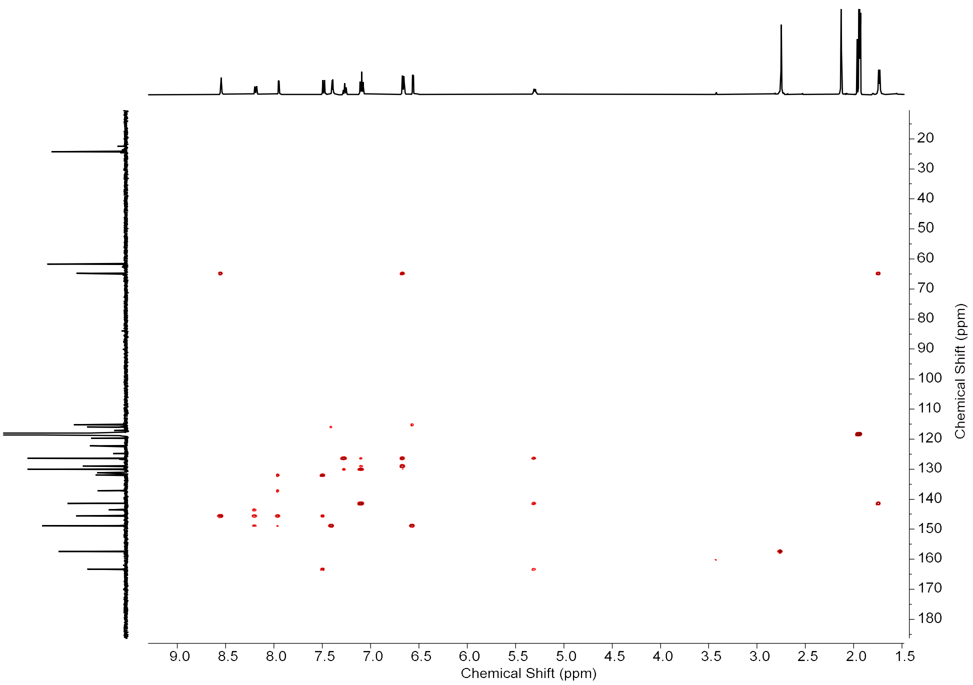

Figure S10. ^{1}H-^{13}C HMBC NMR spectrum of *Δ*$_4$-**1** (500 MHz, CD_3CN, 25 °C).

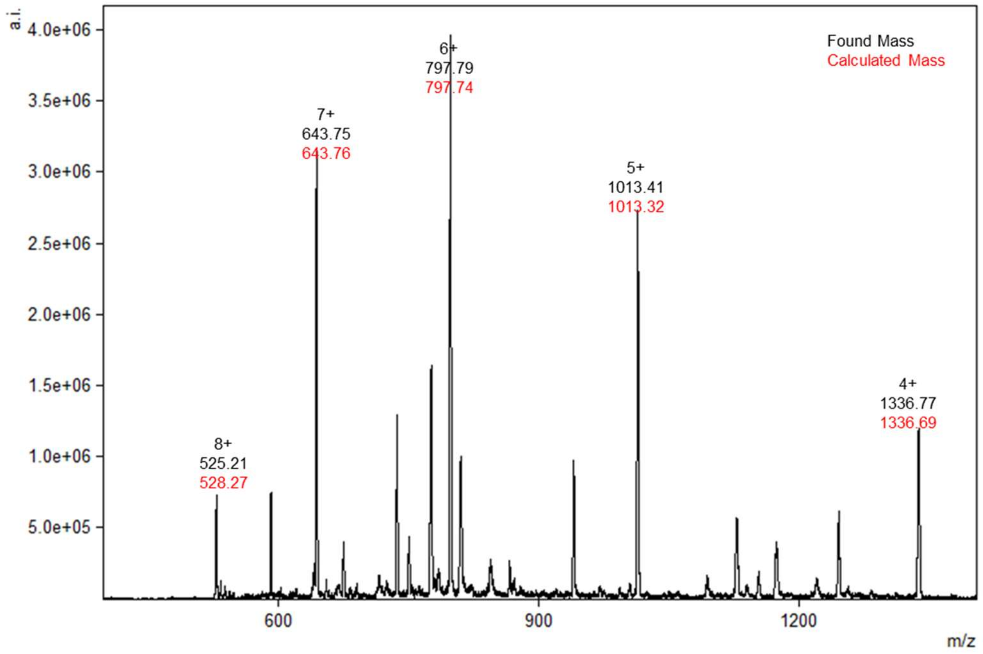

Figure S11. LR-ESI-MS spectrum of *Δ*$_4$-**1** in MeCN.

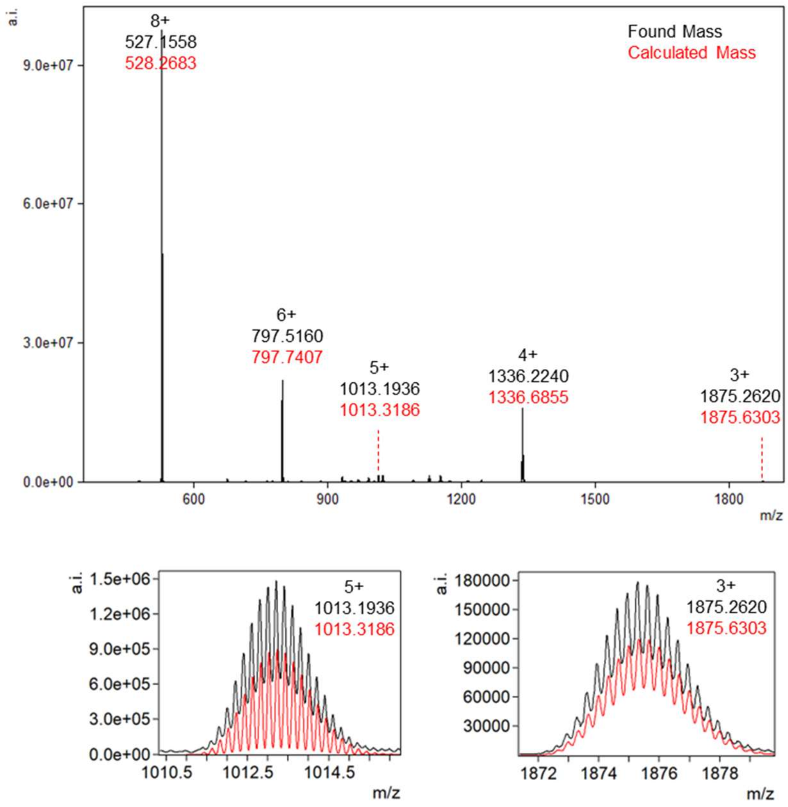

Figure S12. HR-ESI-MS spectrum of *Δ*$_4$-**1** in MeCN. The isotope distribution for the 8+, 6+, and 4+ peaks do not perfectly match due to cleavage of the Zn_4L_4 framework under test conditions.

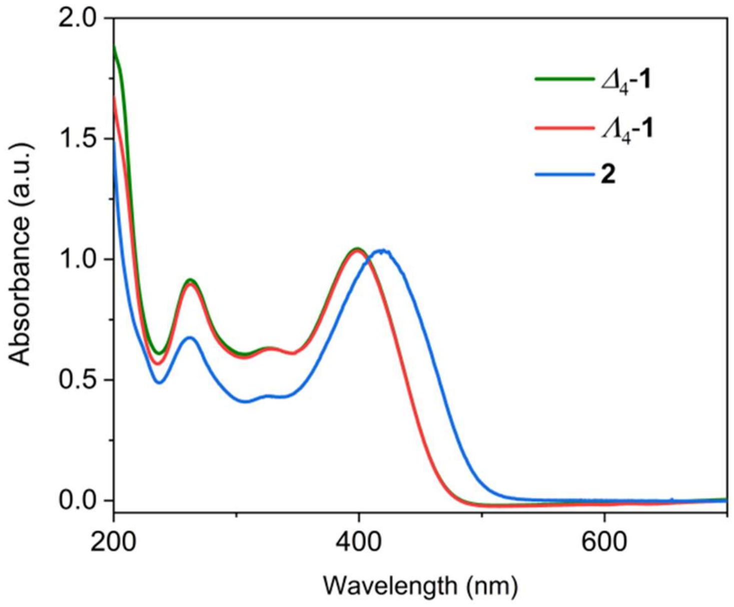

Figure S13. UV-vis spectra of the $Zn^{II}_4L_4$ cages in MeCN (50 μM of cage).

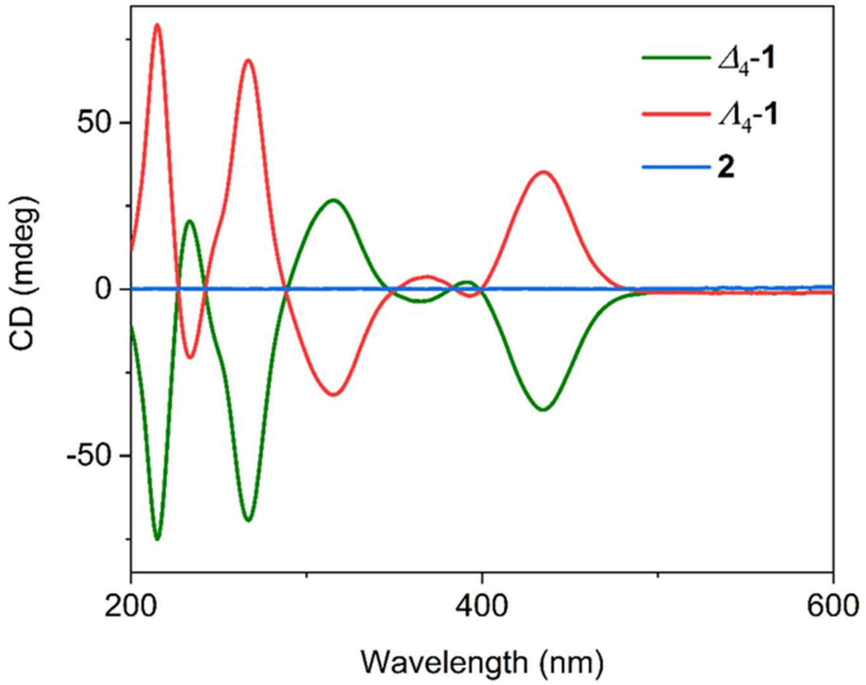

Figure S14. CD spectra of the $Zn^{II}_4L_4$ cages in MeCN (50 μM of cage).

3.2 Self-Assembly of Enantiopure *Λ*$_4$-1

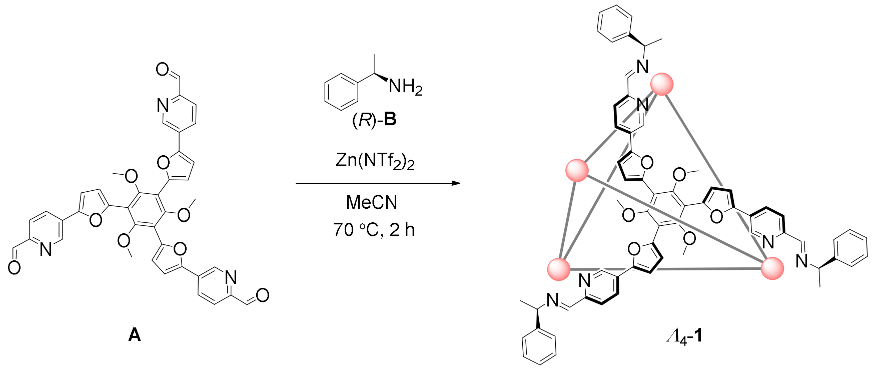

Subcomponent **A** (13.6 mg, 20.0 μmol, 1.0 equiv), (*R*)-1-phenylethylamine ((*R*)-**B**, 7.3 mg, 60.0 μmol, 3.0 equiv) and $Zn(NTf_2)_2$ (12.5 mg, 20.0 μmol, 1.0 equiv) were combined in MeCN (5 mL) in a 25 mL flask. The reaction mixture was stirred at 70 °C for 2 hours. The solvent was reduced to around 1 mL, and Et_2O (15 mL) was then added. The precipitate was collected by centrifugation and washed with excess Et_2O, affording *Λ*$_4$-**1** as a yellow solid (28.1 mg, 87%).

The identical ^{1}H NMR spectra of *Δ*$_4$-**1** and *Λ*$_4$-**1** prepared from (*S*)-**B** and (*R*)-**B**, respectively, indicated that *Δ*$_4$-**1** and *Λ*$_4$-**1** are a pair of enantiomers, consistent with the circular dichroism (CD) spectra showing mirror-image bisignate CD curves (Figure S14). The same diffusion coefficient was observed for *Δ*$_4$-**1** and *Λ*$_4$-**1**.

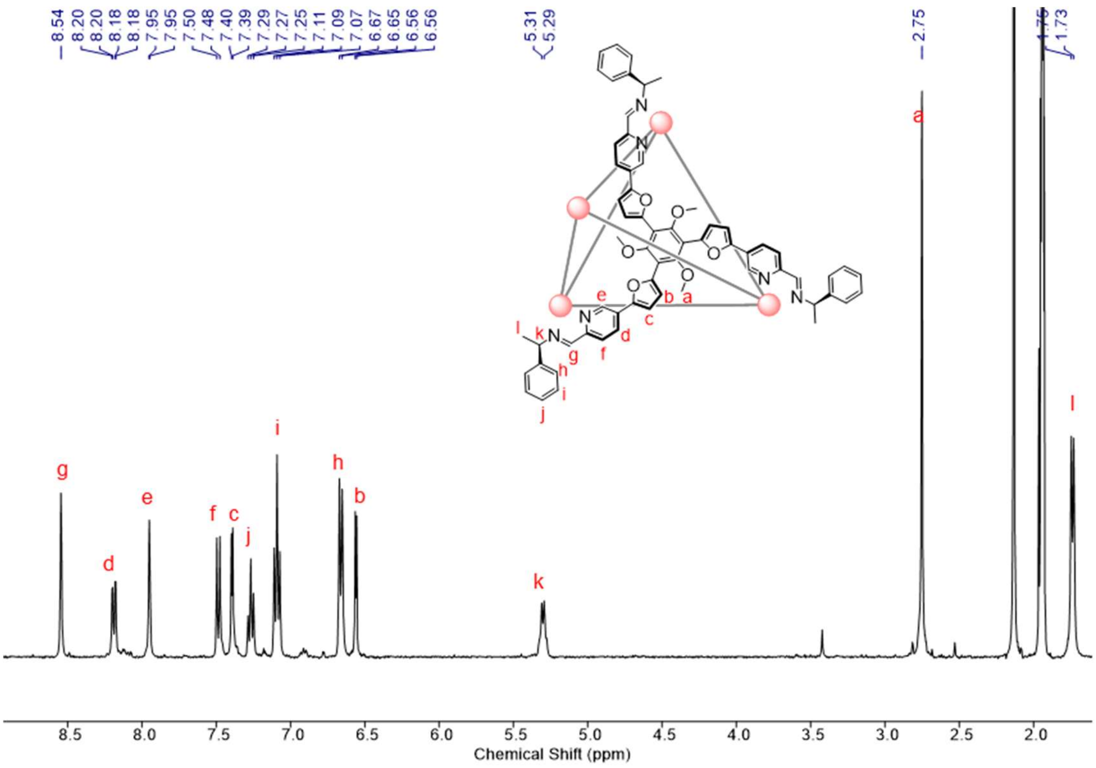

Figure S15. ^{1}H NMR spectrum of Λ_4-**1** (400 MHz, CD_3CN, 25 °C).

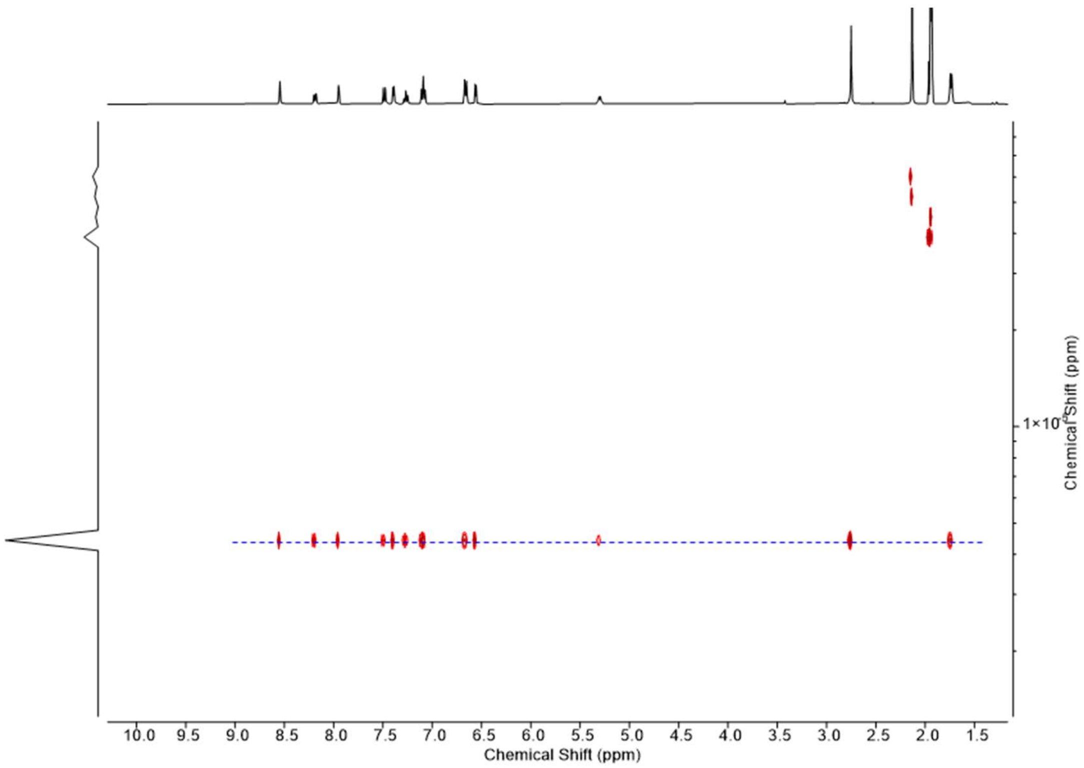

Figure S16. ^{1}H DOSY spectrum of Λ_4-**1** (400 MHz, CD_3CN, 25 °C). The diffusion coefficient was measured to be 4.52×10^{-6} cm^2/s.

3.3 Self-Assembly of Racemic 2

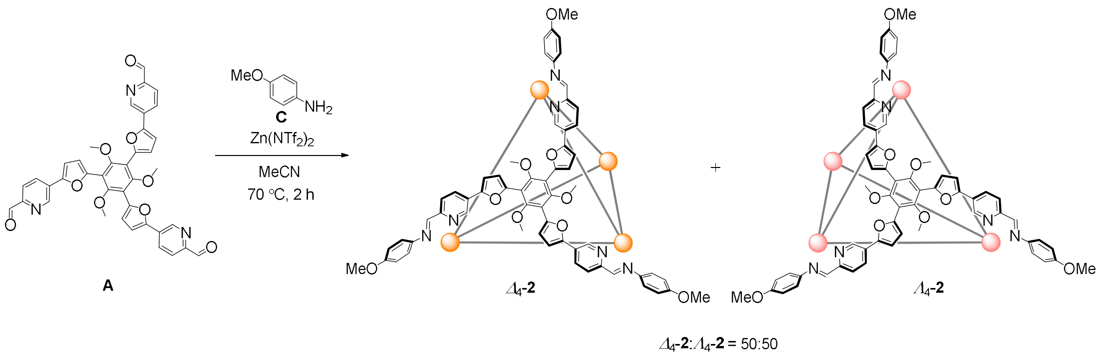

Subcomponent **A** (13.6 mg, 20.0 µmol, 1.0 equiv), 4-methoxyaniline (**C**, 7.4 mg, 60.0 µmol, 3.0 equiv) and $Zn(NTf_2)_2$ (12.5 mg, 20.0 µmol, 1.0 equiv) were combined in MeCN (5 mL) in a 25 mL flask. The reaction mixture was stirred at 70 °C for 2 hours. The solvent was reduced to around 1 mL, and Et_2O (15 mL) was then added. The precipitate was collected by centrifugation and washed with excess Et_2O, affording **2** as a brown solid (29.2 mg, 90%).

Cage **2** exists as a racemic mixture, consisting of $\mathit{\Delta}_4$-**2** and $\mathit{\Lambda}_4$-**2** with a ratio of 50:50, consistent with the absence of CD signals as shown in Figure S13.

Note: The four ligand faces adopt an anticlockwise orientation to pair with *Δ* handedness, whereas a clockwise orientation of ligands was observed for *Λ* handedness, as observed in the crystal structures of **1** and **2** (Figure S52). However, in some batches, an additional pair of enantiomers with anticlockwise-*Λ* and clockwise-*Δ* configurations may also form during the self-assembly process. This can result in minor impurities corresponding to diastereomers of compound **2** appearing in the NMR spectra.

^{1}H NMR (500 MHz, CD_3CN): δ 8.63 (dd, *J* = 1.9, 8.2 Hz, 12H), 8.47 (s, 12H), 8.46 (d, *J* = 1.9 Hz, 12H), 8.22 (d, *J* = 8.2 Hz, 12H), 7.53 (d, *J* = 3.7 Hz, 12H), 6.86 (d, *J* = 7.5 Hz, 24H), 6.77 (d, *J* = 3.7 Hz, 12H), 6.39 (d, *J* = 3.7 Hz, 24H), 3.81 (s, 36H), 2.99 (d, *J* = 6.8 Hz, 36H).

^{13}C NMR (176 MHz, CD_3CN): δ 162.4, 161.0, 157.8, 149.2, 149.1, 146.2, 144.9, 140.6, 137.2, 132.9, 130.9, 120.9 (q, J = 320 Hz, CF_3 from NTf_2), 116.4, 115.70, 115.65, 115.5 62.2, 56.4 ppm.

HR-ESI-MS: m/z (found) = 530.1409 [**2**-8(NTf_2)]$^{8+}$, 801.4932 [**1**-6(NTf_2)]$^{6+}$, 1017.9742 [**2**-5(NTf_2)]$^{5+}$, 1342.1995 [**2**-4(NTf_2)]$^{4+}$, 1883.2390 [**2**-3(NTf_2)]$^{3+}$.

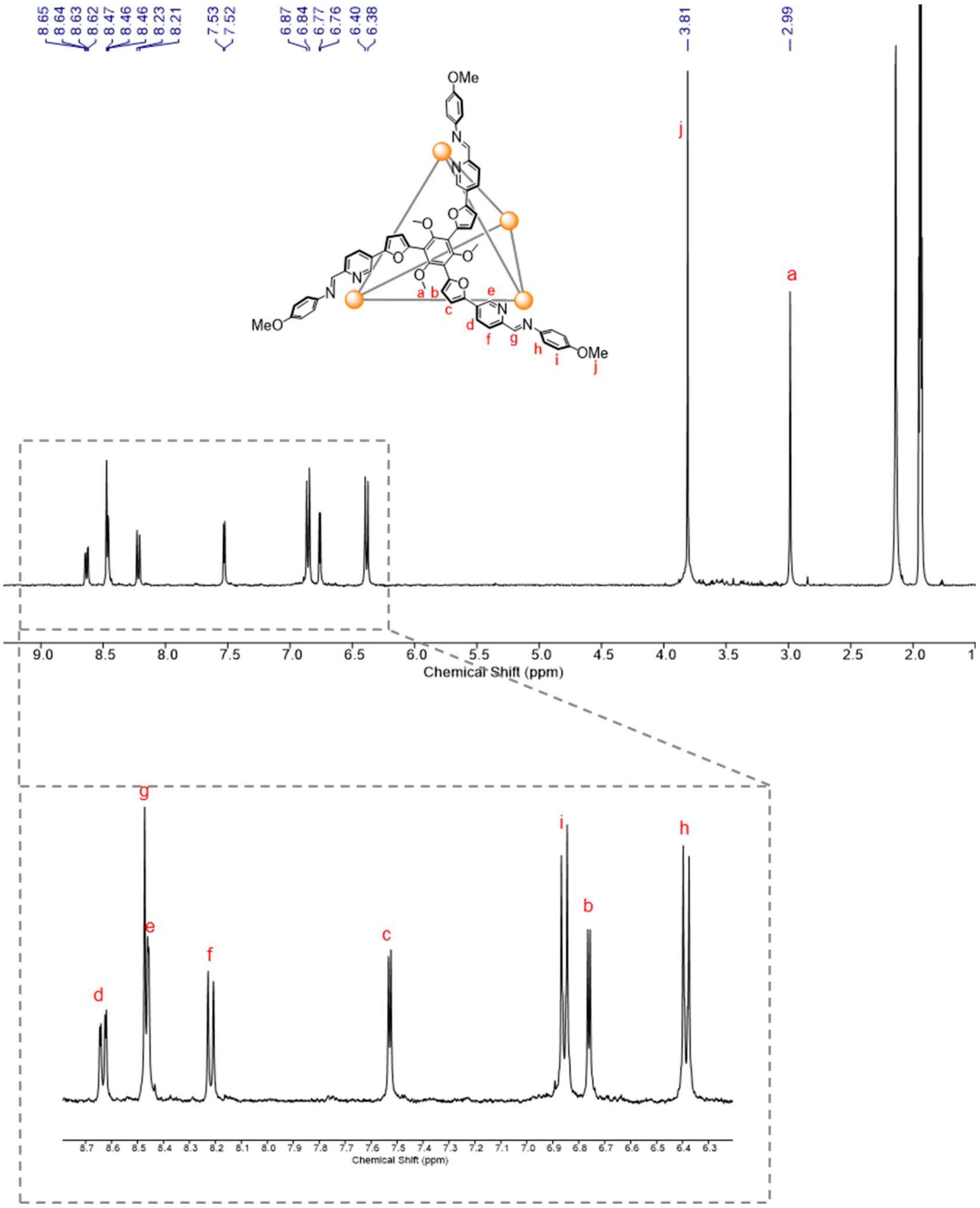

Figure S17. ^{1}H NMR spectrum of **2** (500 MHz, CD_3CN, 25 °C).

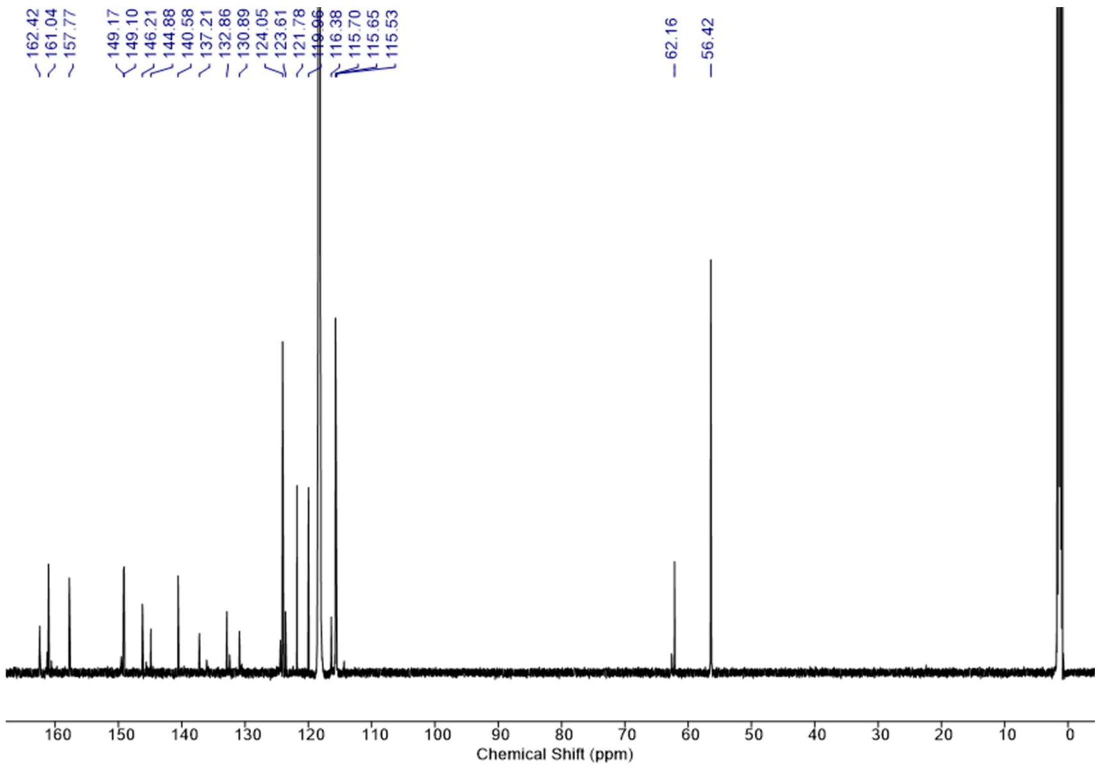

Figure S18. ^{13}C NMR spectrum of **2** (176 MHz, CD_3CN, 25 °C).

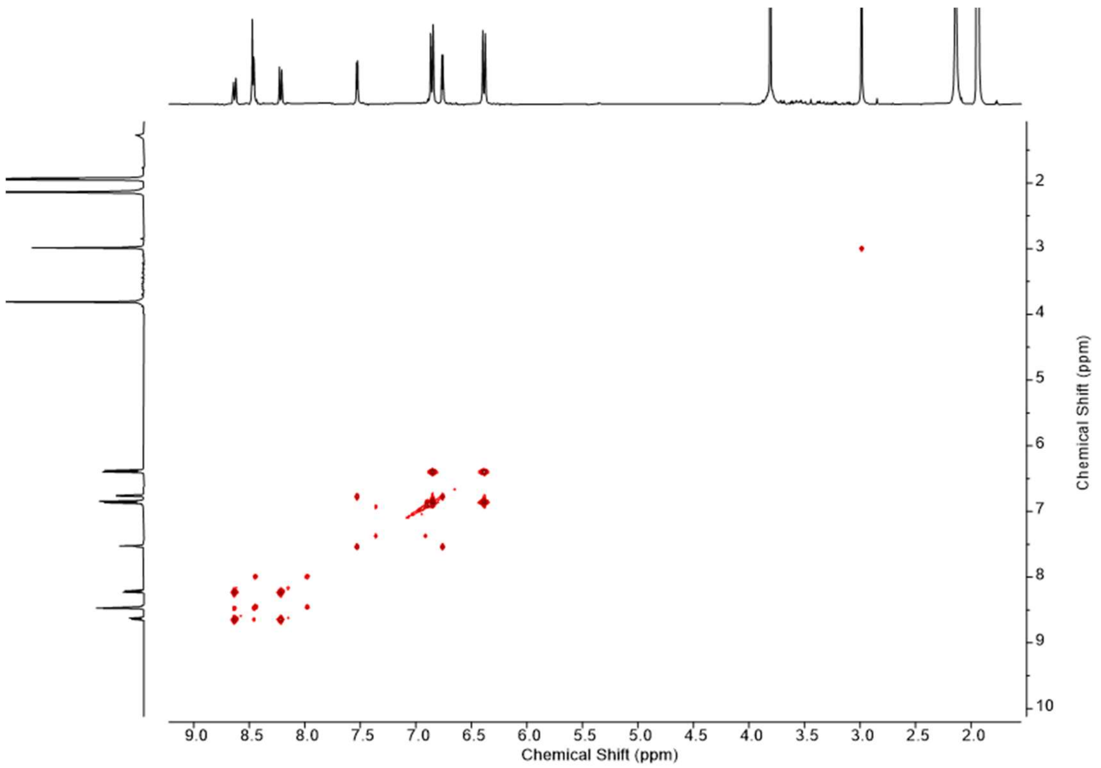

Figure S19. ^{1}H-^{1}H COSY NMR spectrum of **2** (700 MHz, CD_3CN, 25 °C).

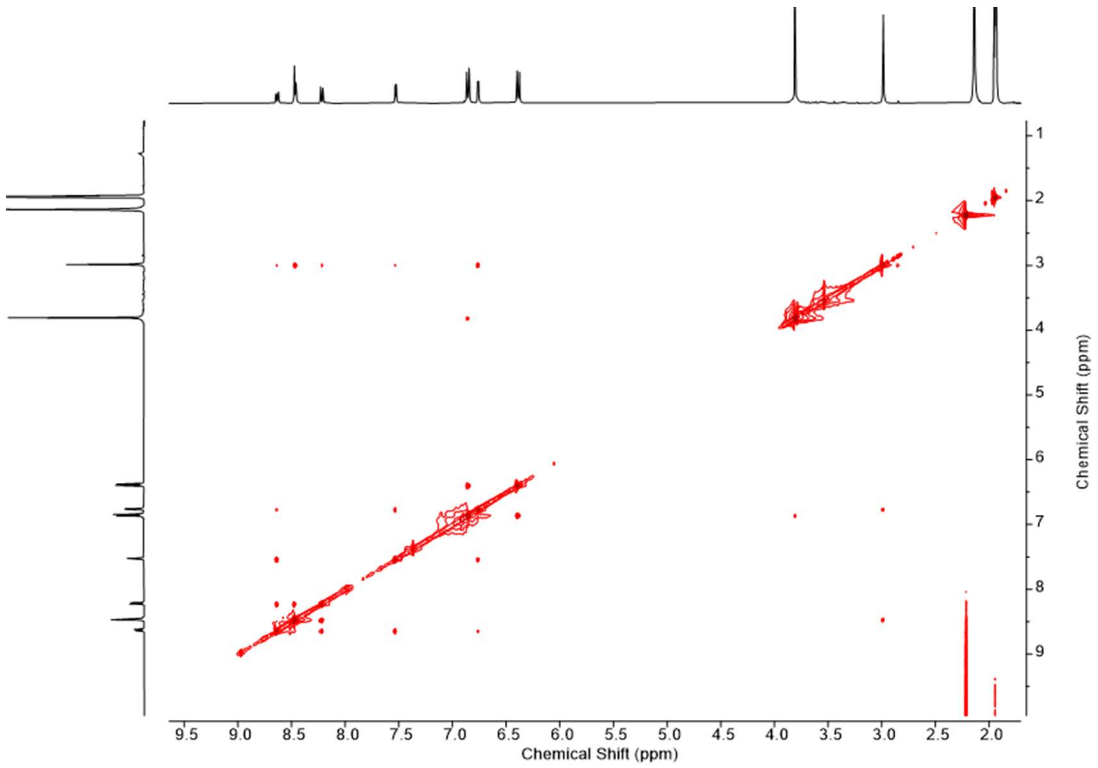

Figure S20. ^{1}H-^{1}H NOESY NMR spectrum of **2** (700 MHz, CD_3CN, 25 °C).

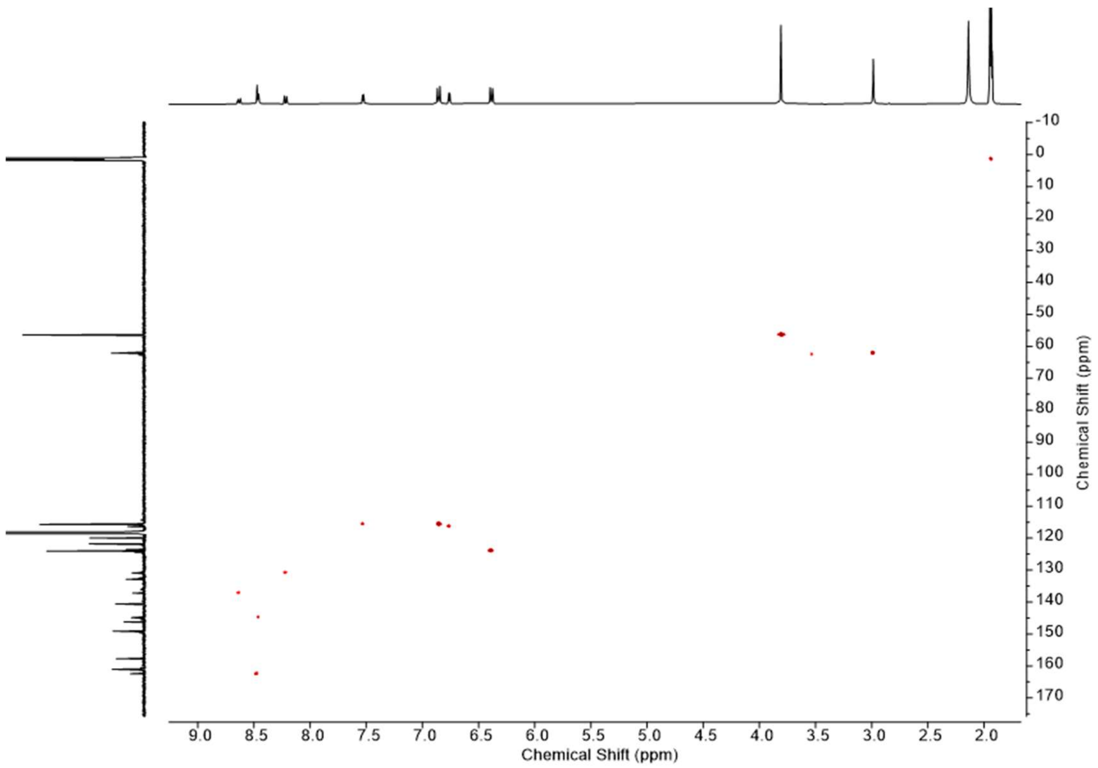

Figure S21. ^{1}H-^{13}C HSQC NMR spectrum of **2** (700 MHz, CD_3CN, 25 °C).

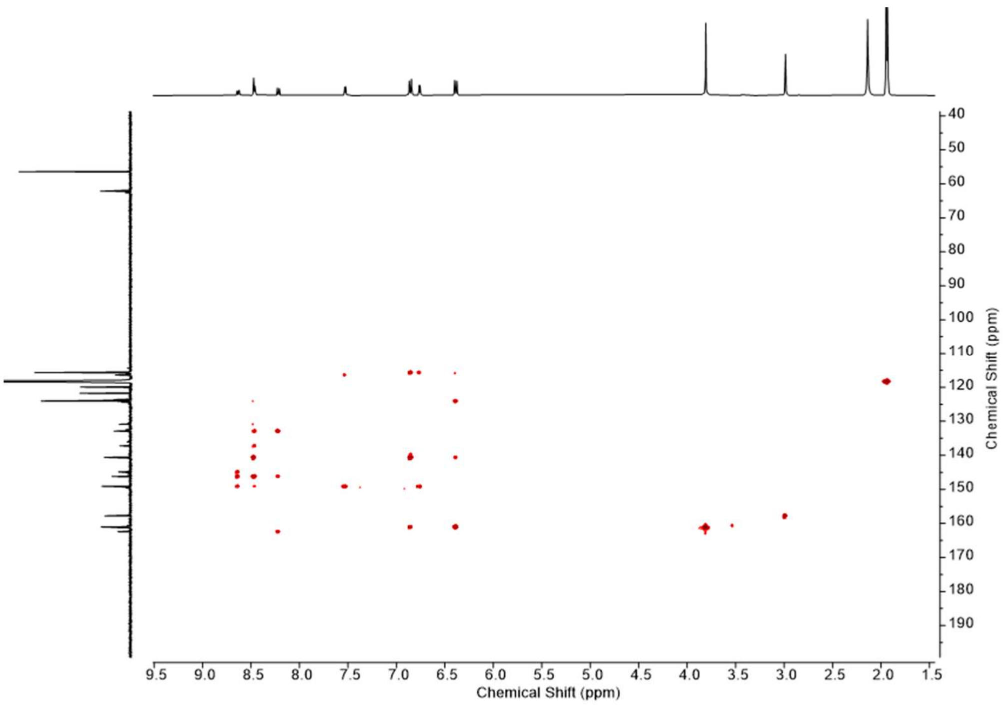

Figure S22. ^1H-^{13}C HMBC NMR spectrum of **2** (700 MHz, CD_3CN, 25 °C).

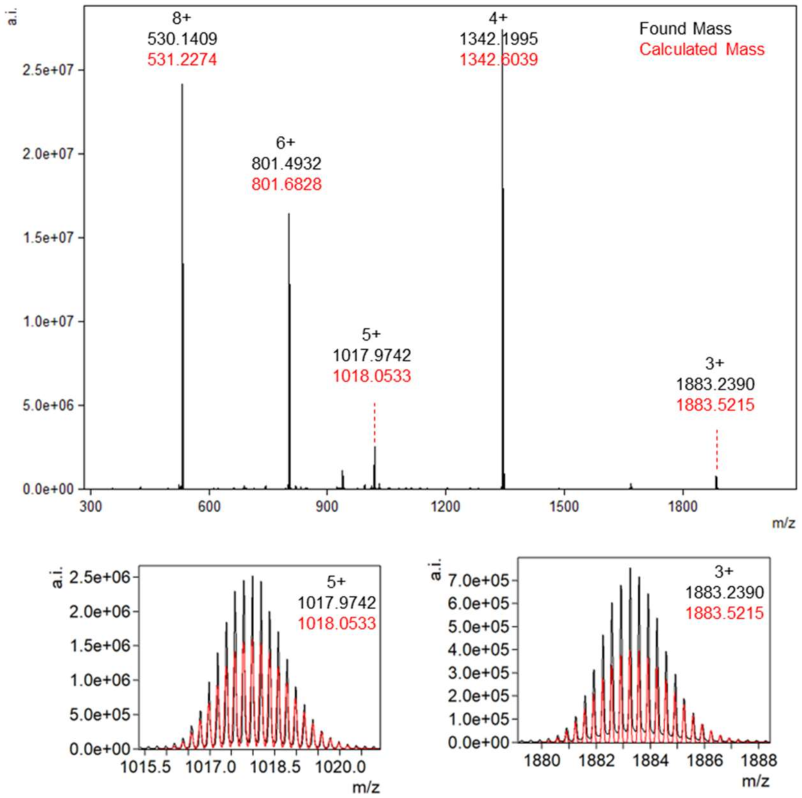

Figure S23. HR-ESI-MS spectrum of **2** in MeCN. The isotope distribution for the 8+, 6+, and 4+ peaks do not perfectly match due to cleavage of the Zn_4L_4 framework under test conditions.

4 Host-Guest Properties of $Zn^{II}_4L_4$ Cages

Host-guest complexes were prepared on an NMR scale in CD_3CN (0.5 mL). The NMR spectra were measured upon heating the mixtures at 70 °C for 30 mins to 2 hours. Binding affinities were quantified by ^1H NMR titrations, and binding stoichiometries were determined by either NMR or ESI-MS. The absence of proton signal shifts of cages indicates that the guests were not bound by the cages.

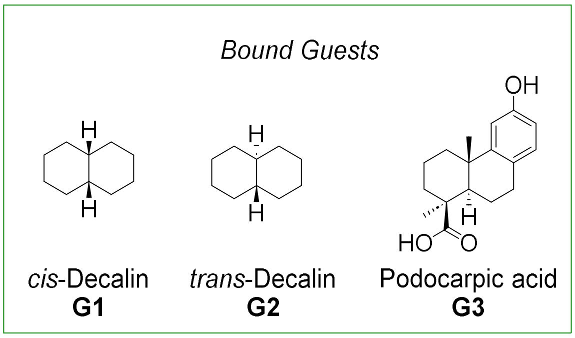

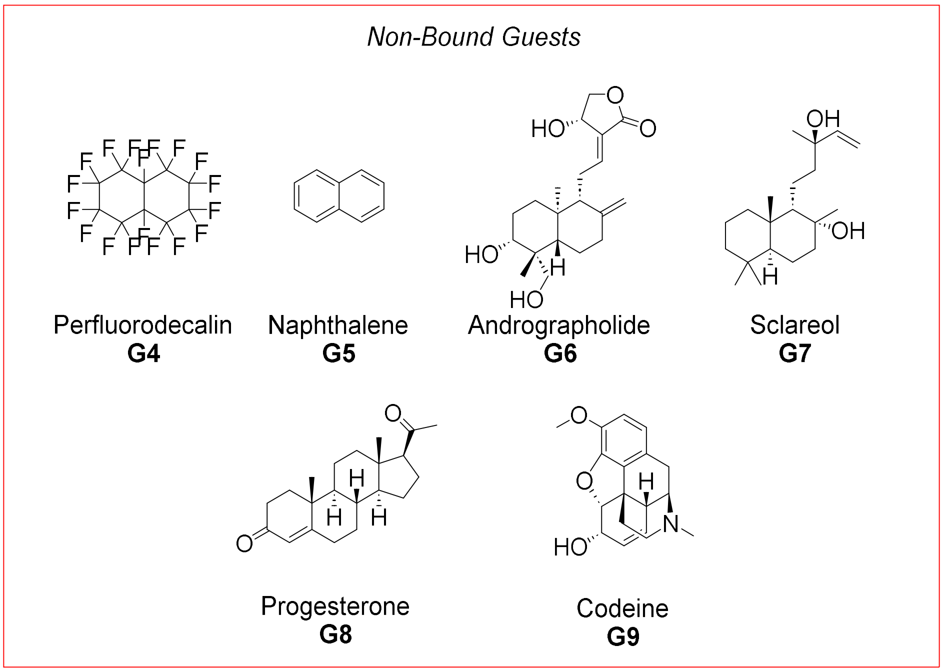

^1H NMR titrations were conducted by the addition a CD_3CN solution of guests or portionwise addition of guests as solid into a stock solution of host in CD_3CN (0.75 mM), using 1,3,5-trimethoxybenzene (1.5 mM) or 1.3.5-trimethylbenzene (1.5 mM) as an internal standard. After each addition, the reaction mixture was heated at 70 °C for 30 minutes. ^1H NMR data was collected after cooling down the host-guest complex.

The binding constant was determined using the following equation:

$$K_a = \frac{[HG]}{[H]([G0]-[HG])}$$

where [HG], [H] are the host-guest complex and host concentration, respectively, and [G0] is the concentration of the guest after it was added into the host solution.

An average value of K_a from each addition of guest was calculated to quantify the binding affinity.

Table S1. Summary of binding constants determined by NMR titrations.[a]

Guest	Host	Binding Constant (M^{-1})
**G1**	Δ_4-**1**	$(1.08 \pm 0.07) \times 10^2$
**G1**	Λ_4-**1**	$(1.10 \pm 0.07) \times 10^2$
**G1**	**2**	$(0.40 \pm 0.10) \times 10^2$
**G2**	Δ_4-**1**	$(0.48 \pm 0.04) \times 10^2$
**G2**	Λ_4-**1**	$(0.47 \pm 0.04) \times 10^2$
**G2**	**2**	$(0.35 \pm 0.10) \times 10^2$
**G3**	Δ_4-**1**	$(1.98 \pm 0.08) \times 10^2$
**G3**	Λ_4-**1**	$(1.44 \pm 0.08) \times 10^2$
**G3**	**2**	—
**G3** carboxylate	Δ_4-**1**	$(2.87 \pm 0.10) \times 10^3$
**G3** carboxylate	Λ_4-**1**	$(2.13 \pm 0.10) \times 10^3$
**G3** carboxylate	**2**	$(1.75 \pm 0.10) \times 10^3$

[a]The data was also summarized in the manuscript Table 1. [b]**G3** carboxylate was prepared by mixing equimolar amounts of **G3** and *N*,*N*-diisopropylethylamine (DIPEA) in CD_3CN.

4.1 Host-Guest Interaction of Enantiopure 1 with G1 and G2

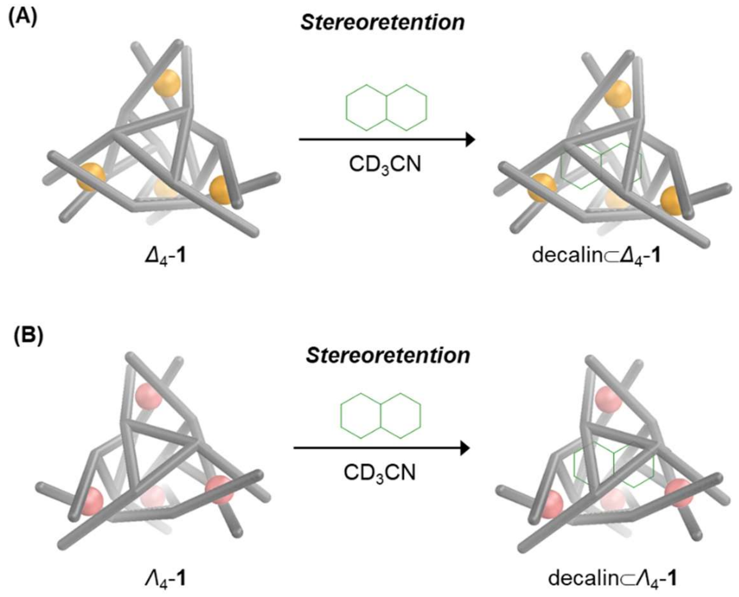

The binding of decalin by **1** was observed to be in slow exchange on the NMR timescale, as proton signals corresponding to host-guest complexes emerged as free **1** disappeared from the ^{1}H NMR spectrum during titration experiments. ^{1}H NMR integration revealed the formation of a 1 :1 host-guest complex. All proton signals from the bound guest shifted significantly upfield as a result of shielding effects, consistent with central binding of decalin.

Both Δ_4-**1** and Λ_4-**1** showed identical binding behavior towards *cis*-decalin **G1** and *trans*-decalin **G2**. The binding affinity of **G1** is higher than that of **G2**. The binding of decalin proceeded in a stereoretentive manner, as observed in CD spectra of the host-guest complexes (Figure S31).

Δ_4-**1** did not exhibit discrimination effects towards **G1** even at −30 °C (Figures S25). NOE correlations between proton signals of bound **G1** indicated that those signals correspond to the same guest molecule (Figure S26).

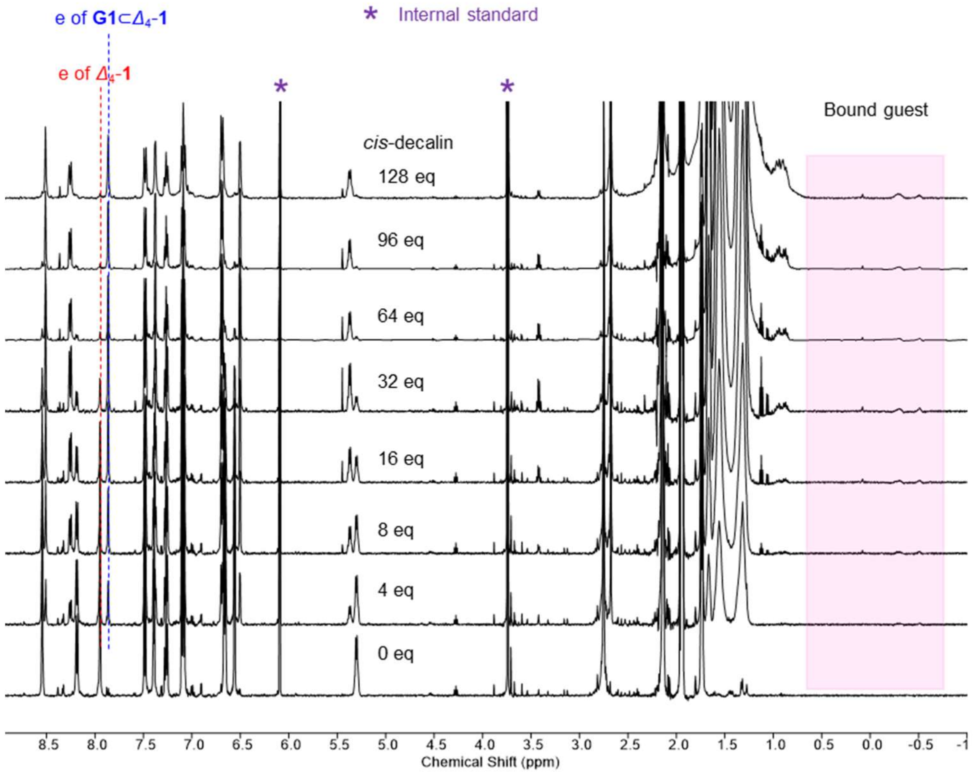

Figure S24. ^{1}H NMR spectra upon addition of **G1** (0.75 M in CD_3CN) into *Δ*$_4$-**1** using 1,3,5-trimethoxybenzene as internal standard (500 MHz, CD_3CN, 25 °C).

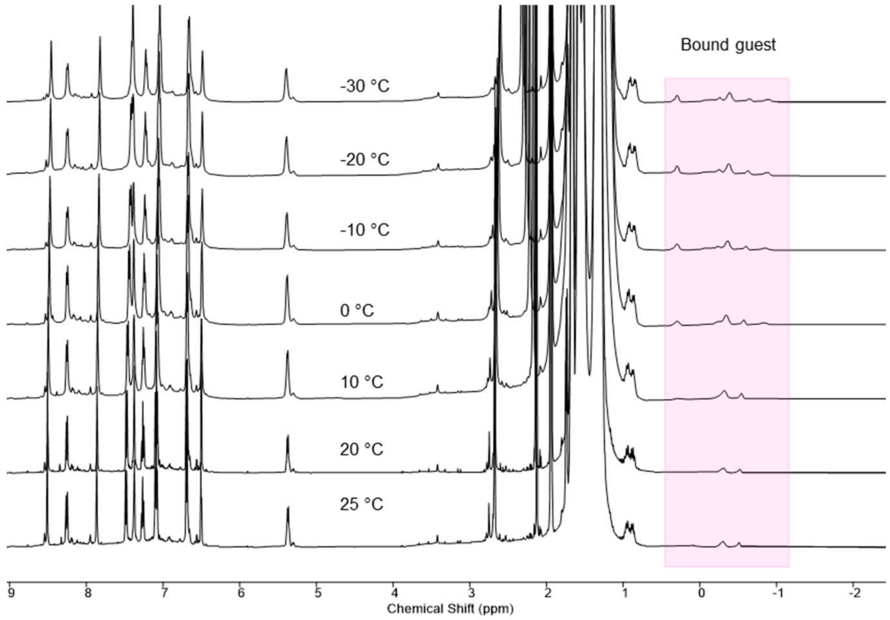

Figure S25. ^{1}H NMR spectra of **G1**⊂*Δ*$_4$-**1** in the presence of 128 equiv **G1** (128 equiv) at different temperatures (500 MHz, CD_3CN). Due to the small binding constants, a large excess of **G1** was needed to shift the equilibrium toward the formation of a clean host-guest complex.

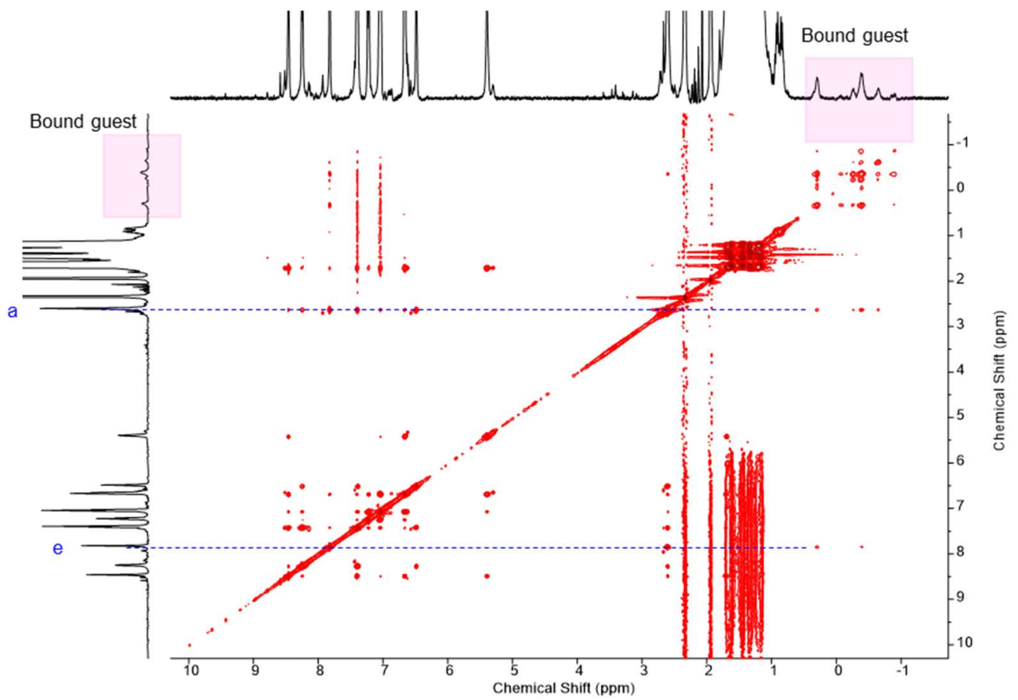

Figure S26. ^{1}H-^{1}H NOESY NMR spectrum of **G1**⊂*Δ*$_4$-**1** in the presence of 128 equiv **G1** (500 MHz, CD_3CN, −30 °C). Due to the small binding constants, a large excess of **G1** was needed to shift the equilibrium toward the formation of a clean host-guest complex.

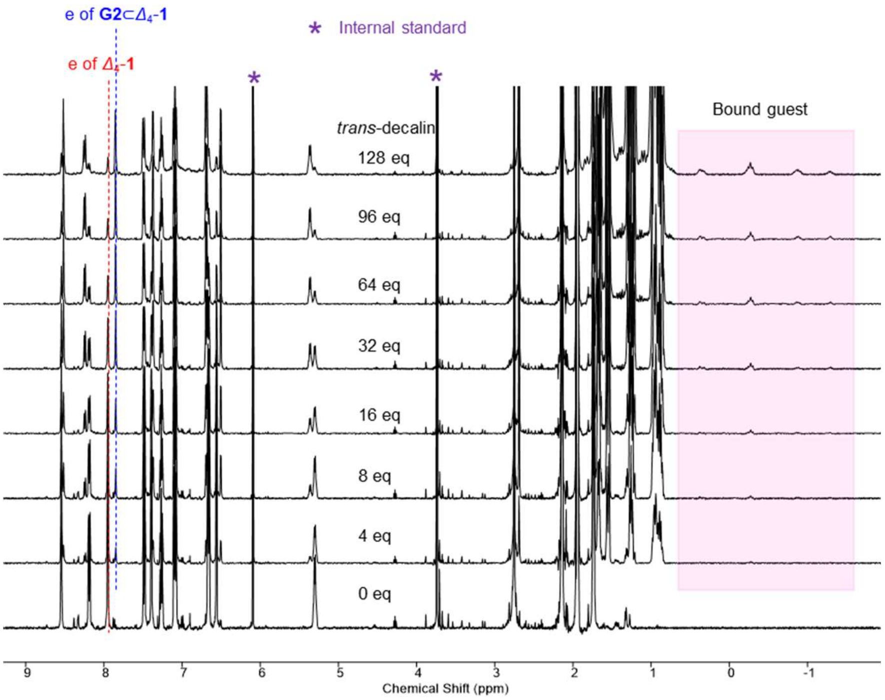

Figure 27. ^{1}H NMR spectra upon addition of **G2** (0.75 M in CD_3CN) into *Δ*$_4$-**1** using 1,3,5-trimethoxybenzene as internal standard (500 MHz, CD_3CN, 25 °C).

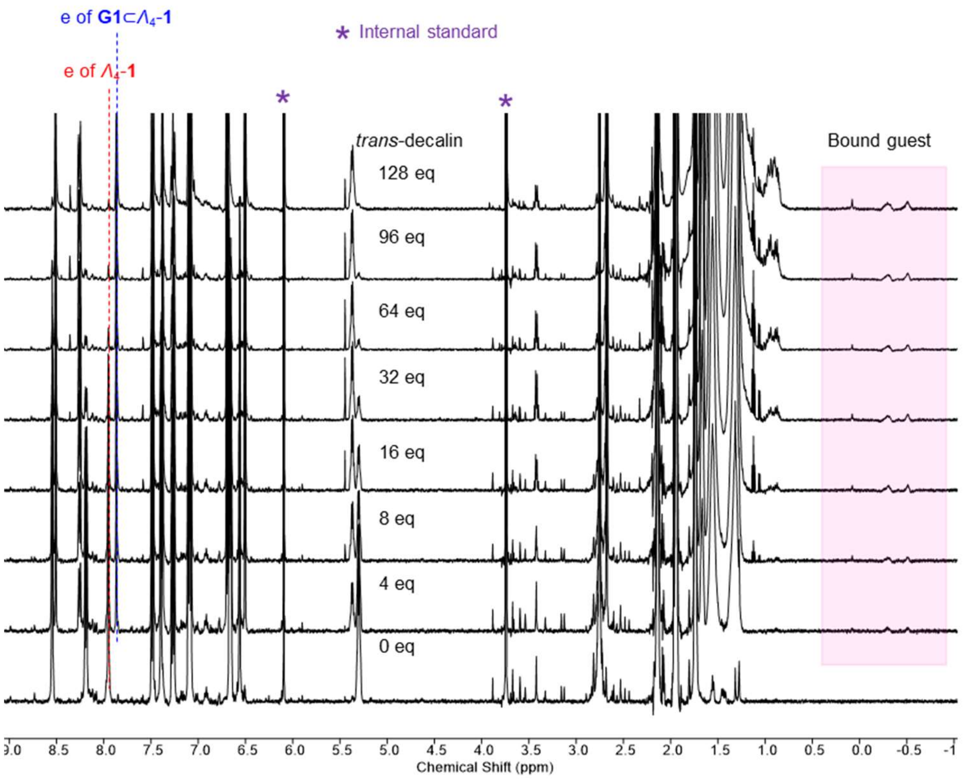

Figure 28. ^{1}H NMR spectra upon addition of **G1** (0.75 M in CD_3CN) into Λ_4-**1** using 1,3,5-trimethoxybenzene as internal standard (500 MHz, CD_3CN, 25 °C).

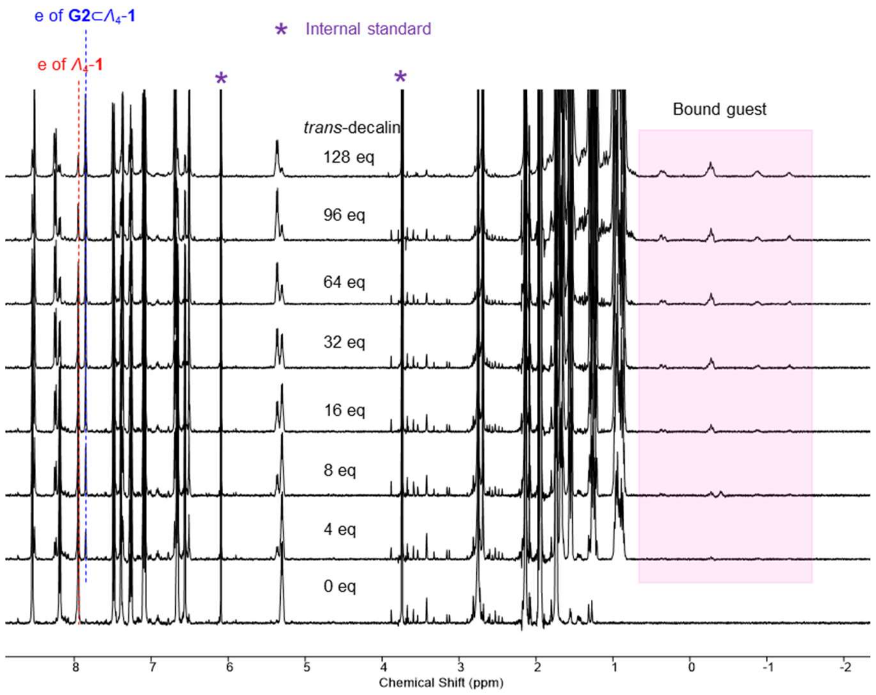

Figure 29. ^{1}H NMR spectra upon addition of **G2** (0.75 M in CD_3CN) into Λ_4-**1** using 1,3,5-trimethoxybenzene as internal standard (500 MHz, CD_3CN, 25 °C).

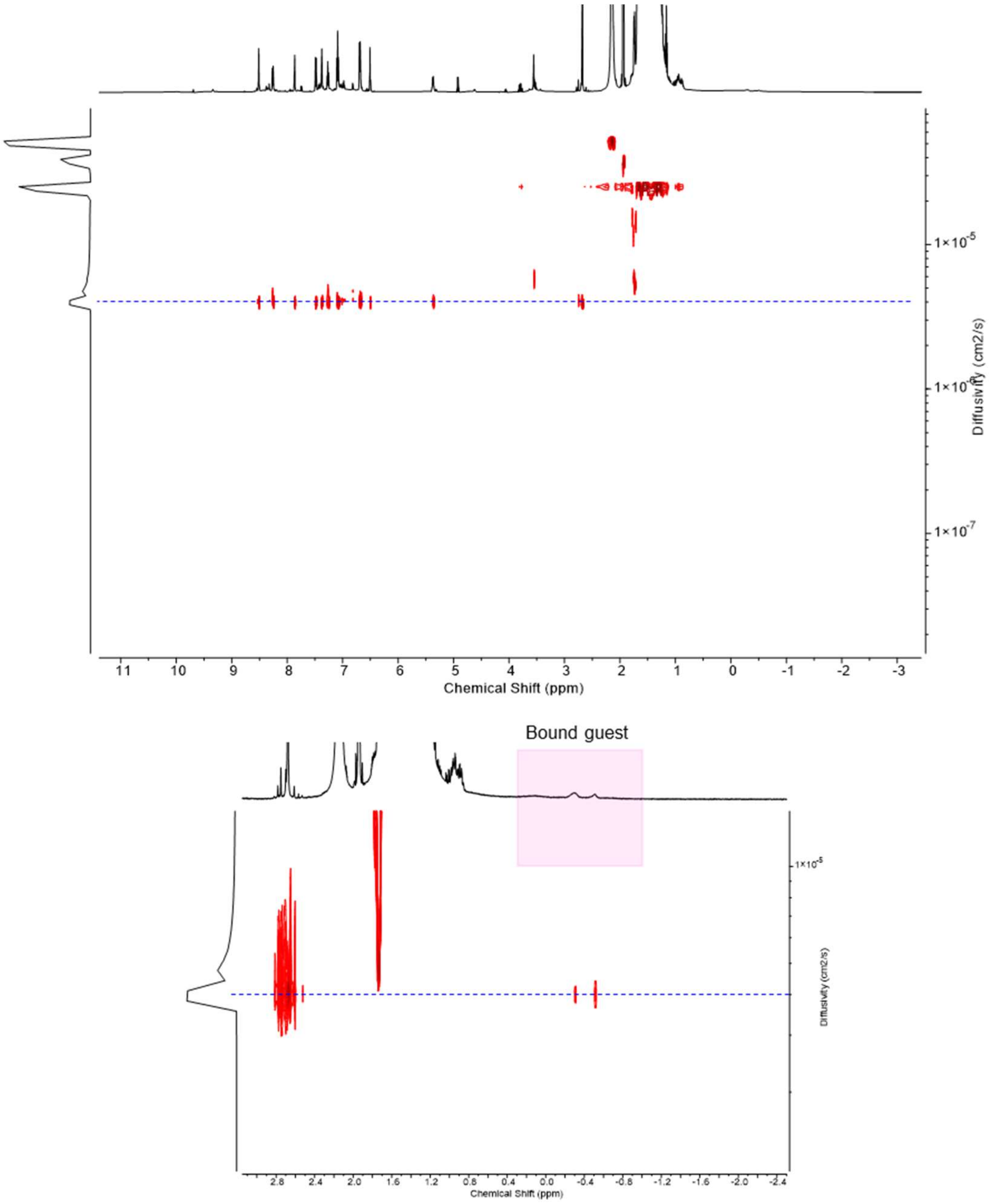

Figure S30. ^{1}H DOSY spectrum of $\mathbf{G1}\subset\Delta_4\text{-}\mathbf{1}$ in the presence of 128 equiv **G1** (500 MHz, CD_3CN, 25 °C), with bound guest region enlarged. The diffusion coefficient was measured to be 4.06×10^{-6} cm^2/s. Due to the small binding constants, a large excess of **G1** was needed to shift the equilibrium toward the formation of a clean host-guest complex.

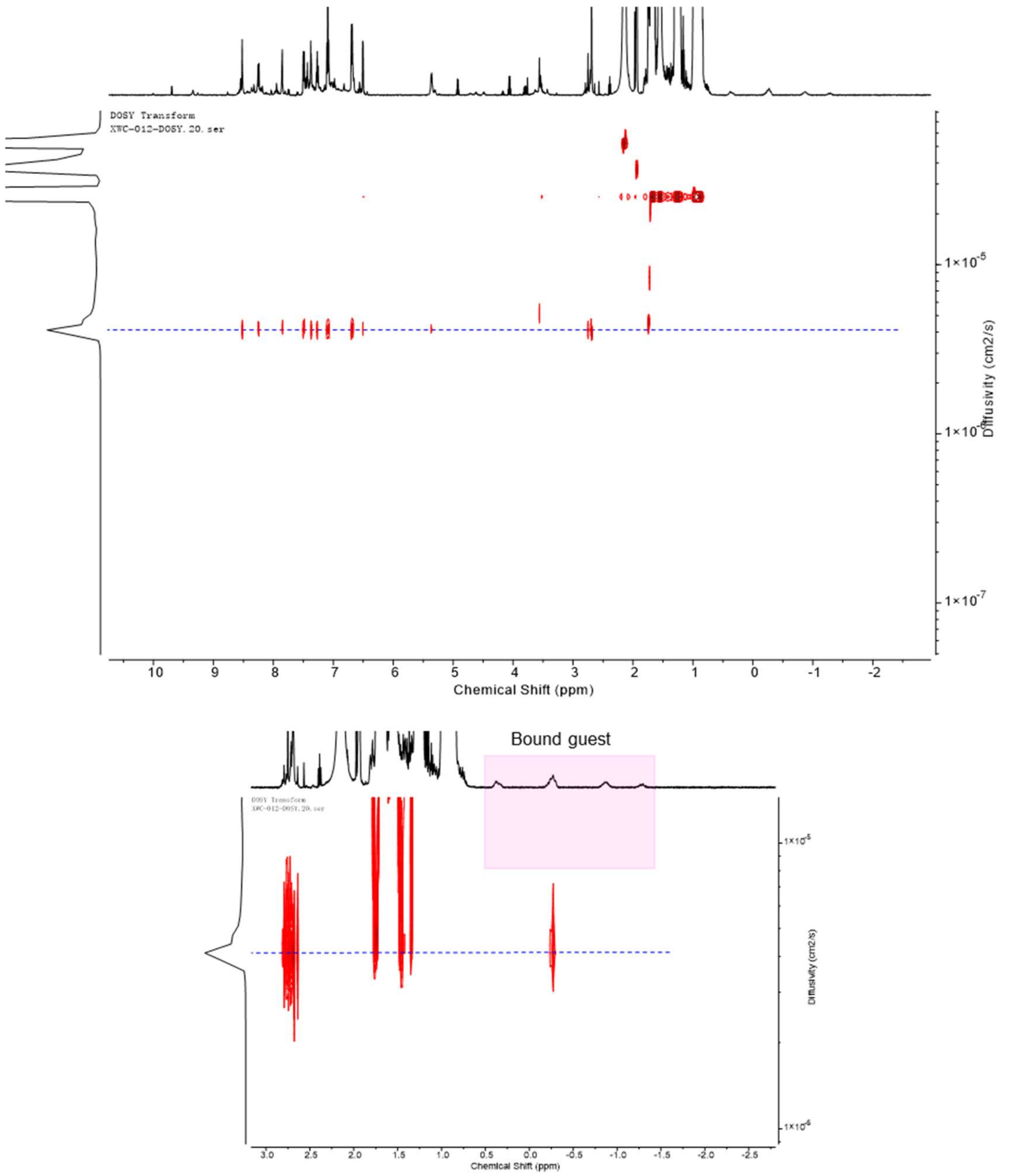

Figure S31. ^{1}H DOSY spectrum of **G2**⊂*Δ*$_4$**-1** in the presence of 128 equiv **G2** (500 MHz, CD_3CN, 25 °C), with bound guest region enlarged. The diffusion coefficient was measured to be 4.04×10^{-6} cm^2/s. Due to the small binding constants, a large excess of **G2** was needed to shift the equilibrium toward the formation of a clean host-guest complex.

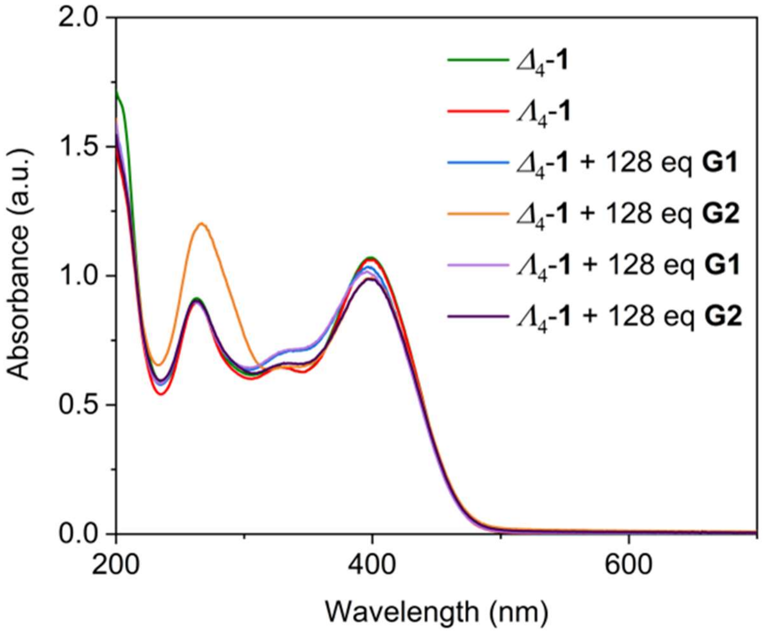

Figure S32. UV-vis spectra of Δ_4-**1**, Λ_4-**1** and their host-guest complexes in MeCN (50 μM of cage).

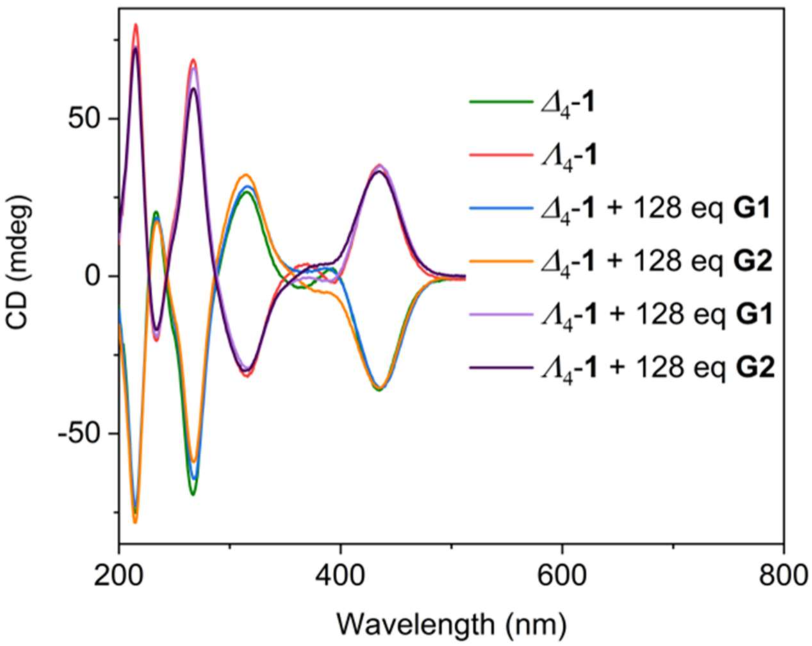

Figure S33. CD spectra of Δ_4-**1**, Λ_4-**1** and their host-guest complexes in MeCN (50 μM of cage).

4.2 Host-Guest Interaction of Enantiopure 1 with G3 and Its Carboxylate

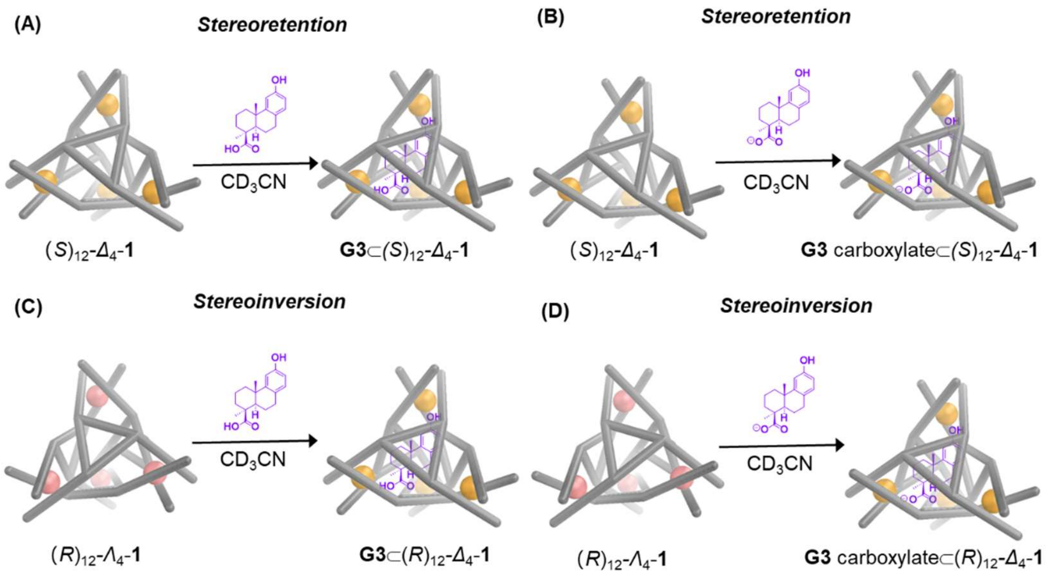

The binding of **G3** and its carboxylate by **1** was observed to be in slow exchange on the NMR timescale, as proton signals corresponding to host-guest complexes emerged as free **1** disappeared from the ^{1}H NMR spectrum during titration experiments. The formation of a 1 :1 host-guest complex was observed in HR-ESI-MS spectra. All proton signals from the bound guest shifted significantly upfield as a result of shielding effects, consistent with central binding of **G3**.

Both Δ_4-**1** and Λ_4-**1** were able to bind enantiopure **G3** and **G3** carboxylate, forming host-guest complexes with magnetically distinct ^{1}H NMR signals, consistent with the formation of diastereomeric complexes. In both cases, the proton signals of the host exhibited splitting and broadening after guest encapsulation, suggesting desymmetrization of the host framework on the NMR timescale. A single set of well-pronounced proton peaks was observed for bound guest, indicating that both host-guest complexes are enantiopure. $(S)_{12}$-Δ_4-**1** bound **G3** and its carboxylate in a stereoretentive manner, producing **G3**⊂$(S)_{12}$-Δ_4-**1** and **G3** carboxylate⊂$(S)_{12}$-Δ_4-**1**, respectively.[3] On the other hand, $(R)_{12}$-Λ_4-**1** dynamically inverted its stereochemistry of Zn^{II} centers and ligand panel orientation upon encapsulating **G3** and its carboxylate, giving **G3**⊂$(R)_{12}$-Δ_4-**1** and **G3** carboxylate⊂$(R)_{12}$-Δ_4-**1**, respectively.

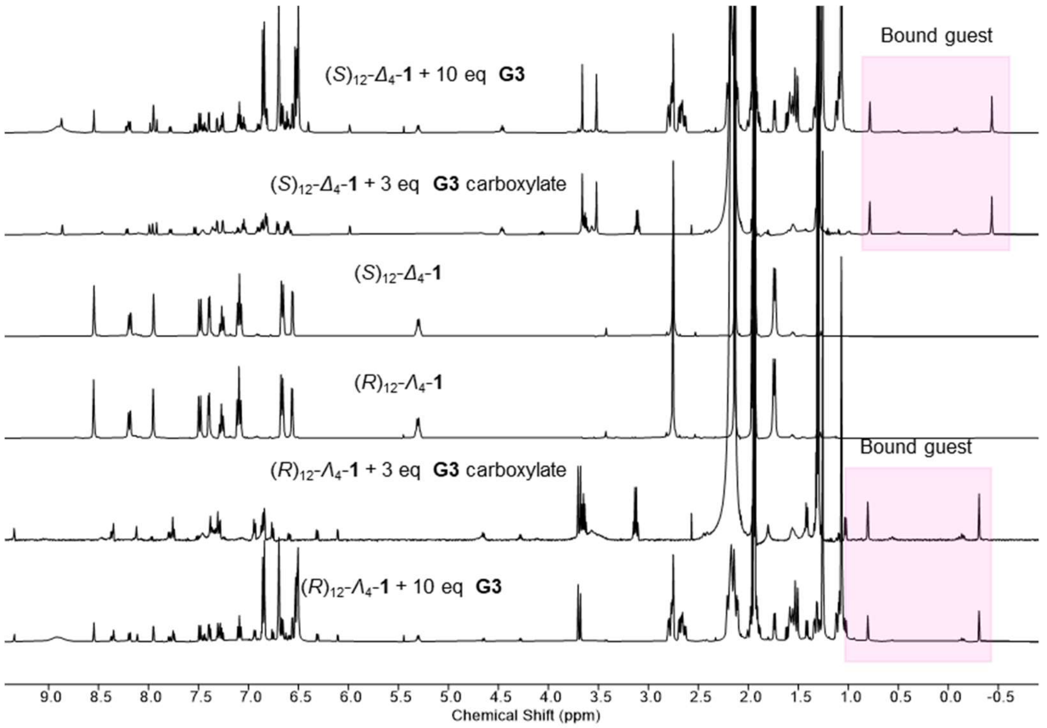

Figure S34. ^{1}H NMR spectra of $\mathit{\Delta}_4$-**1**, $\mathit{\Lambda}_4$-**1** and their host-guest complexes in MeCN. (500 MHz, CD_3CN, 25 °C).

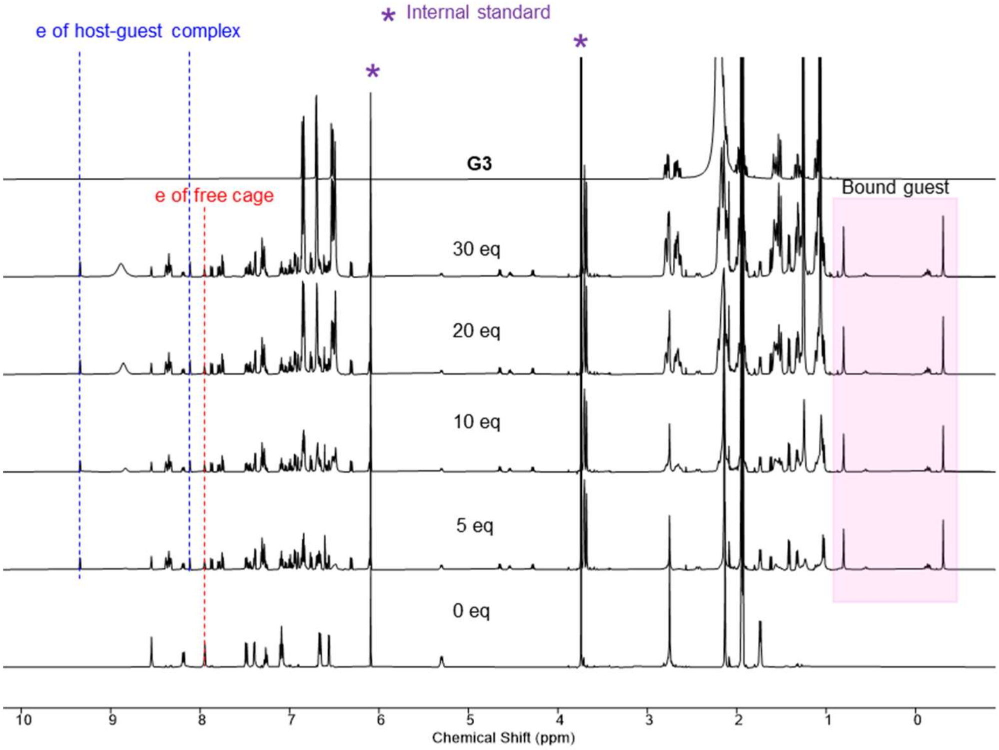

Figure S35. ^{1}H NMR spectra upon addition of **G3** as solid into (S)$_{12}$-$\mathit{\Delta}_4$-**1** using 1,3,5-trimethoxybenzene as internal standard (500 MHz, CD_3CN, 25 °C).

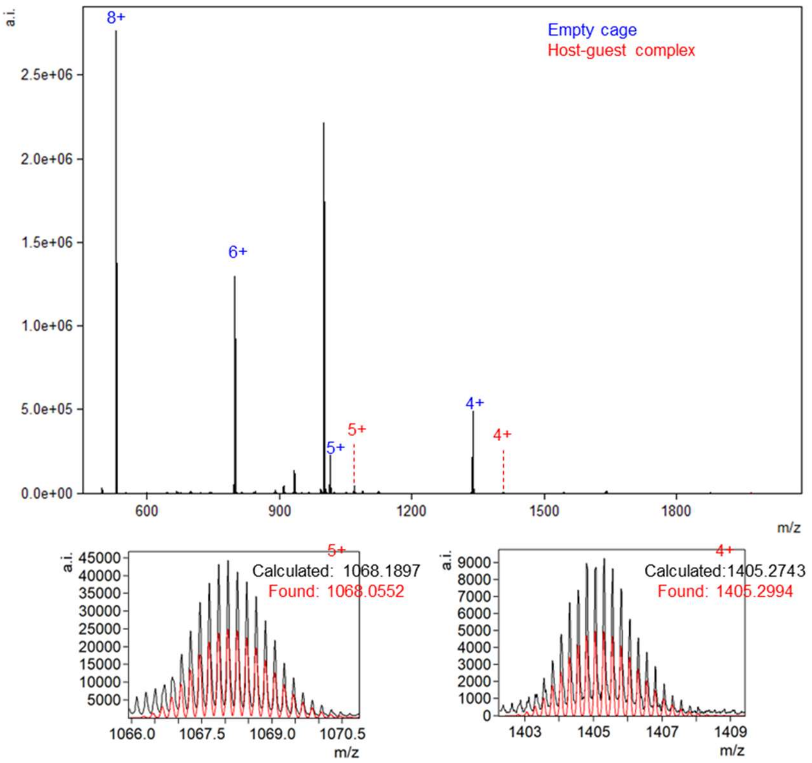

Figure S36. HR-ESI-MS spectrum of (S)$_{12}$-Δ_4-**1** in presence of 10 equiv **G3** in MeCN.

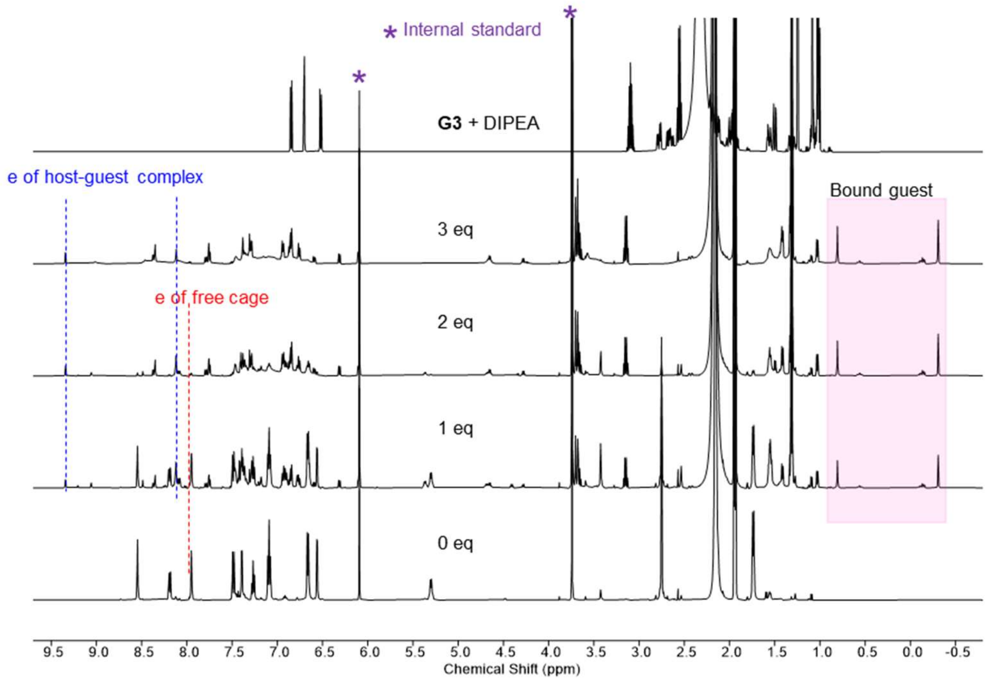

Figure S37. ^{1}H NMR spectra upon addition of **G3** carboxylate (37.5 mM in CD_3CN) into (S)$_{12}$-Δ_4-**1** using 1,3,5-trimethoxybenzene as internal standard (500 MHz, CD_3CN, 25 °C). Minor decomposition of the cage was observed during titrations as a result of the presence of DIPEA.

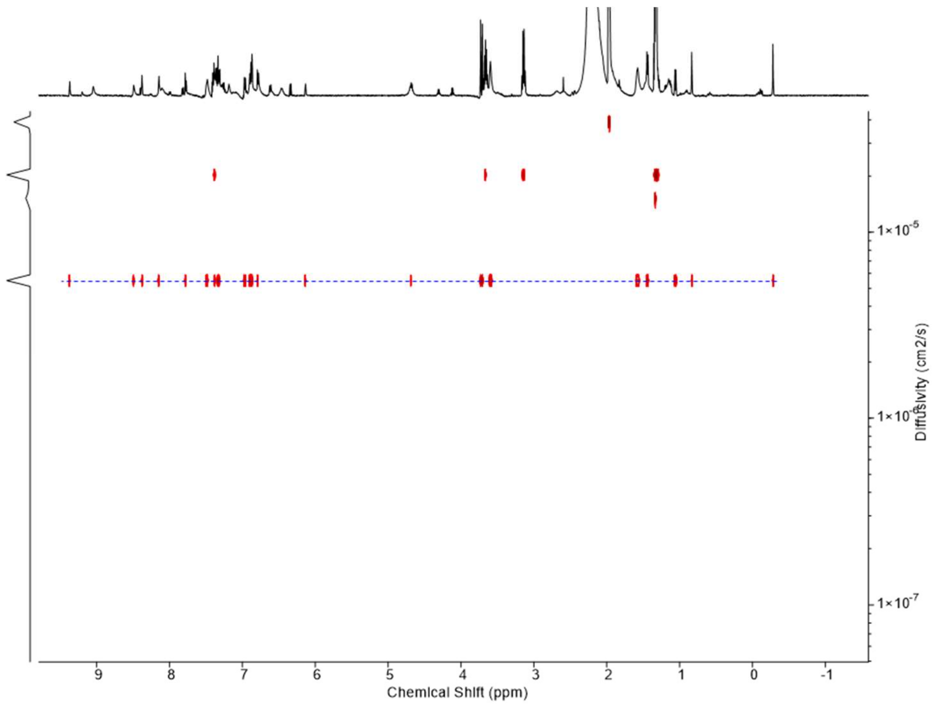

Figure S38. ^{1}H DOSY spectrum of **G3** carboxylate⊂(S)$_{12}$-Δ_4-**1** in the presence of 3 equiv **G3** carboxylate (500 MHz, CD_3CN, 25 °C). The diffusion coefficient was measured to be 5.25×10^{-6} cm^2/s.

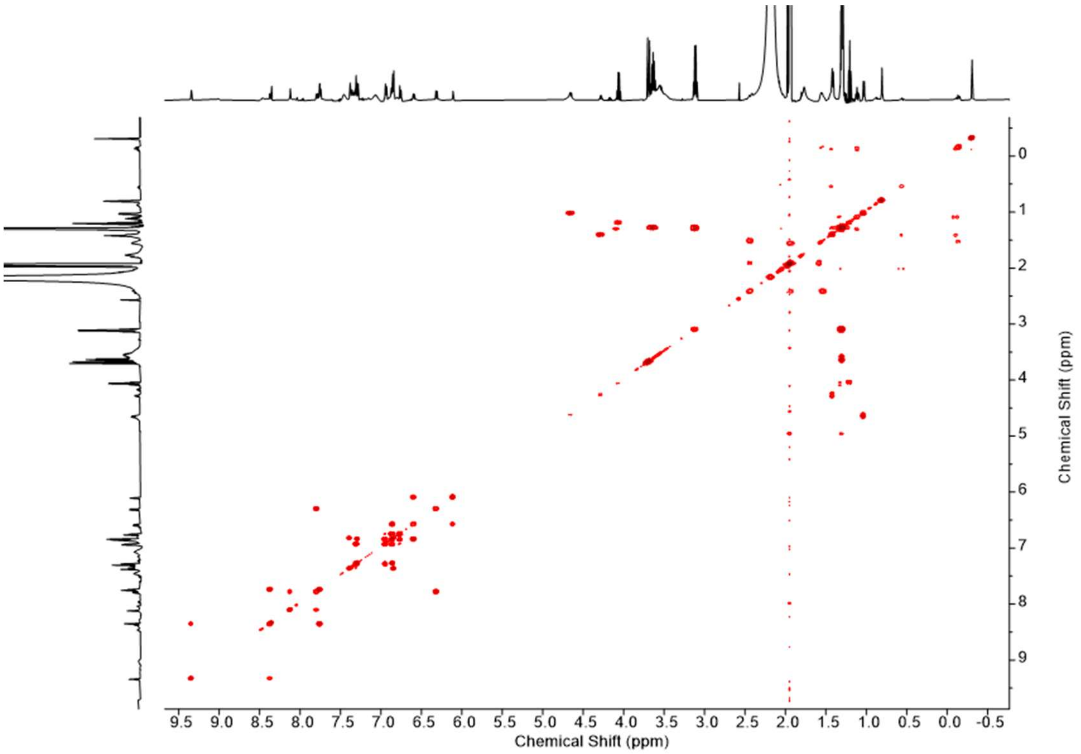

Figure 39. ^{1}H-^{1}H COSY NMR spectrum of **G3** carboxylate⊂(S)$_{12}$-Δ_4-**1** (500 MHz, CD_3CN, 25 °C).

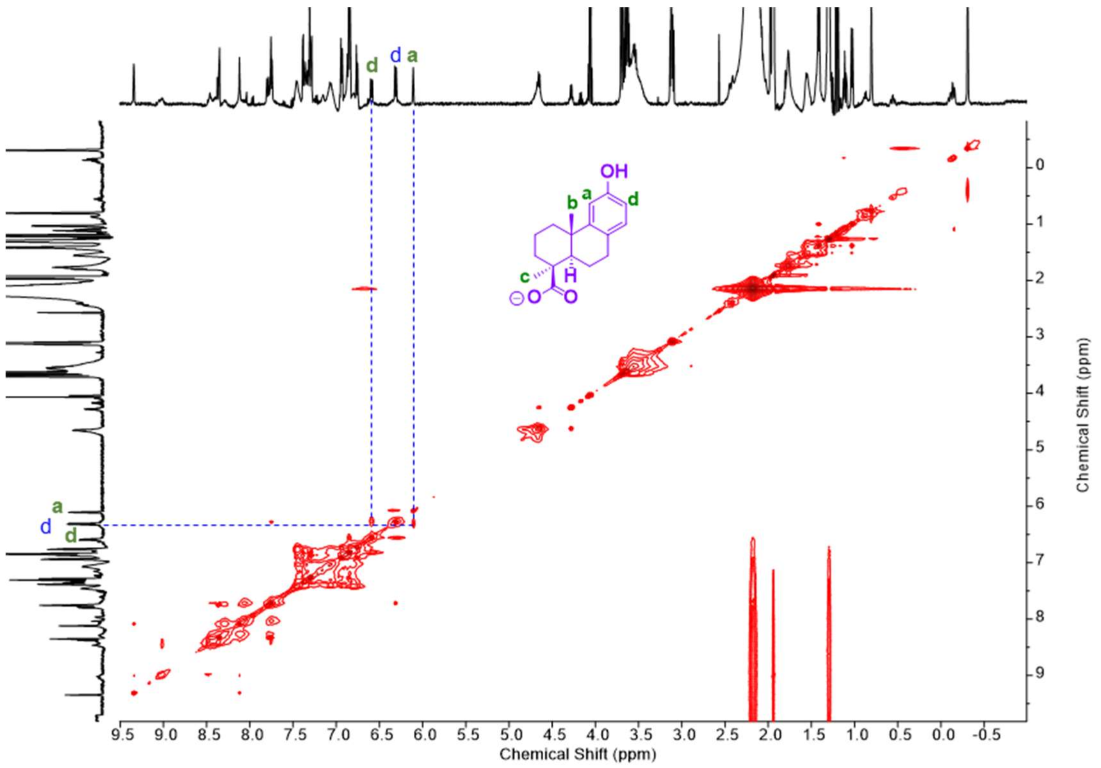

Figure 40. ^{1}H-^{1}H NOESY NMR spectrum of **G3** carboxylate⊂(S)$_{12}$-Δ_4-**1** (500 MHz, CD_3CN, 25 °C).

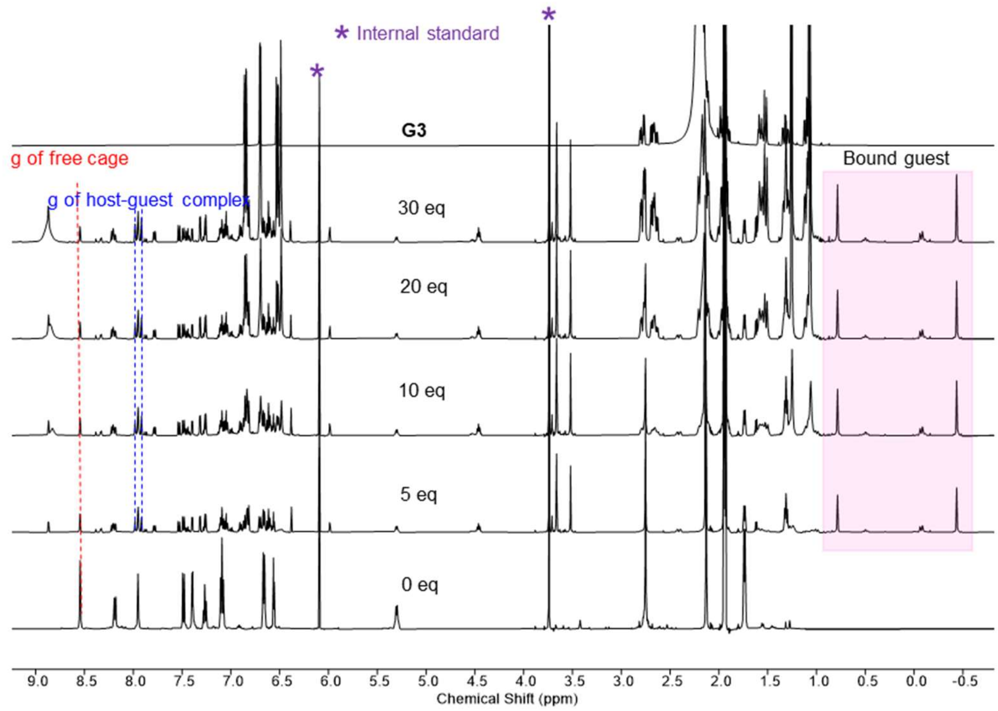

Figure S41. ^{1}H NMR spectra upon addition of **G3** as solid into (R)$_{12}$-Λ_4-**1** (500 MHz, CD_3CN, 25 °C).

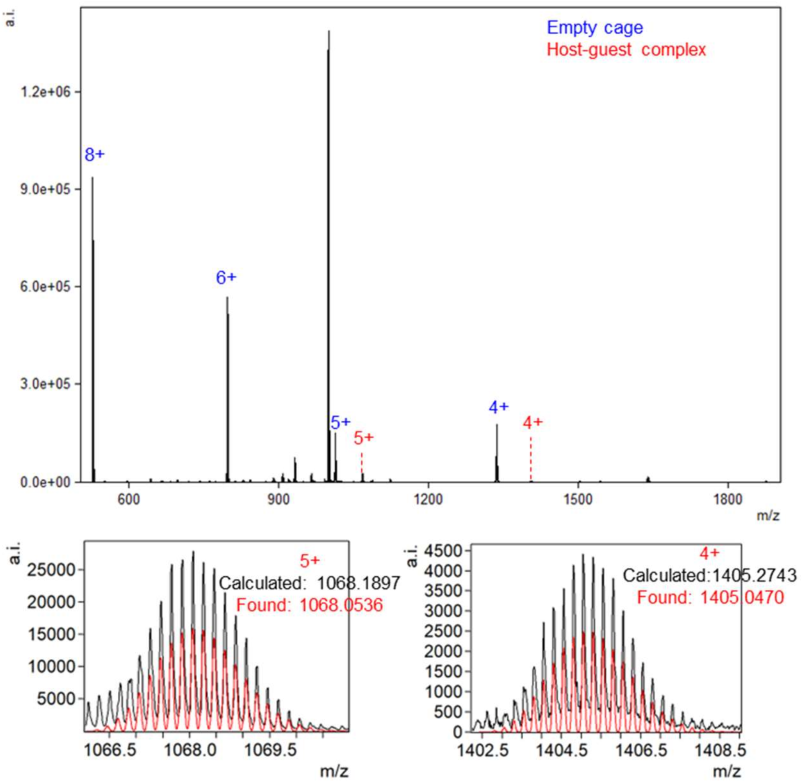

Figure S42. HR-ESI-MS spectrum of (R)$_{12}$-Λ_4-**1** in presence of 10 equiv **G3** in MeCN.

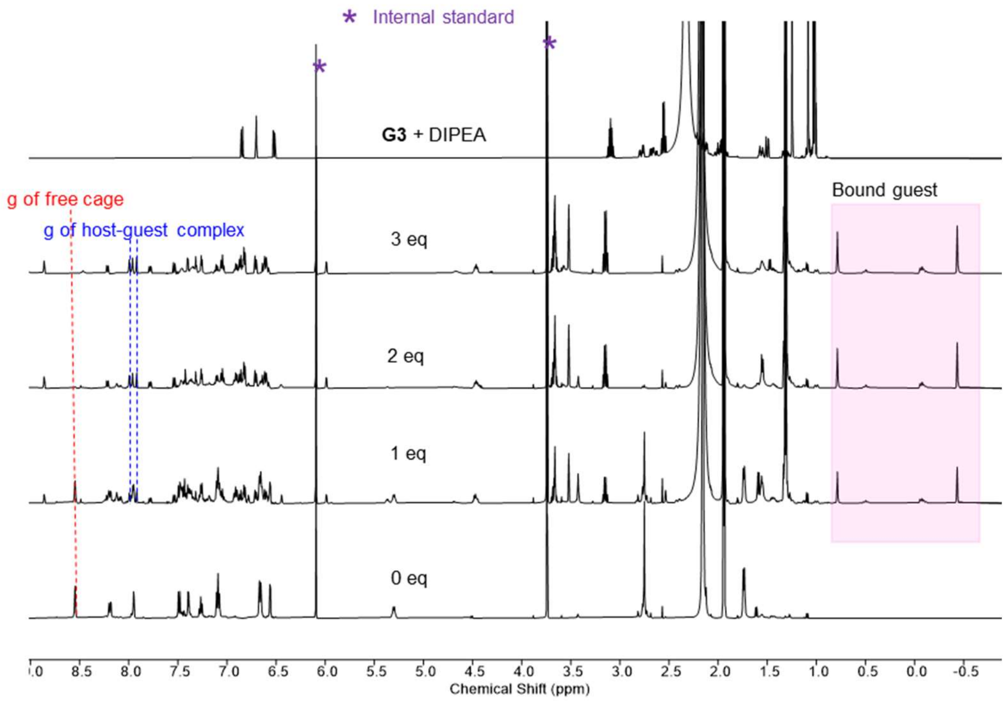

Figure S43. ^{1}H NMR spectra upon addition of **G3** carboxylate (37.5 mM in CD_3CN) into (R)$_{12}$-Λ_4-**1** (500 MHz, CD_3CN, 25 °C). Minor decomposition of the cage was observed during titrations as a result of the presence of DIPEA.

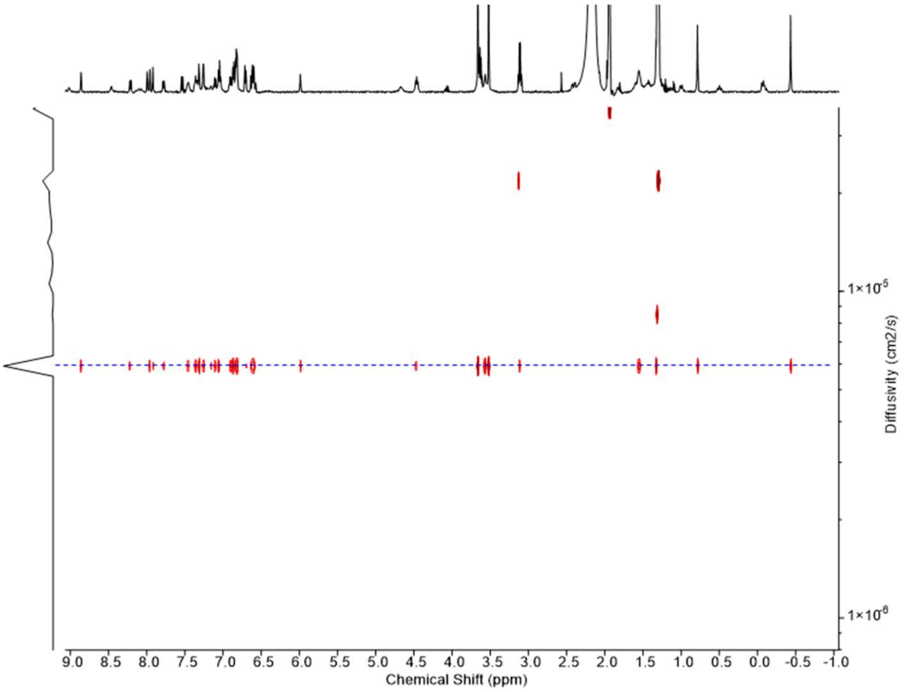

Figure S44. ^{1}H DOSY spectrum of **G3** carboxylate⊂(*R*)$_{12}$-*Δ*$_{4}$-**1** in the presence of 3 equiv **G3** carboxylate (500 MHz, CD_3CN, 25 °C). The diffusion coefficient was measured to be 5.20×10^{-6} cm^{2}/s.

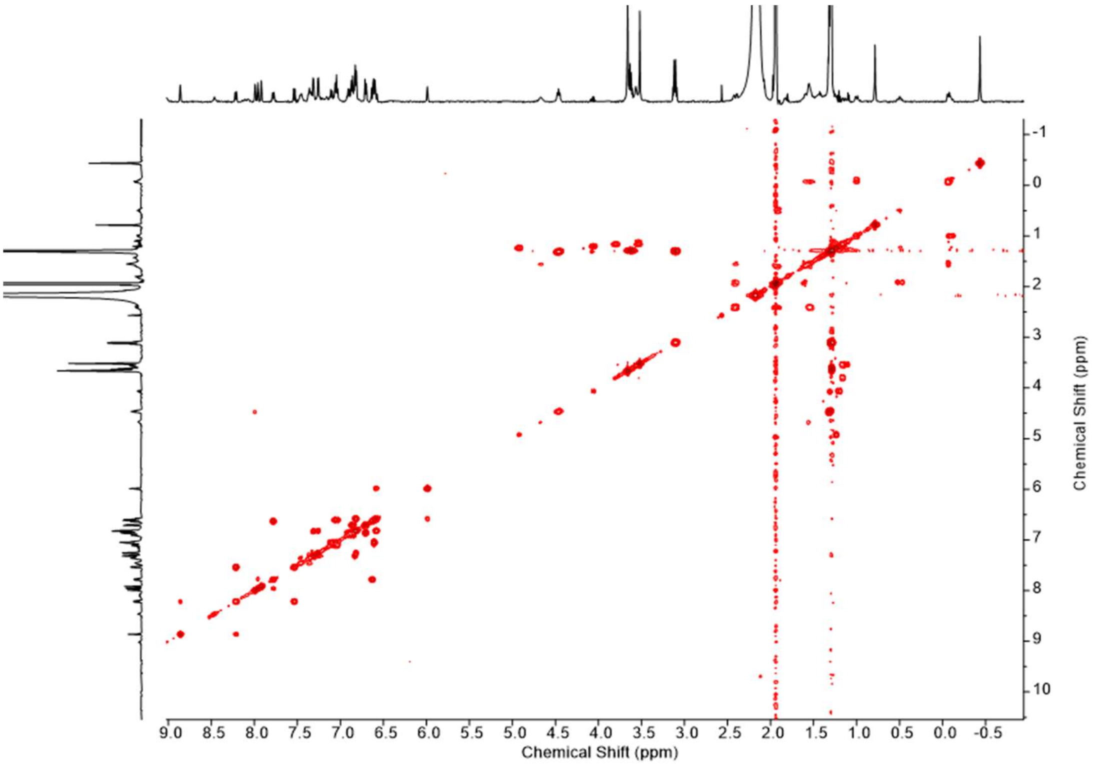

Figure 45. ^{1}H-^{1}H COSY NMR spectrum of **G3** carboxylate⊂(*R*)$_{12}$-*Δ*$_{4}$-**1** (500 MHz, CD_3CN, 25 °C).

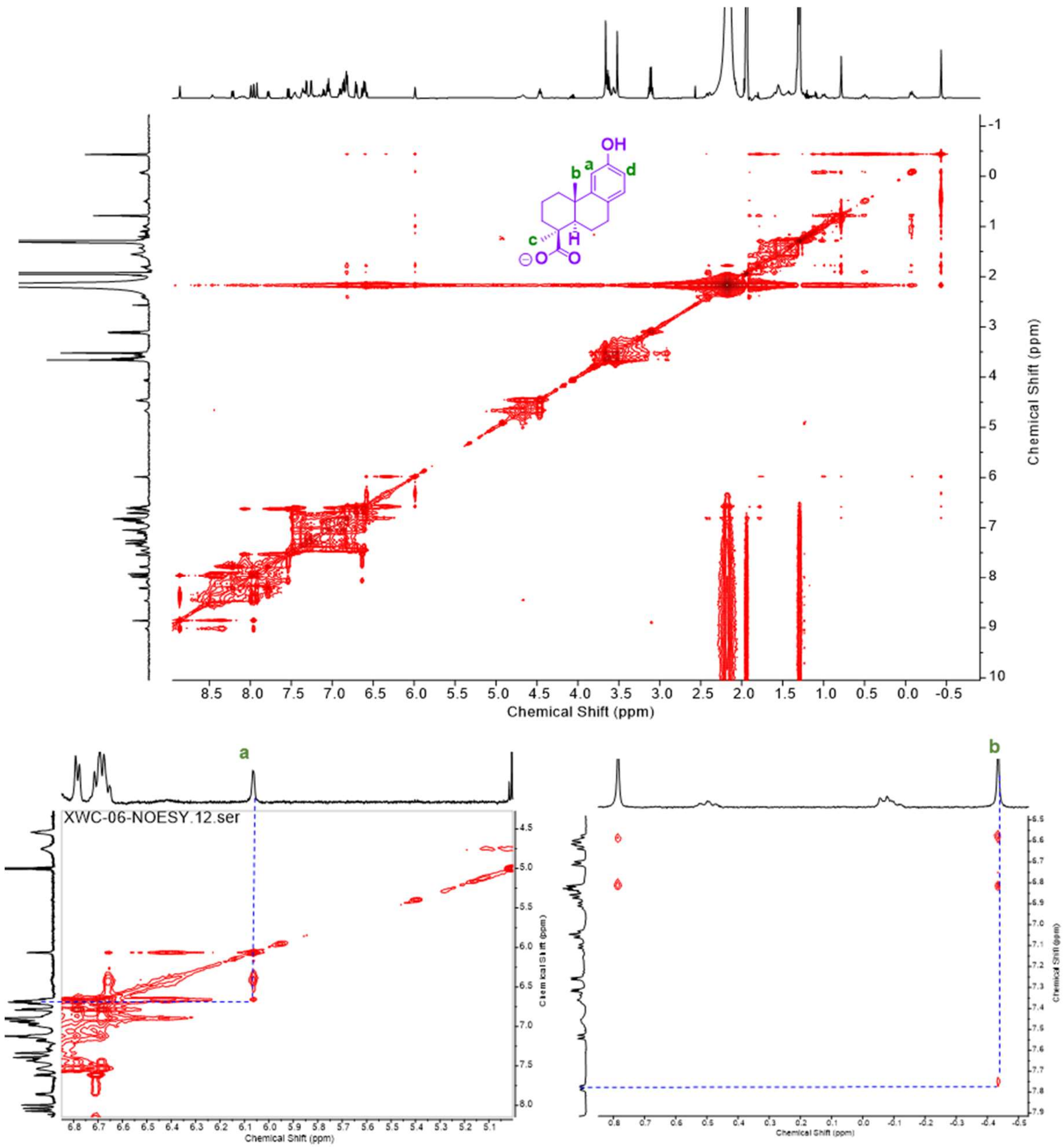

Figure 46. ^{1}H-^{1}H NOESY NMR spectrum of **G3** carboxylate⊂$(R)_{12}$-Δ_4-**1** (500 MHz, CD_3CN, 25 °C), with the key regions enlarged and highlighted.

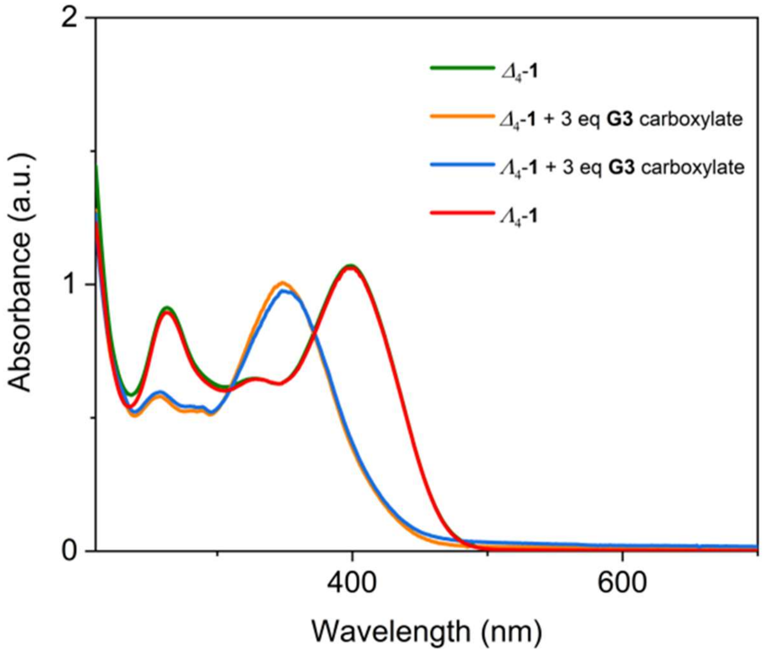

Figure S47. UV-vis spectra of Δ_4-**1**, Λ_4-**1** and their host-guest complexes in MeCN (50 μM of cage).

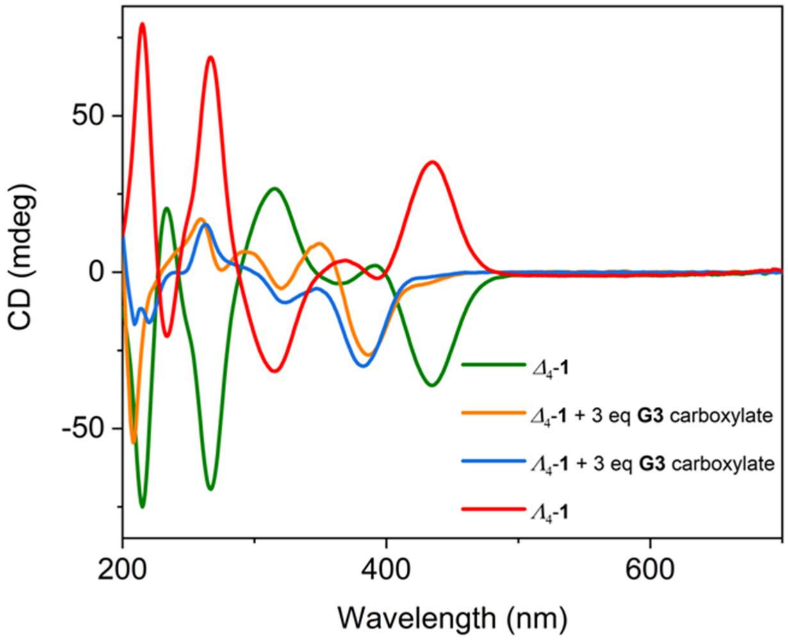

Figure S48. CD spectra of Δ_4-**1**, Λ_4-**1** and their host-guest complexes in MeCN (50 μM of cage).

4.3 Host-Guest Interactions of Racemic 2 with G1, G2, and G3 Carboxylate

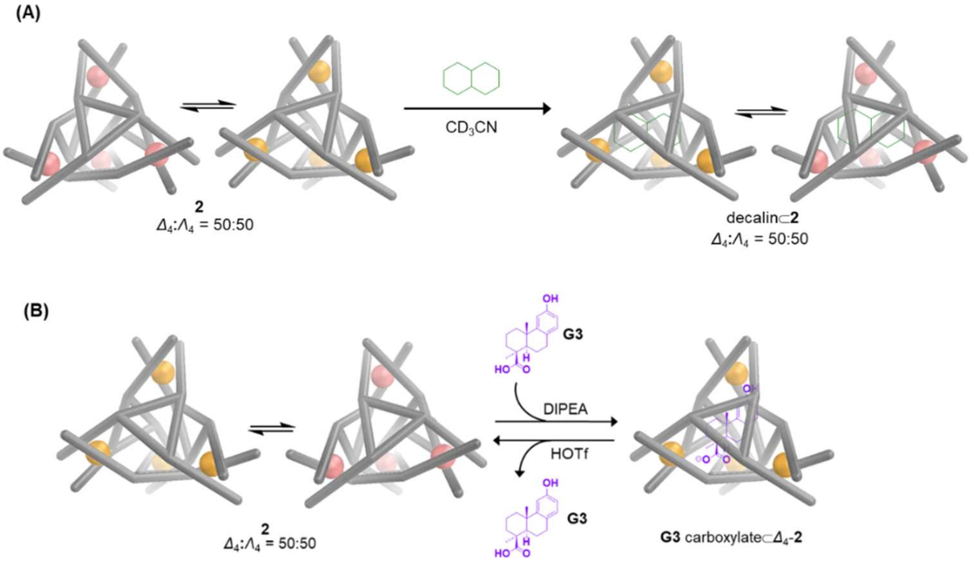

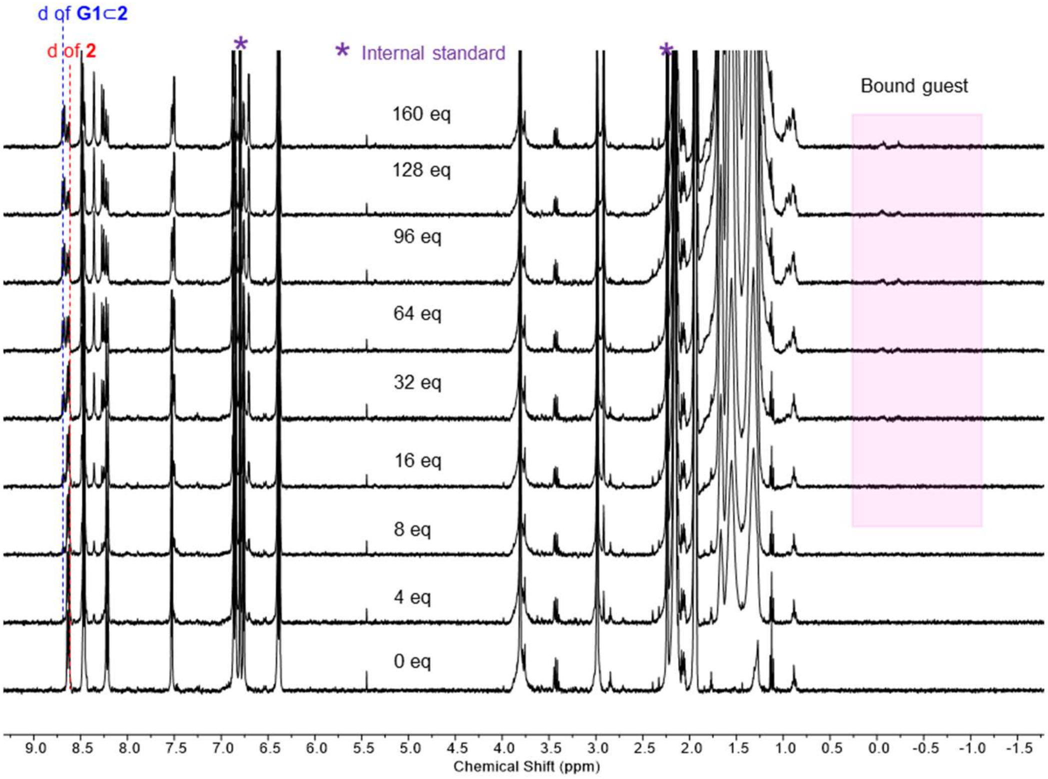

Figure S49. ^{1}H NMR spectra following addition of **G1** (0.75 M in CD_3CN) to **2** using 1,3,5-trimethylbenzene as the internal standard (400 MHz, CD_3CN, 25 °C).

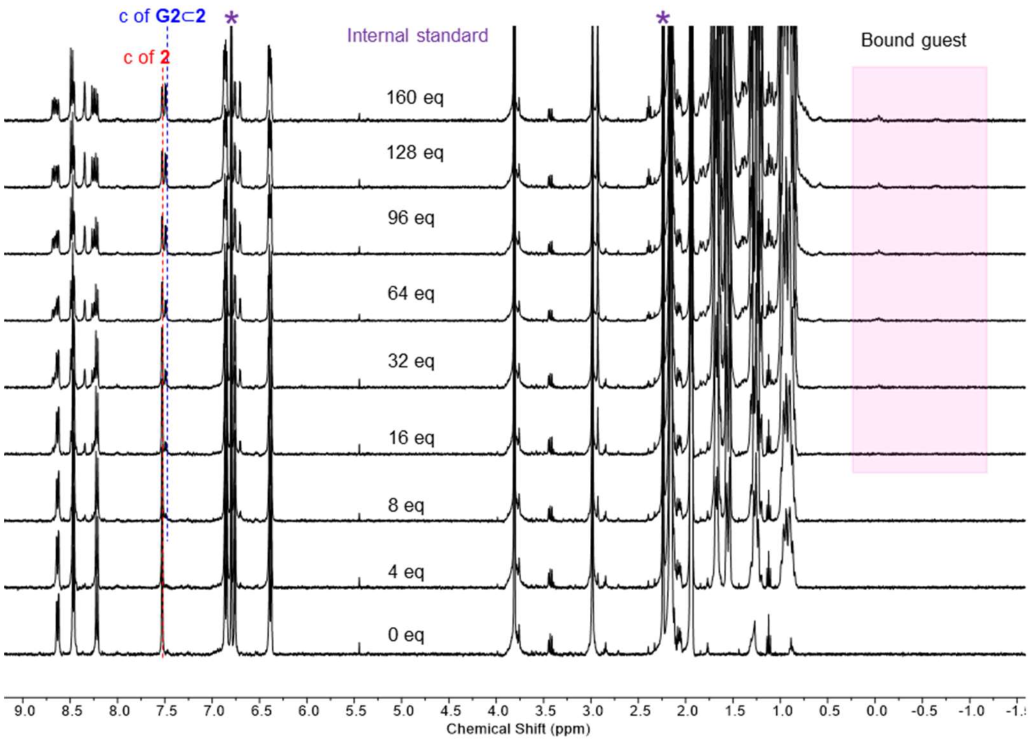

Figure S50. ^{1}H NMR spectra following addition of **G2** (0.75 M in CD_3CN) to **2** using 1,3,5-trimethylbenzene as the internal standard (400 MHz, CD_3CN, 25 °C).

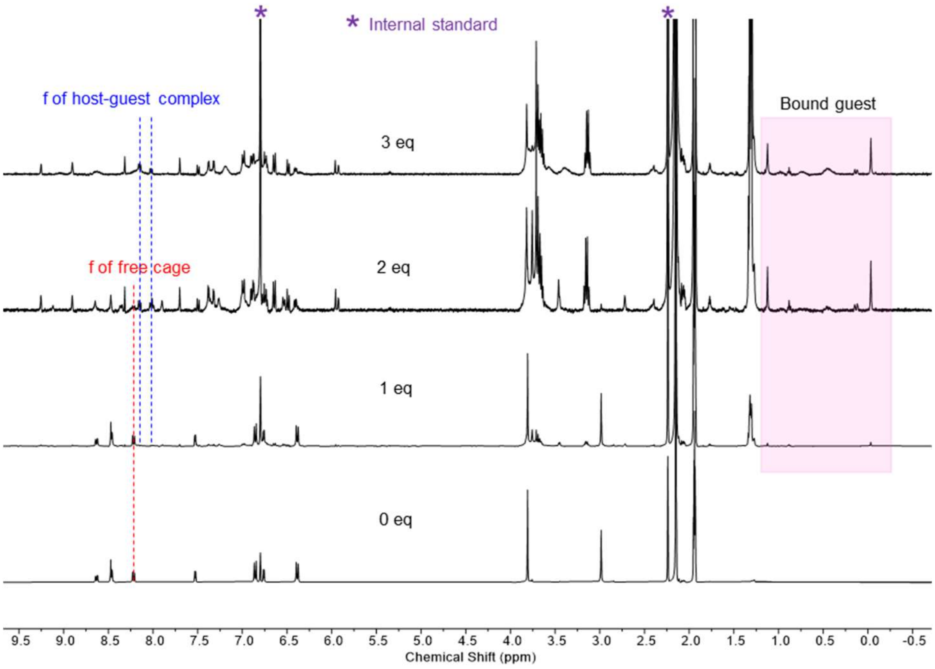

Figure S51. ^{1}H NMR spectra following addition of **G3** carboxylate (37.5 mM in CD_3CN) into **2** using 1,3,5-trimethylbenzene as the internal standard (400 MHz, CD_3CN, 25 °C).

Guest uptake and release: Addition of 3 equivalents of **G3** into the CD_3CN solution of **2** (0.75 mM) did not lead to proton peaks corresponding to bound guests or any shifts of host signals, indicating that **2** was not able to encapsulate neutral **G3**. As a CD_3CN solution of DIPEA (3 equiv) was progressively added to the mixture, enantiopure **G3** carboxylate⊂Δ_4-**2** formed after being kept at 343 K for 30 min without free **2** remaining, as confirmed by ^{1}H NMR and CD spectra. Subsequently, progressive addition of a CD_3CN solution of triflic acid (HOTf, 3 equiv) into the mixture resulted in guest release upon sonicating or shaking the reaction mixture at room temperature. The CD spectrum of this mixture was immediately recorded after guest release, and no Cotton effects were observed for **2**, indicating rapid racemization of host framework upon guest release.

The guest uptake and release process could be repeated over two cycles. In the third cycle, decomposition and precipitation of the cage were observed.

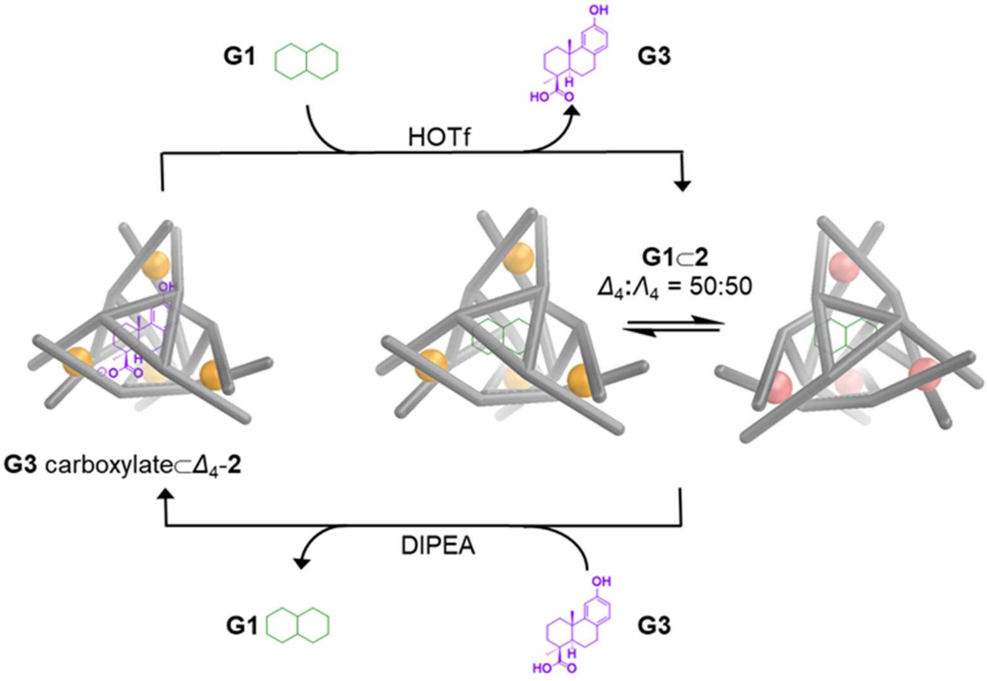

Guest exchange: A CD_3CN solution of HOTf (3 equiv) was progressively added to the CD_3CN solution of enantiopure **G3** carboxylate⊂Δ_4-**2** in the presence of excess **G1**. **G1⊂2** formed upon shaking the reaction mixture at room temperature without **G3** carboxylate⊂Δ_4-**2** remaining, as confirmed by ^{1}H NMR and CD spectra. The CD

spectrum of this mixture was immediately recorded after guest exchange, and no Cotton effects were observed for **G1**⊂**2**, indicating rapid racemization of host framework upon guest exchange. Subsequently, progressive addition of a CD_3CN solution of DIPEA (3 equiv) into the mixture resulted in the reformation of enantiopure **G3** carboxylate⊂Δ_4-**2** upon heating the reaction mixture at 343 K for 30 min. The guest exchange process could be repeated over two cycles.

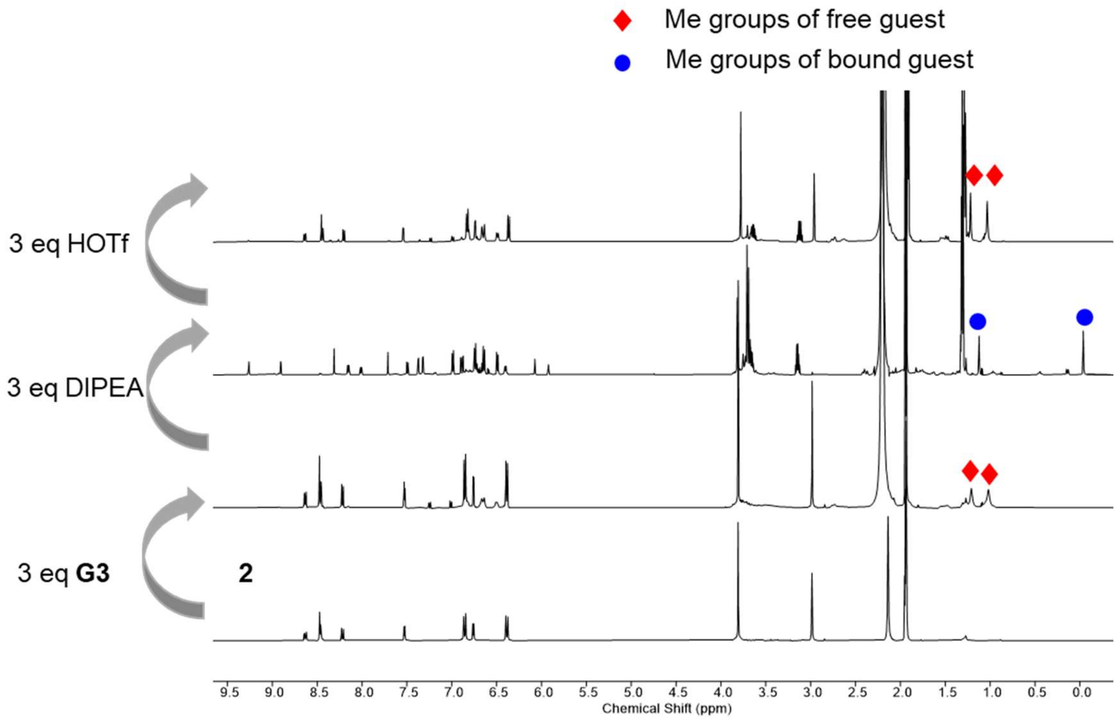

Figure S52. ^{1}H NMR spectra of **2** upon adding **G3**, acid and base (500 MHz, CD_3CN, 25 °C).

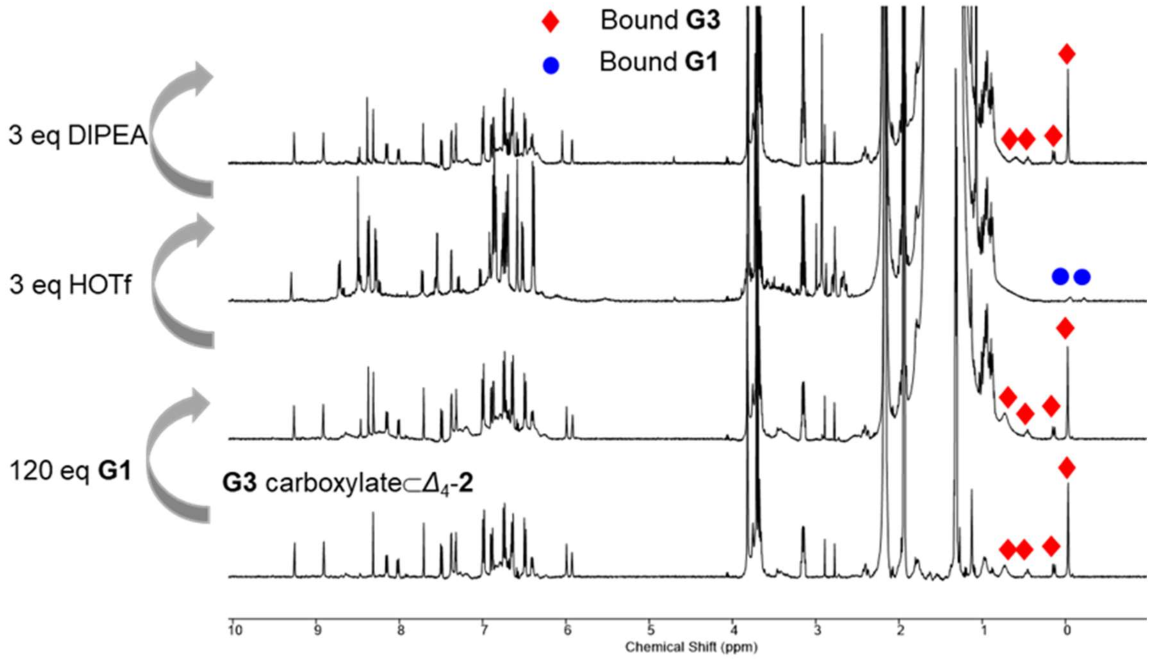

Figure S53. ^{1}H NMR spectra of **G3** carboxylate⊂Δ_4-**2** upon adding **G1**, acid and base in the presence of **G1** and **G3** (500 MHz, CD_3CN, 25 °C).

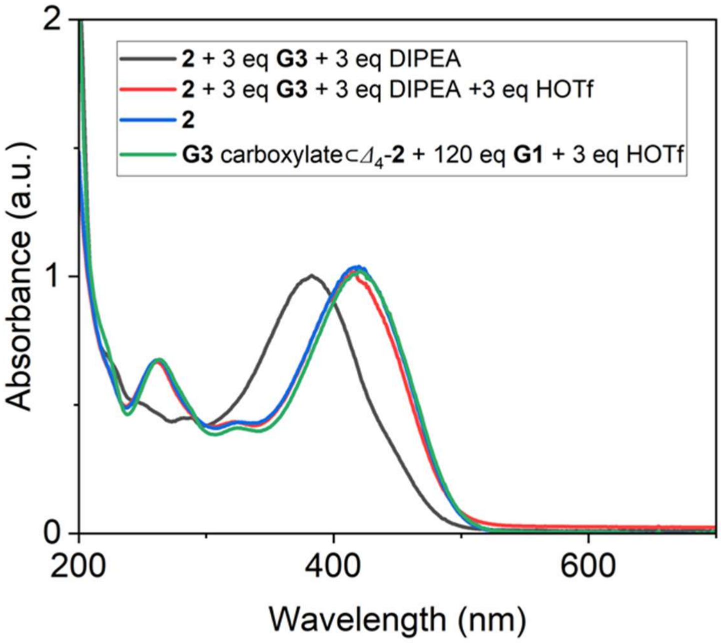

Figure S54. UV-vis spectra of **2** and its host-guest complexes upon adding guest, acid and base in MeCN (50 μM of cage).

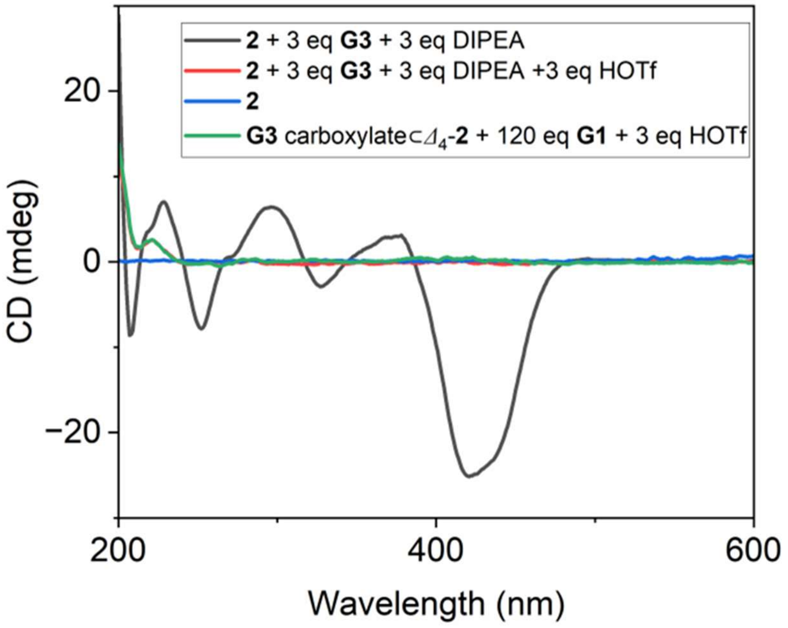

Figure S55. CD spectra of **2** and its host-guest complexes upon adding guest, acid and base in MeCN (50 µM of cage).

4.4 Guest Binding Studies through ITC Experiments

In addition to NMR titrations, ITC experiments were conducted to explore the thermodynamic aspects of the complexation processes using cages **1** and **2**. During these experiments, an acetonitrile solution of the guests (37.5 mM for **G1** and **G2**; 18.75 mM for **G3** carboxylate) was progressively added to a 300 μL solution of the host in acetonitrile (0.75 mM) within a sample cell.

The overall binding constants by ITC are one magnitude larger than those by NMR titrations. The binding constants for **G2** with cages **1** and **2**, as well as for **G1** with cage **2**, were determined to be too small to be detected within the instrument's offline detection range (10^3 M^{-1}).

The trends observed in ITC experiments are consistent with the data from NMR: **G1** was observed to be bound more strongly than **G2**; Δ_4-**1** exhibited a higher binding affinity for the **G3** carboxylate compared to Λ_4-**1**. In all experiments, the binding process was found to be both enthalpically and entropically favorable.

Table S2. Summary of thermodynamic data determined by ITC experiments.

Guest	Host	Binding Constant (M^{-1})	ΔG (kJ mol^{-1})	ΔH (kJ mol^{-1})	ΔS (J mol^{-1})
**G1**	Δ_4-**1**	$(1.67 \pm 0.22) \times 10^3$	-28.4 ± 3.4	-12.2 ± 3.4	54.3
**G1**	Λ_4-**1**	$(1.82 \pm 0.38) \times 10^3$	-18.6 ± 0.9	-7.7 ± 0.9	36.7
**G1**	**2**	$< 1.00 \times 10^3$	-17.1 ± 0.3	-6.4 ± 0.3	36.0
**G2**	Δ_4-**1**	$< 1\times 10^3$	-23.8 ± 0.04	-8.0 ± 0.04	53.2
**G2**	Λ_4-**1**	$< 1 \times 10^3$	-17.2 ± 0.1	-2.5 ± 0.1	49.2
**G2**	**2**	$< 1 \times 10^3$	-17.2 ± 0.05	-1.7 ± 0.05	51.9
**G3** carboxylate	Δ_4-**1**	$(4.26 \pm 0.60) \times 10^4$	-46.4 ± 4.4	-22.6 ± 4.4	79.9
**G3** carboxylate	Λ_4-**1**	$(2.49 \pm 0.34) \times 10^4$	-45.1 ± 3.3	-23.4 ± 3.3	72.8
**G3** carboxylate	**2**	$(1.81 \pm 0.13) \times 10^4$	-44.3 ± 4.3	-23.8 ± 4.3	68.8

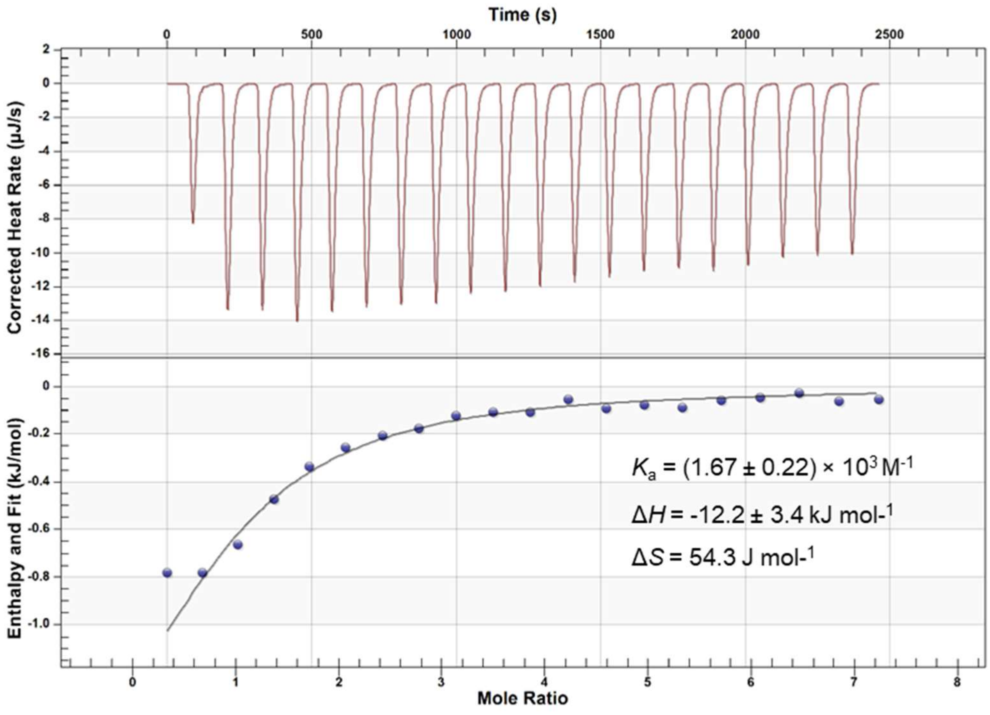

Figure S56. ITC titration plots and fitted curves (25 °C, MeCN) obtained by titration of **G1** into Δ_4-**1**.

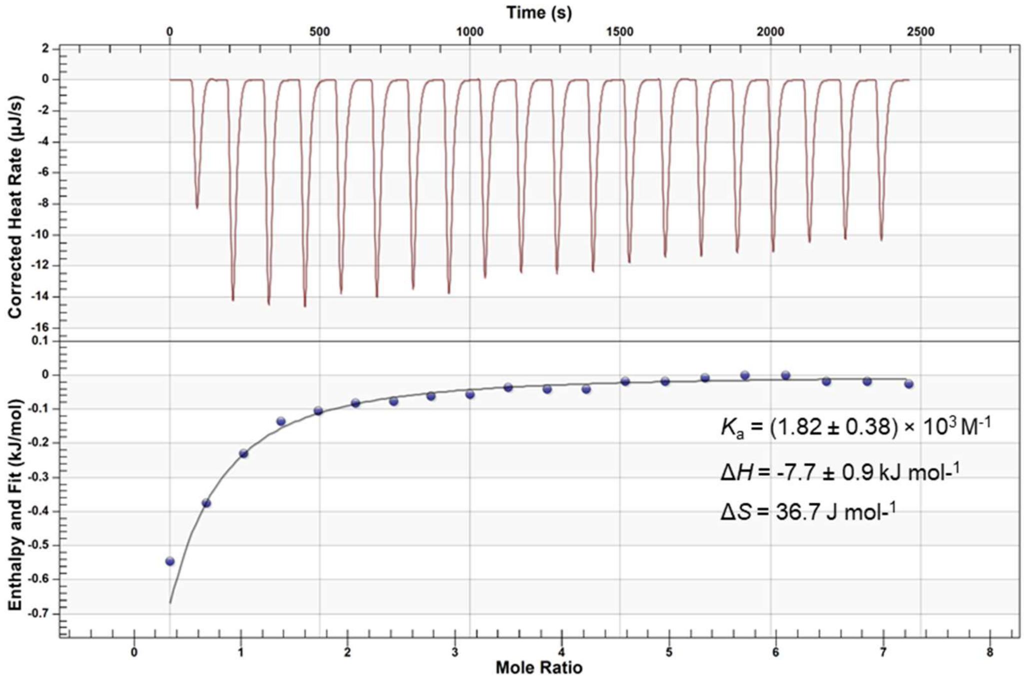

Figure S57. ITC titration plots and fitted curves (25 °C, MeCN) obtained by titration of **G1** into Λ_4-**1**.

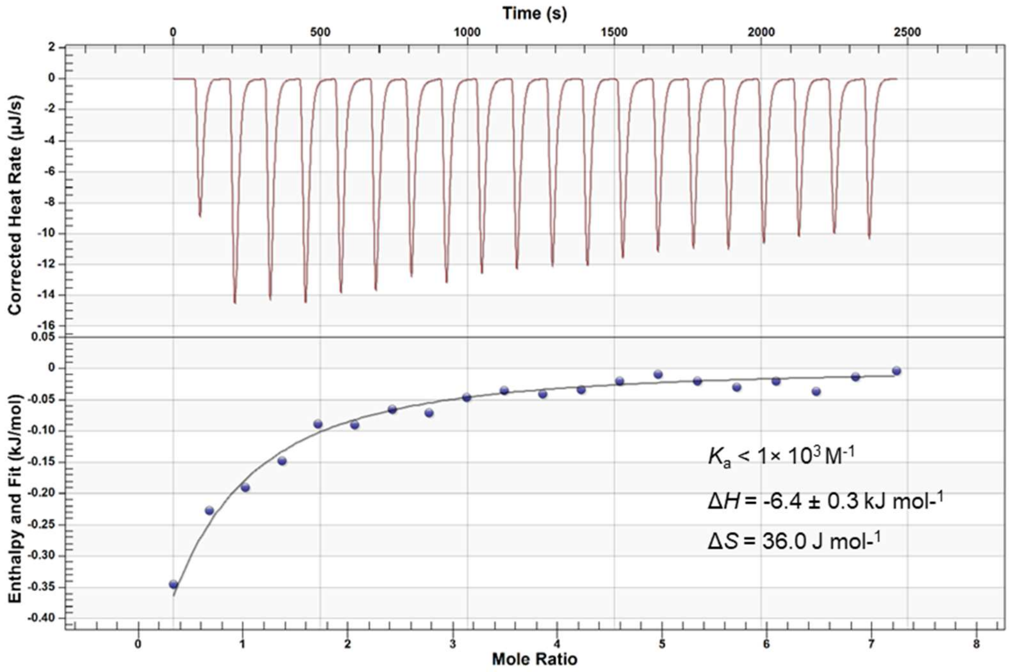

Figure S58. ITC titration plots and fitted curves (25 °C, MeCN) obtained by titration of **G1** into **2**.

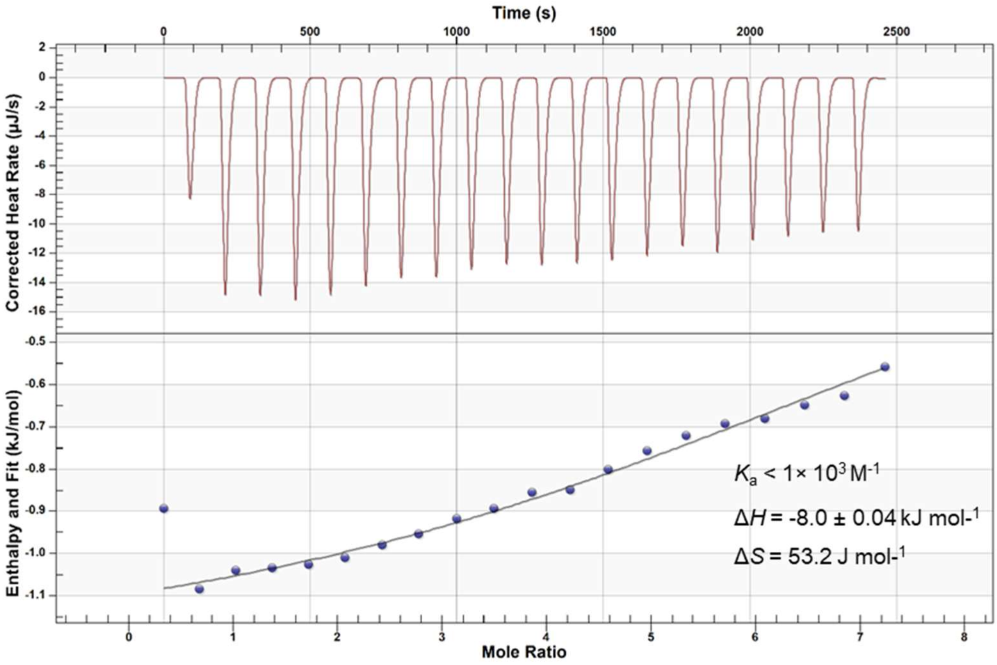

Figure S59. ITC titration plots and fitted curves (25 °C, MeCN) obtained by titration of **G2** into Δ_4-**1**.

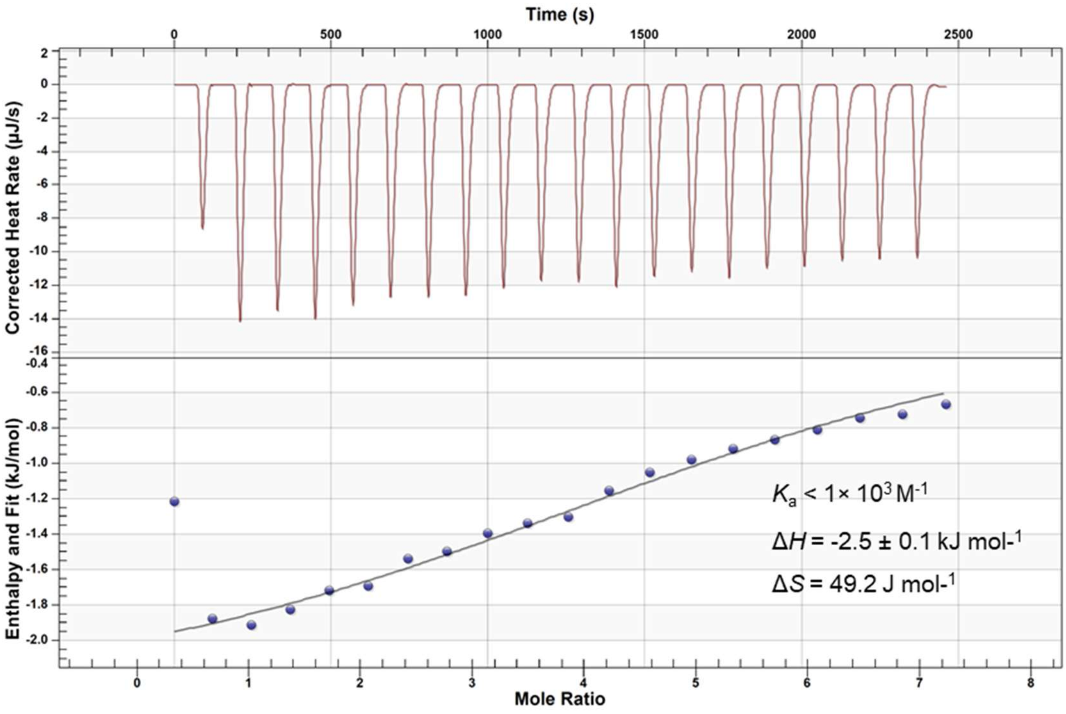

Figure S60. ITC titration plots and fitted curves (25 °C, MeCN) obtained by titration of **G2** into Λ_4-**1**.

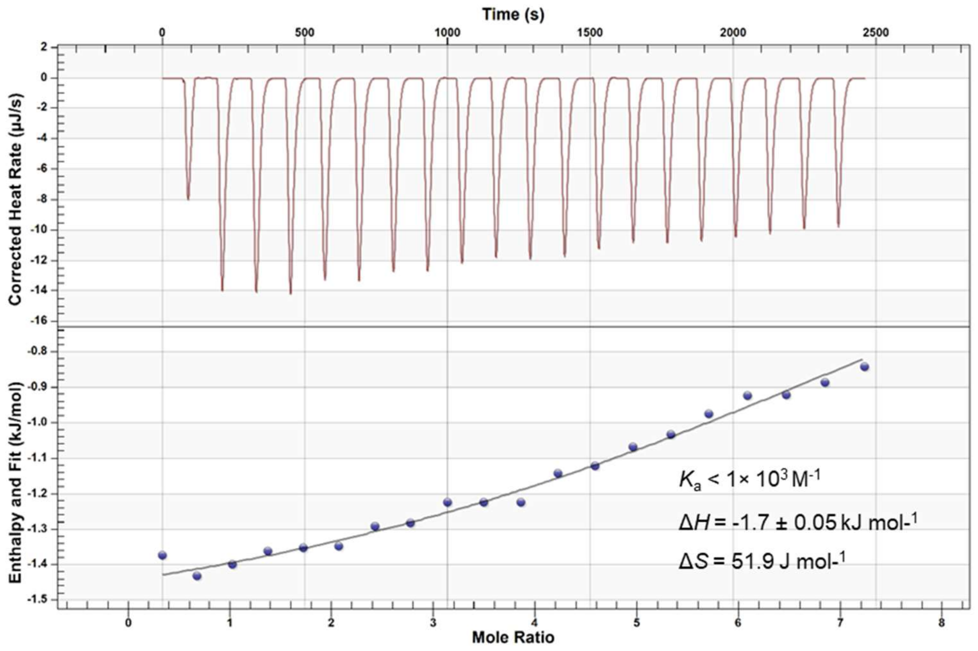

Figure S61. ITC titration plots and fitted curves (25 °C, MeCN) obtained by titration of **G2** into **2**.

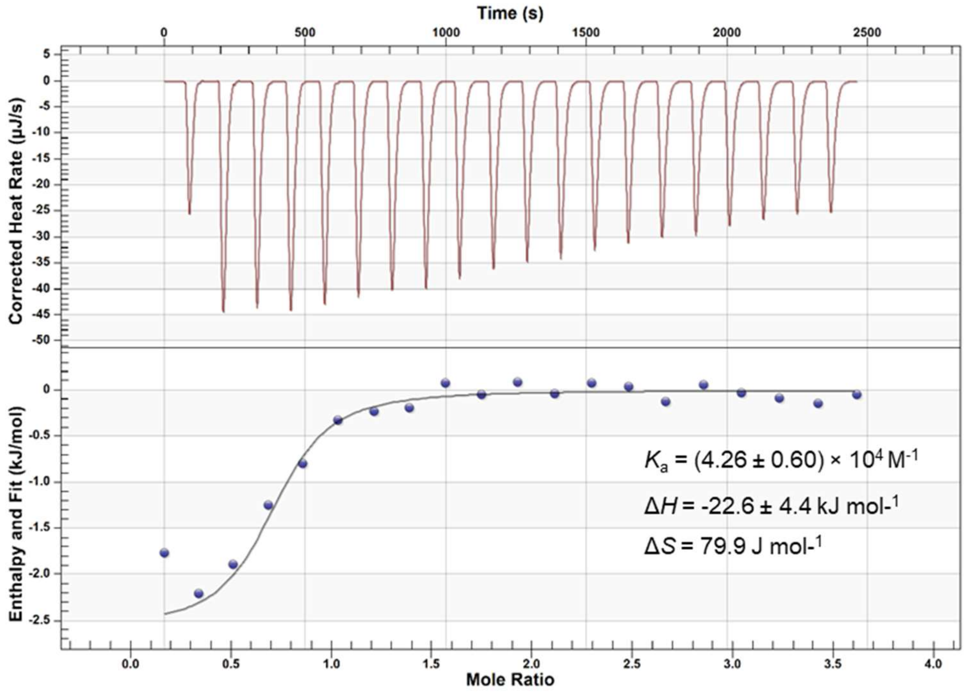

Figure S62. ITC titration plots and fitted curves (25 °C, MeCN) obtained by titration of **G3** carboxylate into *Δ*$_4$-**1**.

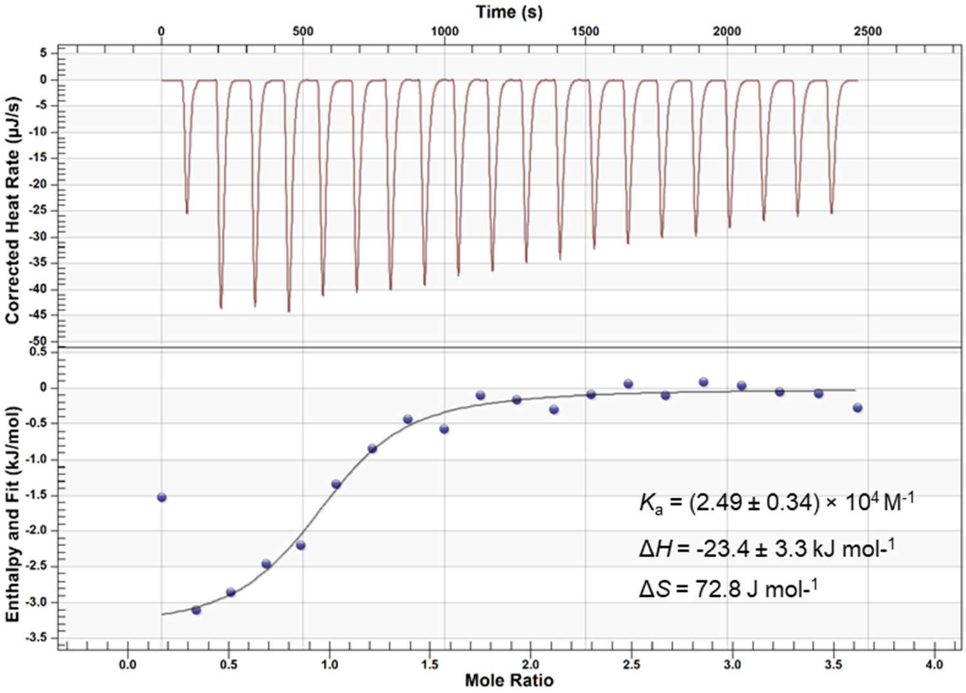

Figure S63. ITC titration plots and fitted curves (25 °C, MeCN) obtained by titration of **G3** carboxylate into Λ_4-**1**.

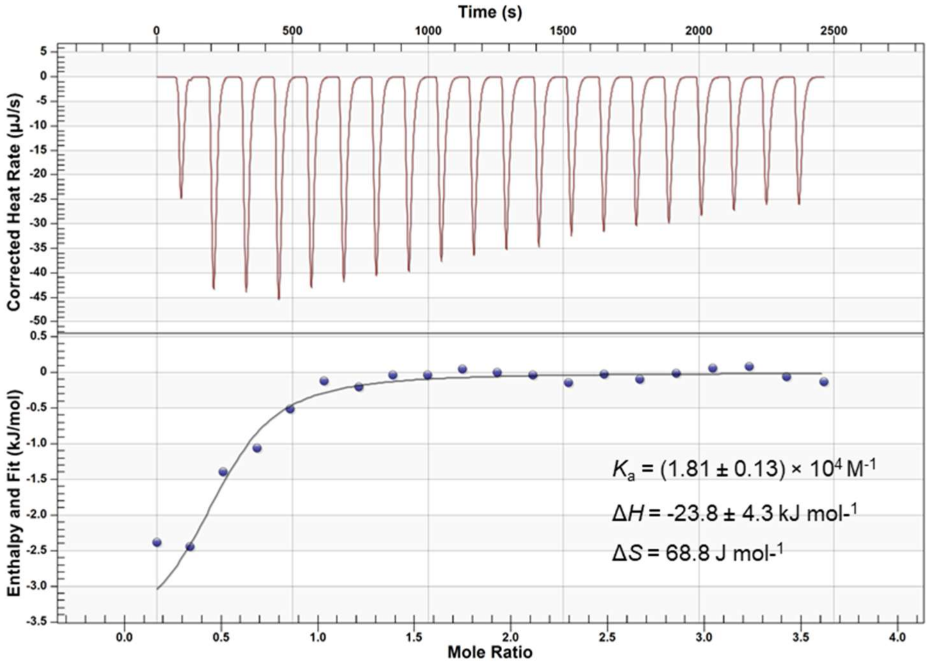

Figure S64. ITC titration plots and fitted curves (25 °C, MeCN) obtained by titration of **G3** carboxylate into **2**.

5 CPL Studies of Host-Guest Systems

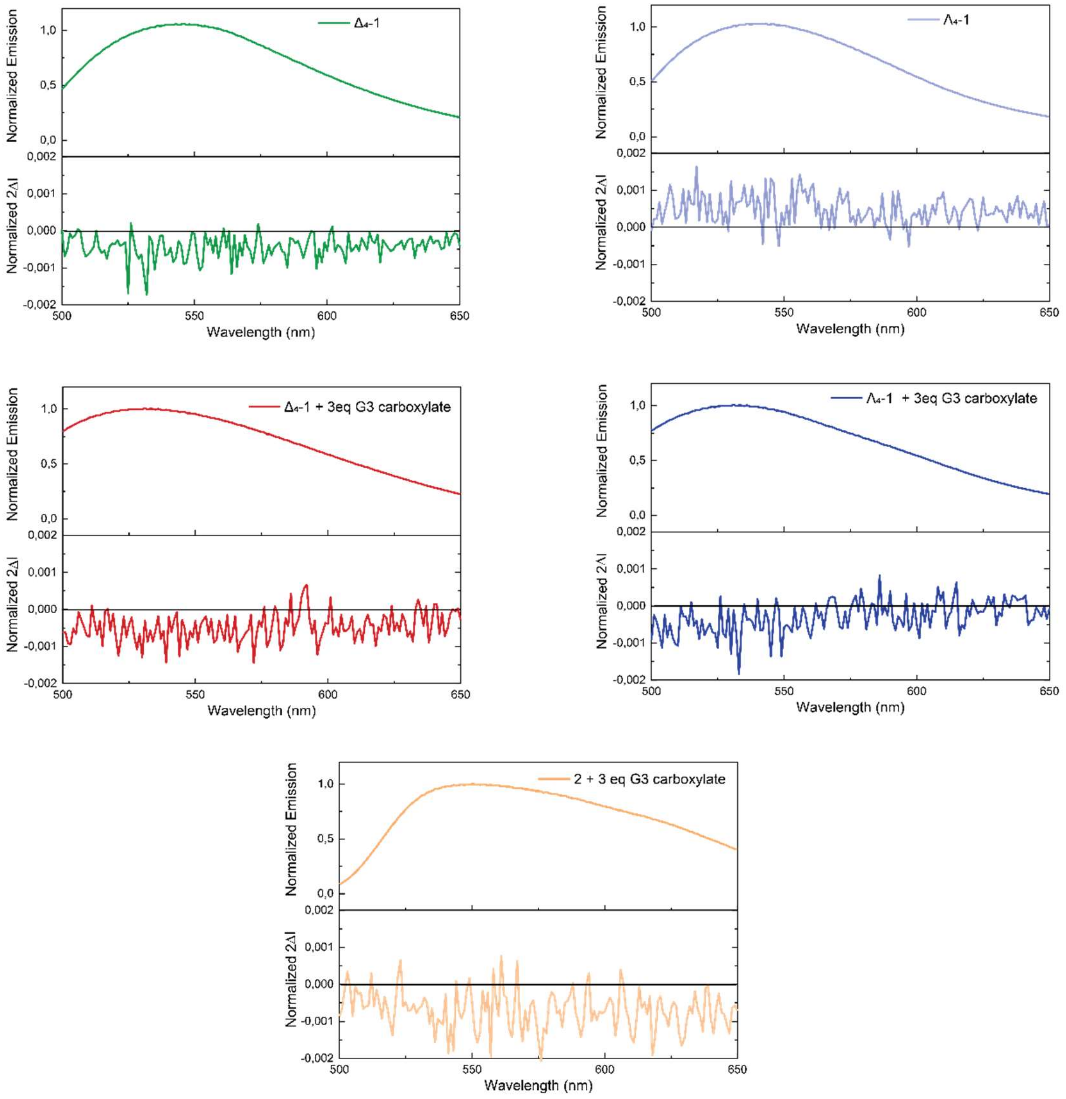

Figure S65. CPL (bottom) and emission (top) spectra of the Zn_4L_4 cages and their host-guest complexes (λ_{ex} = 385 nm for **2** + **G3** carboxylate, λ_{ex} = 425 nm for **1** and **1** + **G3** carboxylate, 0.60 mM of cage, 10 mm path-length cuvette, CD_3CN, 25 °C).

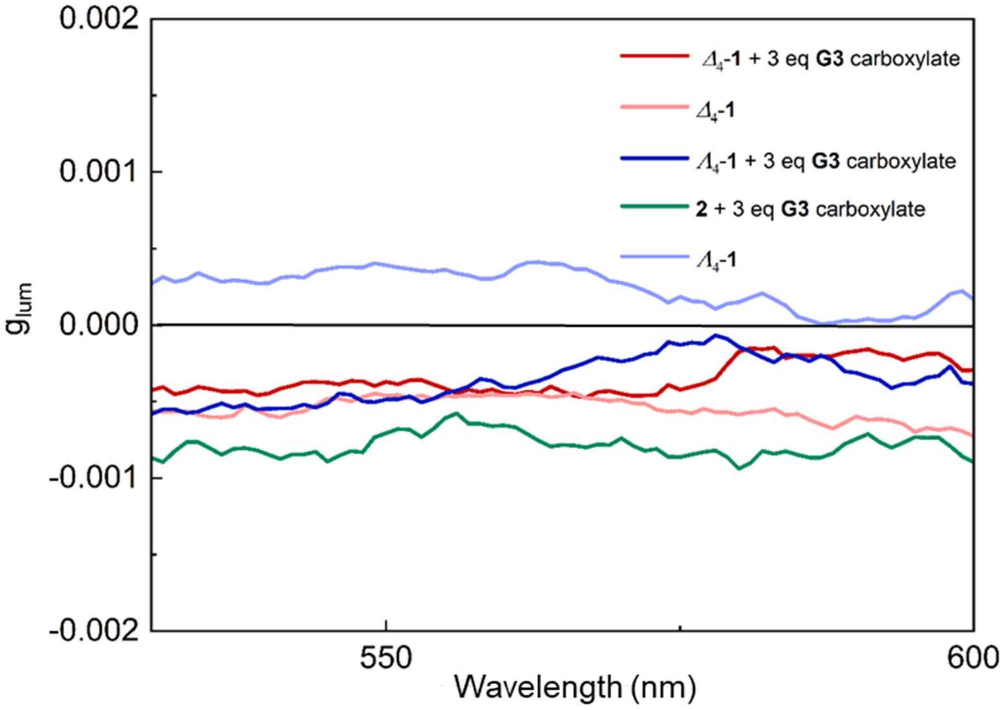

Figure S66. CPL spectra of the Zn_4L_4 cages and their host-guest complexes after smoothening of the g_{lum} curves using the machine software functionality.

6 Volume Calculations

In order to determine the available void spaces within the structures of the Zn_4L_4 cages, MoloVol[4] calculations based on the crystal structures were performed. A probe with a radius of 1.6 Å was employed. The standard parameters are tabulated below, and the results are shown below.

Probe mode: one probe

Probe radius: 1.5 Å

Grid resolution: 0.1 Å

Optimization depth: 4

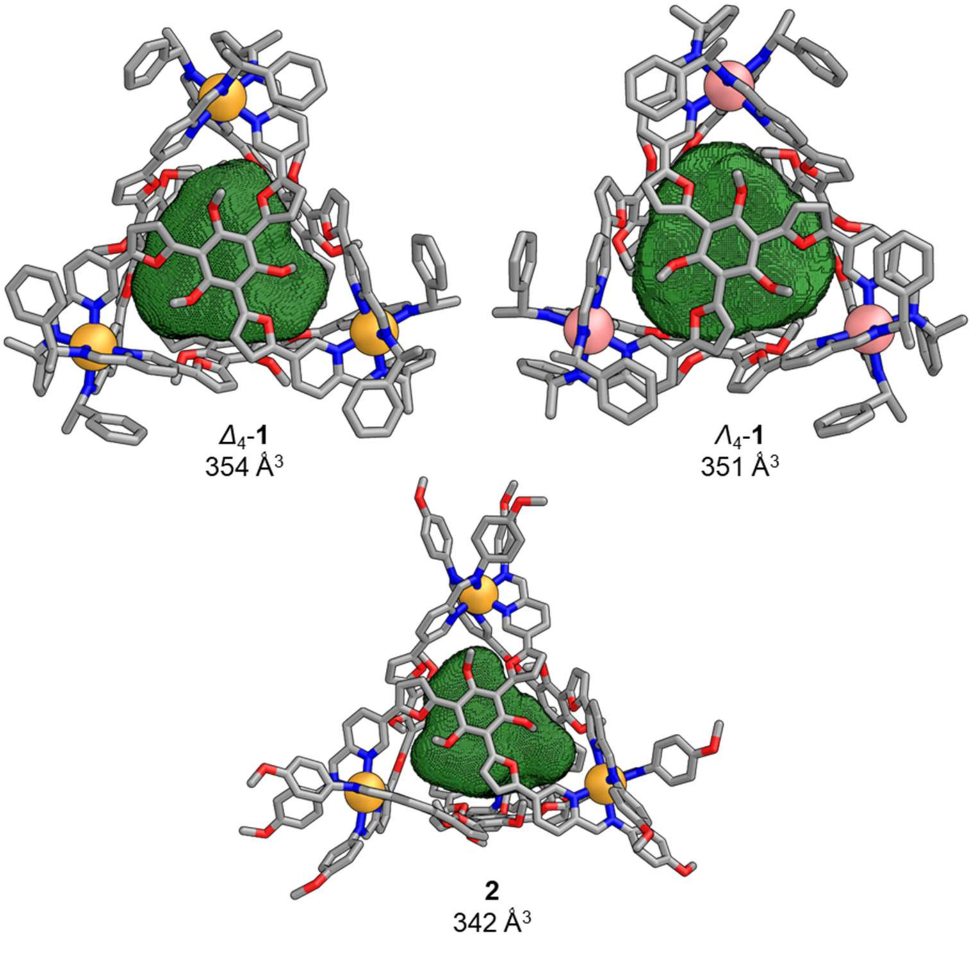

Figure S67. MoloVol-calculated void space (green mesh) within the crystal structure of the $Zn^{II}_4L_4$ Cages.

7 Crystal Structures of $Zn^{II}_4L_4$ Cages

Crystals of the $Zn^{II}_4L_4$ cages were grown by diffusion of *i*Pr_2O into an acetonitrile solution of the cage. Due to small block or thin plate shaped crystals, the analysis required cryogenic handling and highly brilliant synchrotron radiation. Hence, diffraction data of most of the supramolecular assemblies was collected during four beamtime shifts at macromolecular synchrotron beamline P11, PETRA III, DESY.[5] Counterion and solvent flexibility required carefully adapted macromolecular refinement protocols employing geometrical restraint dictionaries, similarity restraints and restraints for anisotropic displacement parameters (ADPs).

Table S3. Crystal data and structure refinement for supramolecular assemblies *Δ*$_4$-**1** and *Λ*$_4$-**1**.

**Compound**	*Λ*$_4$-**1**	*Δ*$_4$-**1**
**CIF ID**	xwc2rp	xwc2sp
**CCDC number**	2309382	2309383
Empirical formula	$C_{125.25}H_{105.75}F_{21}N_{12}O_{12}Sb_{3.50}Zn_2$	$C_{126}H_{108}F_{21}N_{12}O_{12}Sb_{3.50}Zn_2$
Formula weight	2926.83	2938.10
Temperature [K]	100(2)	100(2)
Crystal system	trigonal	trigonal
Space group (number)	$R3:H$ (146)	$R3:H$ (146)
a [Å]	23.30(6)	23.283(6)
b [Å]	23.30(6)	23.283(6)
c [Å]	91.8(2)	93.16(2)
α [°]	90	90
β [°]	90	90
γ [°]	120	120
Volume [Å^3]	43139(226)	43736(24)
Z	12	12
ρ_{calc} [gcm^{-3}]	1.352	1.339
μ [mm^{-1}]	2.849	2.811
F(000)	17577	17658
Crystal size [mm^3]	0.025×0.025×0.025	0.050×0.040×0.010

Crystal colour	yellow	yellow
Crystal shape	block	block
Radiation	synchrotron (λ=1.03318 Å)	synchrotron (λ=1.03318 Å)
2θ range [°]	1.94 to 46.83 (1.30 Å)	1.91 to 50.10 (1.22 Å)
Index ranges	$-17 \le h \le 16$<br>$-17 \le k \le 16$<br>$-70 \le l \le 70$	$-18 \le h \le 18$<br>$-19 \le k \le 19$<br>$-75 \le l \le 75$
Reflections collected	22685	51796
Independent reflections	8930<br>R_{int} = 0.0863<br>R_{sigma} = 0.1038	10914<br>R_{int} = 0.0781<br>R_{sigma} = 0.0612
Completeness	99.7 %	97.7 %
Data / Restraints / Parameters	8930/4341/2187	10914/4593/2259
Goodness-of-fit on F^2	1.454	1.249
Final *R* indexes [$I \ge 2\sigma(I)$]	R_1 = 0.1391<br>wR_2 = 0.3553	R_1 = 0.1025<br>wR_2 = 0.2606
Final *R* indexes [all data]	R_1 = 0.1544<br>wR_2 = 0.3743	R_1 = 0.1094<br>wR_2 = 0.2700
Largest peak/hole [eÅ^{-3}]	1.29/-0.50	0.80/-0.38
Flack X parameter	0.164(16)	0.162(6)

Table S4. Crystal data and structure refinement for supramolecular assembly **2**.

**Compound**	**2**
**CIF ID**	xwc1
**CCDC number**	2309384
Empirical formula	$C_{240}H_{191}F_3N_{24}O_{36}P_{0.50}Zn_4$
Formula weight	4321.12
Temperature [K]	293(2)
Crystal system	monoclinic
Space group (number)	$C2/c$ (15)
a [Å]	43.15(3)
b [Å]	39.76(2)
c [Å]	37.69(2)
α [°]	90
β [°]	91.397(7)
γ [°]	90
Volume [Å^3]	64641(66)
Z	8
ρ_{calc} [gcm^{-3}]	0.888
μ [mm^{-1}]	0.929
F(000)	17932
Crystal size [mm^3]	0.075×0.075×0.01
Crystal colour	yellow

Crystal shape	plate
Radiation	synchrotron (λ=1.0331 Å)
2θ range [°]	2.02 to 48.40 (1.26 Å)
Index ranges	$-33 \leq h \leq 33$<br>$-28 \leq k \leq 29$<br>$-29 \leq l \leq 29$
Reflections collected	111053
Independent reflections	16251<br>R_{int} = 0.0485<br>R_{sigma} = 0.0272
Completeness	96.0 %
Data / Restraints / Parameters	16251/5888/2799
Goodness-of-fit on F^2	2.037
Final *R* indexes<br>[$I \geq 2\sigma(I)$]	R_1 = 0.1524<br>wR_2 = 0.4453
Final *R* indexes<br>[all data]	R_1 = 0.1780<br>wR_2 = 0.4688
Largest peak/hole [eÅ^{-3}]	0.79/-0.59

7.1 Crystal Structure of Δ_4-**1**

Yellow block-shaped crystals of Δ_4-**1** were grown by slow vapor diffusion of diisopropyl ether (*i*Pr_2O) into an acetonitrile solution of Δ_4-**1** at room temperature. Single crystals in mother liquor were pipetted onto a glass slide containing NVH oil. To avoid collapse of the crystal lattice, several crystals were quickly mounted on nylon micro loops and immediately flash-cooled in liquid nitrogen. Crystals were stored at cryogenic temperature in dry shippers, in which they were safely transported to macromolecular beamline P11 at Petra III,[5] DESY, Hamburg, Germany. A wavelength of λ = 1.0331 Å was chosen using a liquid N_2 cooled double crystal monochromator. Single crystal X-ray diffraction data was collected at 100(2) K on a single axis goniometer, equipped with an Oxford Cryostream 800 and an Eiger2x 16M detector. 3600 diffraction images were collected in a 360° φ sweep at a detector distance of 154 mm, 100% filter transmission, 0.1° step width and 0.1 seconds exposure time per image. Data integration and reduction were undertaken using XDS.[6] The structure was solved by intrinsic phasing/direct methods using SHELXT[7] and refined with SHELXL[8] using 22 CPU cores for full-matrix least-squares routines on F^2 and ShelXle[9] as a graphical user interface and the DSR program plugin was employed for modeling.[10,11]

Specific refinement details

Stereochemical restraints for the organic ligands (residue XW2), and hexafluoroantimonate counter ions (residue SB6) were generated by the GRADE program using the GRADE Web Server (http://grade.globalphasing.org) and applied in the refinement. A GRADE dictionary for SHELXL contains target values and standard deviations for 1,2-distances (DFIX) and 1,3-distances (DANG), as well as restraints for planar groups (FLAT). All displacements for non-hydrogen atoms were refined anisotropically. The refinement of ADPs for carbon, nitrogen and oxygen atoms was enabled by a combination of similarity restraints (SIMU) and rigid bond restraints (RIGU).[12] The contribution of the electron density from disordered counterions and solvent molecules, which could not be modelled with discrete atomic positions were handled using the SQUEEZE[13] routine in PLATON.[14] The solvent mask file (.fab),

computed by PLATON, was included in the SHELXL refinement via the ABIN instruction leaving the measured intensities untouched.

The compound crystalized in trigonal space group $R3{:}H$ (no. 146). The asymmetric unit cell contains two times one-third of a cage, combining one full XW2 ligand and one third of a XW2 ligand, as well as six hexafluoroantimonate counter anions. Two of the six counter-anions were modelled as disordered over two conformations using free individual variables for refinement of occupancy factors, and two other counter-anions were modelled on special positions (threefold rotoinversion axes).

The Flack parameter according to Parson's method is **0.162(6)**. While the low resolution and high solvent content did not allow us to get a lower value, the presence of the known *S* chiral center is proof enough of the correct stereochemistry of the model.

7.2 Crystal Structure of *Λ*$_4$-1

Yellow block-shaped crystals of *Λ*$_4$-**1** were grown by slow vapor diffusion of diisopropyl ether (*i*Pr_2O) into an acetonitrile solution of *Λ*$_4$-**1** at room temperature. Single crystals in mother liquor were pipetted onto a glass slide containing NVH oil. To avoid collapse of the crystal lattice, several crystals were quickly mounted on nylon micro loops and immediately flash-cooled in liquid nitrogen. Crystals were stored at cryogenic temperature in dry shippers, in which they were safely transported to macromolecular beamline P11 at Petra III,[5] DESY, Hamburg, Germany. A wavelength of λ = 1.0331 Å was chosen using a liquid N_2 cooled double crystal monochromator. Single crystal X-ray diffraction data was collected at 100(2) K on a single axis goniometer, equipped with an Oxford Cryostream 800 and an Eiger2x 16M detector. 1800 diffraction images were collected in a 180° φ sweep at a detector distance of 154 mm, 100% filter transmission, 0.1° step width and 0.2 seconds exposure time per image. Data integration and reduction were undertaken using XDS.[6] The structure was solved by intrinsic phasing/direct methods using SHELXT[7] and refined with SHELXL[8] using 22 CPU cores for full-matrix least-squares routines on F^2 and ShelXle[9] as a graphical user interface and the DSR program plugin was employed for modeling.[10,11]

Specific refinement details

Stereochemical restraints for the organic ligands (residue XW2), and hexafluoroantimonate counter ions (residue SB6) were generated by the GRADE program using the GRADE Web Server (http://grade.globalphasing.org) and applied in the refinement. A GRADE dictionary for SHELXL contains target values and standard deviations for 1,2-distances (DFIX) and 1,3-distances (DANG), as well as restraints for planar groups (FLAT). All displacements for non-hydrogen atoms were refined anisotropically. The refinement of ADPs for carbon, nitrogen and oxygen atoms was enabled by a combination of similarity restraints (SIMU) and rigid bond restraints (RIGU).[12] The contribution of the electron density from disordered counterions and solvent molecules, which could not be modeled with discrete atomic positions were handled using the SQUEEZE[13] routine in PLATON.[14] The solvent mask file (.fab),

computed by PLATON, was included in the SHELXL refinement via the ABIN instruction leaving the measured intensities untouched.

The compound crystalized in trigonal space group $R3{:}H$ (no. 146). The asymmetric unit cell contains two times one-third of a cage, combining one full XW2 ligand and one third of a XW2 ligand, as well as six hexafluoroantimonate counter anions. One of the six counter-anions were modelled as disordered over two conformations using free individual variables for refinement of occupancy factors, and two other counter-anions were modelled on special positions (threefold rotoinversion axes).

Due to instability of the model, carbon C8 of a methoxy group of a XW2 ligand (residue 2) could not be modelled and the adjacent O7 had to be left naked. It is likely that the region presents some degree of disorder, and due to the low resolution of the data and low redundancy it not possible to fully model the disordered positions.

The Flack parameter according to Parson's method is **0.164(16)**. While the low resolution and high solvent content did not allow us to get a lower value, the presence of the known *R* chiral center is proof enough of the correct stereochemistry of the model.

7.3 Crystal Structure of 2

Yellow block-shaped crystals of **2** were grown by slow vapor diffusion of diisopropyl ether (*i*Pr_2O) into an acetonitrile solution of **2** at room temperature. Single crystals in mother liquor were pipetted onto a glass slide containing NVH oil. To avoid collapse of the crystal lattice, several crystals were quickly mounted on nylon micro loops and immediately flash-cooled in liquid nitrogen. Crystals were stored at cryogenic temperature in dry shippers, in which they were safely transported to macromolecular beamline P11 at Petra III,[5] DESY, Hamburg, Germany. A wavelength of λ = 1.0331 Å was chosen using a liquid N_2 cooled double crystal monochromator. Single crystal X-ray diffraction data was collected at 100(2) K on a single axis goniometer, equipped with an Oxford Cryostream 800 and an Eiger2x 16M detector. 3600 diffraction images were collected in a 360° φ sweep at a detector distance of 154 mm, 100% filter transmission, 0.1° step width and 0.2 seconds exposure time per image. Data integration and reduction were undertaken using XDS.[6] The structure was solved by intrinsic phasing/direct methods using SHELXT[7] and refined with SHELXL[8] using 22 CPU cores for full-matrix least-squares routines on F^2 and ShelXle[9] as a graphical user interface and the DSR program plugin was employed for modeling.[10,11]

Specific refinement details

Stereochemical restraints for the organic ligands (residue XW1), and hexafluorophosphate counter ions (residue PF6) were generated by the GRADE program using the GRADE Web Server (http://grade.globalphasing.org) and applied in the refinement. A GRADE dictionary for SHELXL contains target values and standard deviations for 1,2-distances (DFIX) and 1,3-distances (DANG), as well as restraints for planar groups (FLAT). All displacements for non-hydrogen atoms were refined anisotropically. The refinement of ADPs for carbon, nitrogen and oxygen atoms was enabled by a combination of similarity restraints (SIMU) and rigid bond restraints (RIGU).[12] The contribution of the electron density from disordered counterions and solvent molecules, which could not be modeled with discrete atomic positions were handled using the SQUEEZE[13] routine in PLATON.[14] The solvent mask file (.fab),

computed by PLATON, was included in the SHELXL refinement via the ABIN instruction leaving the measured intensities untouched.

The compound crystalized in monoclinic space group $C2/c$ (no. 15). The asymmetric unit cell contains one full cage and one half hexafluorophosphate anion. Three methoxy groups at the central phenyl ring of ligand XW1 were were modelled as disordered over two conformations using free individual variables for refinement of occupancy factors.

8 References

1. Y. Tamura, H. Takezawa, M. Fujita, *J. Am. Chem. Soc.* **2020**, *142*, 5504–5508.
2. A. Ojida, T. Sakamoto, M.-a. Inoue, S.-h. Fujishima, G. Lippens, I. Hamachi, *J. Am. Chem. Soc.* **2009**, *131*, 6543–6548.
3. S. E. Howson, L. E. N. Allan, N. P. Chmel, G. J. Clarkson, R. J. Deeth, A. D. Faulkner, D. H. Simpson, P. Scott, *Dalton Trans.* **2011**, *40*, 10416–10433.
4. J. B. Maglic, R. Lavendomme, *J. Appl. Cryst.* **2022**, *55*, 1033–1044.
5. A. Burkhardt, T. Pakendorf, B. Reime, J. Meyer, P. Fischer, N. Stübe, S. Panneerselvam, O. Lorbeer, K. Stachnik, M. Warmer, P. Rödig, D. Göries, A. Meents, *Eur. Phys. J. Plus* **2016**, *131*, 56.
6. W. Kabsch, *Acta Crystallogr. D Biol. Crystallogr.* **2010**, *66*, 125–132.
7. G. M. Sheldrick, *Acta Crystallogr. A Found. Adv.* **2015**, *71*, 3–8.
8. G. M. Sheldrick, *Acta Crystallogr. C. Struct. Chem.* **2015**, *71*, 3–8.
9. C. B. Hübschle, G. M. Sheldrick, B. Dittrich, *J. Appl. Crystallogr.* **2011**, *44*, 1281–1284.
10. D. Kratzert, J. J. Holstein, I. Krossing, *J. Appl. Crystallogr.* **2015**, *48*, 933–938.
11. D. Kratzert, I. Krossing, *J. Appl. Crystallogr.* **2018**, *51*, 928–934.
12. A. Thorn, B. Dittrich, G. M. Sheldrick, *Acta Crystallogr. A Found. Adv.* **2012**, *68*, 448–451.
13. A. L. Spek, *Acta Crystallogr. C. Struct. Chem.* **2015**, *71*, 9–18.
14. A. L. Spek, *Acta Crystallogr. D. Biol. Crystallogr.* **2009**, *65*, 148–155.
